# Tissue-Like Multicellular Development Triggered by Mechanical Compression in Archaea

**DOI:** 10.1126/science.adu0047

**Published:** 2025-04-03

**Authors:** Theopi Rados, Olivia S. Leland, Pedro Escudeiro, John Mallon, Katherine Andre, Ido Caspy, Andriko von Kügelgen, Elad Stolovicki, Sinead Nguyen, Inés Lucía Patop, L. Thiberio Rangel, Sebastian Kadener, Lars D. Renner, Vera Thiel, Yoav Soen, Tanmay A.M. Bharat, Vikram Alva, Alex Bisson

**Affiliations:** 1 Brandeis University, Department of Biology; Waltham, MA, USA; 2 Department of Protein Evolution, Max Planck Institute for Biology Tübingen; Tübingen, Germany; 3 Structural Studies Division, MRC Laboratory of Molecular Biology; Cambridge, United Kingdom; 4 Department of Biomolecular Sciences, Weizmann Institute of Science; Israel; 5 Department of Earth, Atmospheric, & Planetary Sciences, Massachusetts Institute of Technology; Cambridge, MA, USA; 6 Leibniz Institute of Polymer Research and the Max Bergmann Center of Biomaterials; Dresden, Germany; 7 Department of Microorganisms, Leibniz Institute DSMZ – German Collection of Microorganisms and Cell Cultures GmbH; Germany

## Abstract

The advent of clonal multicellularity is a critical evolutionary milestone, seen often in eukaryotes, rarely in bacteria, and only once observed in archaea. We show that uniaxial compression induces clonal multicellularity in haloarchaea, forming tissue-like structures. These archaeal tissues are mechanically and molecularly distinct from their unicellular lifestyle, mimicking several eukaryotic features. Archaeal tissues undergo a multinucleate stage followed by tubulin-independent cellularization, orchestrated by active membrane tension at a critical cell size. After cellularization, tissue junction elasticity becomes akin to animal tissues, giving rise to two cell types—peripheral (Per) and central scutoid (Scu) cells—with distinct actin and protein glycosylation polarity patterns. Our findings highlight the potential convergent evolution of a biophysical mechanism in the emergence of multicellular systems across domains of life.

Multicellularity has evolved multiple times across the tree of life, fundamentally reshaping Earth's biosphere. Comparative studies across these independent transitions have revealed common selective benefits, including increased size, enhanced mechanical strength, and cellular differentiation—principles later confirmed through experimental evolution (1–4). While well-documented in eukaryotes and prokaryotes, the extent to which clonal multicellularity contributes to the emergence of structural and functional complexity in bacteria and archaea remains unclear. Once mistaken for bacteria due to their lack of nuclei, archaea are now recognized as a monophyletic group with eukaryotes (5). Most archaea lack a rigid cell wall and are encapsulated by a proteinaceous surface monolayer (S-layer), a 2D paracrystalline lattice composed of glycoproteins (6, 7). While the archaeal envelope structure is thought to make cells mechanically vulnerable, it also facilitates close interactions between cells, such as cell-cell contact and fusion, which may have played a role in the emergence of eukaryotes (8, 9). However, the evolution of mechanosensory responses driven by the lack of a rigid cell wall remains elusive due to the scarcity of in vivo studies. The unique combination of genetic and biophysical traits prompted us to investigate the mechanobiology of archaeal cells, leading to the serendipitous discovery of a reversible, clonal tissue-like multicellular developmental program.

## Uniaxial compression gives rise to clonal, tissue-like multicellularity

**Fig. 1:**
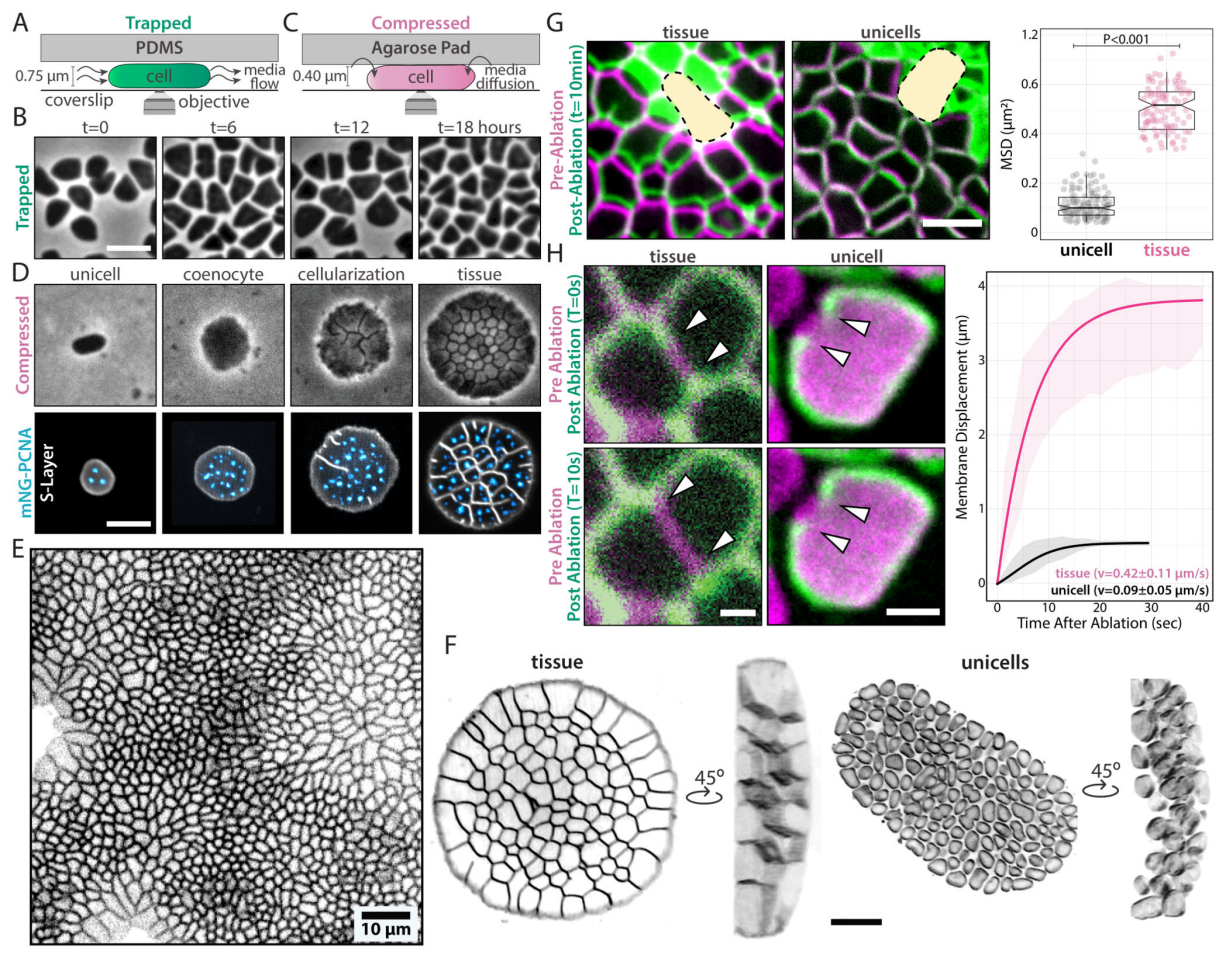
Uniaxial compression triggers multicellular development in *Hfx. volcanii*. **(A)** Schematic of cells trapped and **(B)** phase-contrast time-lapses of cells growing in in the ArcCell microfluidic device. **(C)** Schematic of compressed cells under 2.5% agarose pads. **(D)** Phase-contrast (top row) and spinning-disk confocal (bottom row) time-lapses of compressed cells across ~6 generations. msfGFP-PCNA foci (blue) represent replication sites. **(E)** Stretched and compressed areas comprised in large monolayers of epithelia-like tissues. **(F)** 3D-SoRa microscopy images of a tissue (top) and unicells (bottom). **(G)** Laser ablation of tissue regions. False-colored overlays of tissues before (magenta) and 10 minutes after (green) ablation. Yellow areas indicate the ablated area. Directional motion from cells was calculated from MSD curves. **(H)** Laser ablation of cell membranes. False-colored overlays of tissues and cells before (magenta) and after (green) ablation. White arrowheads indicate the membrane recoil retraction. Unless specified, scale bars represent 2 μm.

To gain insights into the mechanobiology of archaeal cells, we performed confinement experiments with the salt-loving *Haloferax volcanii* (*Hvo*), leveraging its straightforward cultivation and genetics (10). First, we established a baseline for mechanically unperturbed haloarchaeal cells trapped within ArcCell, a custom microfluidic device (Fig. 1A and S1A). Cells growing in ArcCell showed cell morphologies comparable to those in bulk liquid cultures, indicating no mechanical stress (Fig. 1B and S1B; Movie S1). Next, we imaged cells under agarose pads, a standard technique for microbial immobilization (Fig. 1C). Unlike in ArcCell, agarose pads deformed cells within a single generation (~2.5 hours) at the lowest agarose concentrations, making pad immobilization incompatible with prolonged imaging (Fig. S1B, Movie S1). To quantify the compressive forces deforming cells, we measured the pad elasticity using dynamic mechanical analysis (DMA) (11). The storage moduli of 0.25-3.5% pads revealed that resistance forces of ~10 kPa are sufficient to deform *Hvo* (Fig. S1C). These values suggest *Hvo* cells may have viscoelastic properties close to eukaryotic cells, such as amoeba and mammalian cells, but orders of magnitude lower than most cell-walled organisms (12, 13).

Given the mechanical sensitivity of haloarchaeal cells under pads, we tested the responses of *Hvo* to compressive forces above 100 kPa, closer to their natural habitat, such as the human gut and salt ponds (14–16). Following compression under pads with agarose concentrations of 1.5% and higher, cells stopped dividing but continued to grow (Fig. 1D, 2^nd^ panel). After ~12 hours, cellularization occurred via simultaneous septation events (Fig. 1D, 3^rd^ panel), resulting in epithelial-like monolayer structures (Fig. 1E; Movie S2). These tissue-like ensembles resemble radial tessellation patterns in leaf tissues and multicellular green algae (17, 18). The morphological development before cellularization also resembles the coenocytic phase of chytrids and chicken embryogenesis, where cells multiply their nuclei without cell division (19, 20). Time-lapses of cells expressing mNeonGreen-PCNA (DNA sliding clamp) showed continuous replication during development, supporting a coenocytic-like phase preceding cellularization (Fig. 1D; Movie S3).

Animal and plant cells often sense and activate biochemical pathways in response to surface curvatures and material properties (21, 22). To test the influence of the pad's stiffness, *Hvo* cells were immobilized under and on top of the same 2.5% agarose pad. As a result, tissues were observed exclusively under the pads (Fig. S2A), suggesting stiffness alone is not sufficient to induce multicellularity. Furthermore, cells compressed by different bilayer ″cake″ pad setups developed into tissue-like structures only when under compression by at least one stiff surface, ruling out a specific role of the coverslip other than providing a rigid surface for compression (Fig. S2B and S2C). Finally, tissue formation is independent of gravity (Fig. S3A), pad mass, or thickness (Fig. S3B) and consistently initiated at the same coenocytic area regardless of pad density (Fig. S3C). These analogous features suggest archaeal tissue development represents an evolved biological program response to compression.

To determine if cells within multicellular structures retain their S-layer lattice, we cryo-fixed cells from mechanically sheared tissues and imaged individual cells by electron cryotomography (cryo-ET) (Fig. S4A). Concomitantly with live-cell staining of glycoproteins (Fig. S4B), we concluded that archaeal tissues preserve S-layer material in their intercellular spaces.

Next, we explored the possibility that tissues arise from cell compaction. 3D imaging revealed extracellular spaces between most unicells but none within tissues (Fig. 1F; Movie S4). To probe physical connectivity, we used laser ablation to wound areas at the center of the tissues. Following ablation, we observed directional movement of cells toward the wounds in tissues, but not in unicells, at speeds of 0.62±0.27 μm/min (Fig. 1G and S4C; Movie S5), comparable to those seen in wounded animal tissues, which can vary between 0.2-1.0 μm/min (23, 24). Since archaea lack canonical cytoskeleton motors like myosin (25), the synchronic cell migration suggests that archaeal and animal tissues have similar membrane elastic properties. We tested this idea by measuring the retraction rates after ablating the cell envelope in unicells and archaeal tissues, observing a membrane recoil in archaeal tissues (0.42±0.11μm/s) (Fig. 1H; Movie S6) similar to those reported in animal tissues (~0.3μm/s) (26). The apparent higher membrane tension in archaeal tissues compared to compacted unicells (0.09±0.05 μm/s) implies the presence of junctional load-bearing structures, placing archaeal tissues as a unique class of multicellularity, exhibiting material properties typical of eukaryotic tissues.

### Archaeal tissues are widespread in Haloarchaea and counter-correlate with biofilm production

To understand the evolutionary diversity of haloarchaeal tissues, we constructed a phylogenomic tree spanning 57 genera, representing all haloarchaeal orders (Data S1), imaging compressed cells from 52 species across 14 genera (Fig. S5). Regardless of cell size or growth rate (Fig. S6A), 61.6% of tested haloarchaeal species formed tissues, with at least one instance of tissue development or no multicellularity in every tested genus. Among non-forming tissue strains, we identified cases of cell death (Fig. S6B), shape deformation (Fig. S6C), and unnoticeable shape deformations under pads (Fig. S6D). We also observed 3 strains – *Htg. salina, Ncc. jeotgali*, and *Hka. jeotgali* – that exhibit aggregative multicellularity similar to *Methanosarcina*, the only previously reported multicellular archaeon (Fig. S6E) (27). These results suggest archaeal tissues emerged early in haloarchaeal evolution and remain dominant in the sampled diversity.

**Fig. 2:**
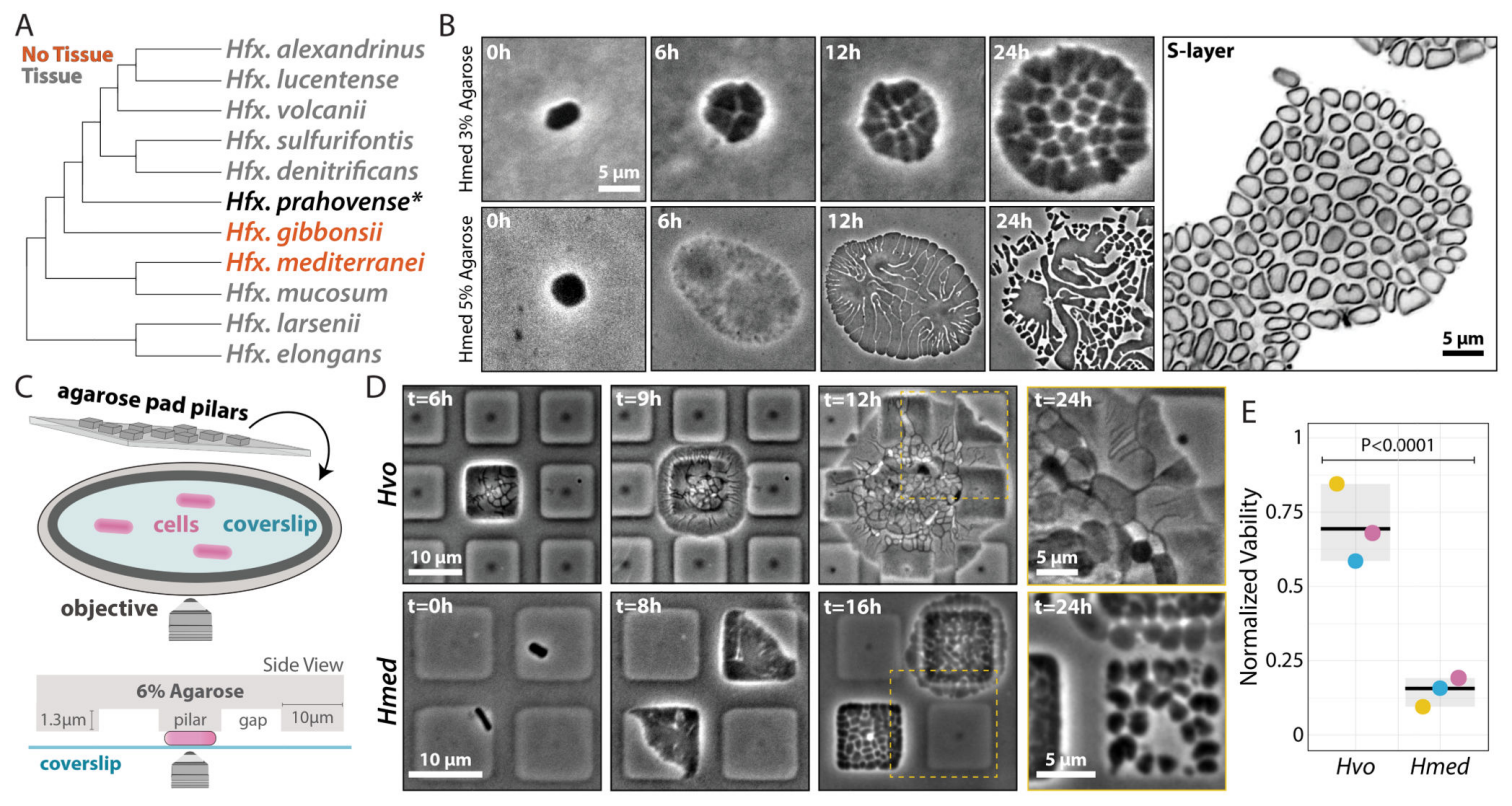
*Hfx. mediterranei* does not form tissues under compression. **(A)** Cladogram depicting evolutionary relationships between compressed *Haloferax* species. Gray- and red-labeled species represent cells that form or do not form tissues, respectively. *Hfx. prahovense* is marked with an asterisk as it develops to considerably larger, deformed tissues. For a comprehensive phylogenetic tree, see Supplementary Data S1. **(B)** Phase-contrast time-lapses (left) of *Hmed* growth under 3% (top) and 5% (bottom) agarose pads. (right) SoRa microscopy of *Hmed* growth after 24 hours under 3% agarose pads. **(C)** Cartoon representation of microfabricated pillars used in the intermittent compression experiments. **(D)** Phase-contrast time-lapses of *Hvo* (top) and *Hmed* (bottom) under micropillar devices. The 24-hour datapoint is represented as a zoom inlet from the yellow area in the previous timepoint. **(E)** Viability of *Hvo* tissues compared to *Hmed* cells under micropillars measured by colony formation unit (CFU). *Hvo* and *Hmed* viabilities were normalized by their respective liquid cultures.

Next, we focused on strains from the *Haloferax* genus, where *Hfx. prahovense* produced large, deformed tissue structures, whereas *Hfx. mediterranei* (*Hmed*) and *Hfx. gibbonsii* (*Hgib*) failed to develop, growing instead as stacked colonies (Fig. 2A and S7A; Movie S7). Although all three taxa are closely related, *Hmed* is placed on a long branch, suggesting an extended time for adaptation following the loss or gain of genetic material. Under higher compression (5% agarose pads), *Hmed* cells grew larger than *Hvo* coenocytes until they split and swarmed outwards (Fig. 2B; Movie S7). Since *Hmed*'s swarming-like motion resembles bacterial biofilm-dependent gliding (28), we questioned whether their extracellular matrix promoted survival under compression. Supporting this hypothesis, relative biofilm was at least twice as high in *Hmed* as in other *Hfx* strains (Fig. S7B). However, it remains unclear whether *Hmed* still hosts the (suppressed) genetic pathways for multicellularity or if it completely lost one or more required components. The diversification of tissue architecture suggests a shared multicellularity origin with occasional losses.

Despite sharing many similar “weed-like” traits, *Hmed* is still outcompeted in nature by other haloarchaea (29). To test if *Hmed*'s lack of multicellularity affects its fitness, we compressed cells under microfabricated pillars intercalated with ″relief″ zones (Fig. 2C). This setup allowed us to observe how these cells navigated mechanical ″escape room″ challenges, mimicking their natural habitat. While *Hvo* cells managed to propagate even with low initial cell numbers, *Hmed* showed a 4.3-fold decrease in viability compared to *Hvo* under pillars (Fig. 2D and E; Movie S8). In contrast, *Hvo* tissues showed only a 1.8-fold and 1.4-fold loss in viability compared to unicells and *Hvo* colonies from agar plates, respectively (Fig. S7C).

The survival rates between *Hvo* and *Hmed* suggest that tissues can revert to unicells. To observe the transition to unicells, we shear-shocked tissues by injecting liquid media under pads. Cells detached from tissues and transitioned to motile rods, swimming away from compression zones (Movie S9). Our findings suggest that mechano-responsive multicellularity is an adaptive trait and likely beneficial in compressive zones exceeding lethal thresholds in environments such as desiccated salt plates, animal guts, and microbial biofilms (30).

### Archaeal tissues undergo FtsZ-independent cellularization, resulting in a radial symmetry with distinct cell types

**Fig. 3:**
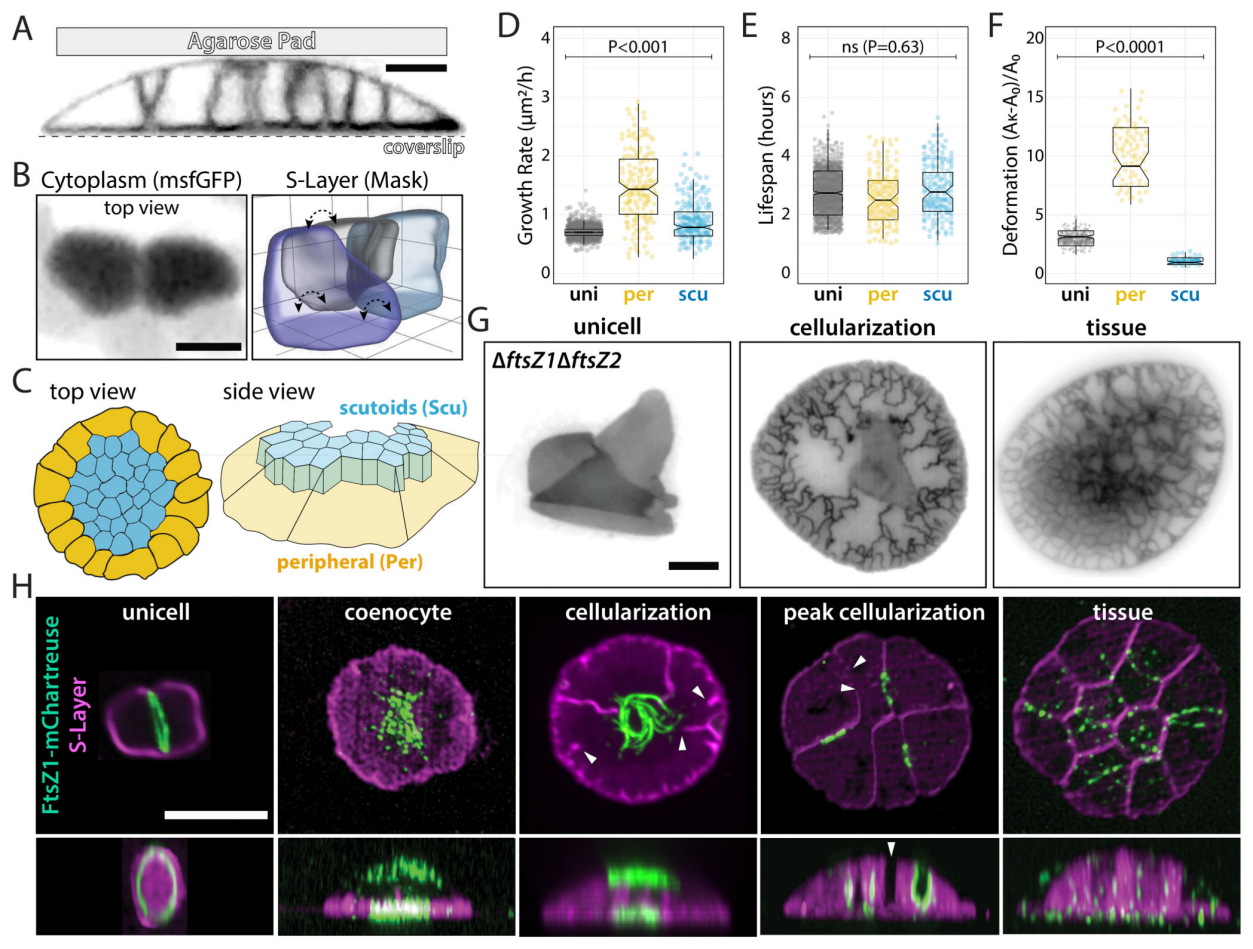
Cellularization is independent of FtsZs and results in two cell types. **(A)** 3D-STED super-resolution microscopy of the cellularization process. **(B)** (Left) iSIM of cells expressing cytoplasmic msfGFP and (right) 3D outline masks of scutoid cells segmented from 3D-STED highlights scutoid cells. Dashed arrows indicate the different scutoid surface neighbors across z planes. **(C)** Cartoon representation of top and side views showing Per and Scu cell types within tissues. **(D-F)** Area growth rate **(D)**, lifespan **(E)** and deformation **(F)** measurements of unicells, Per, and Scu cells from phase-contrast time-lapses. **(G)** Epifluorescence microscopy of representative cell-division impaired Δ*ftsZ1*Δ*ftsZ2* cells across different developmental stages. Early cellularization represents cells that just entered the cellularization stage. Peak cellularization represents cells at the onset of completing cellularization. **(H)** 3D-SoRa microscopy of representative cells expressing FtsZ1-mChartreuse across different developmental stages. White arrowheads indicate septation sites without FtsZ1 signal. Scale bars: 2 μm.

While compression yielded tissues with larger peripheral cells (Fig. 2D), cell size and shape changes could result from uneven distribution of mechanical forces within the device instead of mechanosensation by specific cells. Since specialized cell types are a hallmark of multicellularity (31), we characterized the cellular morphology and cell cycle in different tissue regions. 3D-STED micrographs showed two profiles: wider but shorter cells at the periphery and taller cells at the center of tissues (Fig. 3A, Movie S10). 3D-STED projections showed irregular scutoid-like center cell shapes similar to those stabilizing curved epithelia during embryogenesis (33). From 3D outline segmented masks, we observed variation in cell neighborhoods across the scutoid regions, suggesting a maximization of packing typical of animal scutoids (Fig. 3B). Based on their radial symmetry and position within the tissue, we named tissue cells peripheral (Per) and scutoid (Scu) cells (Fig. 3C).

Moreover, 3D-STED data suggests Per cells are not in contact with the pad surface, indicating that their lower height is not a direct consequence of compression. In contrast, Scu cells are in physical contact with compression areas, suggesting they could directly respond to the mechanical compression from pads. Inspired by traction force microscopy (32), we imaged fluorescent beads embedded in pads, observing an upward displacement of beads over the tissues’ Scu but not Per region (Fig. S8A).

Another feature of animal scutoid cells is minimizing energy by distributing membrane tension. This led us to determine whether Scu and Per cells maintain a constant surface-to-volume ratio. Although Per cells have larger volumes and surface areas, Per and Scu surface:area ratios remained consistent (Fig. S8B). Our data suggest that scutoid-like cells may predate eukaryotes.

Mechanically stressed cells are typically smaller, grow slower, and have shorter lifespans than unstressed cells (34). Comparing the growth rates and lifespans of Per and Scu to those of unicells, both cell types showed higher growth rates (Fig. 3D) and similar lifespans (Fig. 3E) relative to unicells. Our data support the idea that simple physical packing or nutritional stresses cannot summarize Per and Scu cell shape profiles.

To expand on the Per-Scu specialization, we examined whether differences in size and shape could translate to distinct viscoelastic properties. After breaking tissues by mechanical shear, we subjected individual cells to a new compression cycle and measured their deformation. While Per cells were ~3-fold more deformed than unicells, Scu cells were ~2.5 more rigid than unicells (Fig. 3F, Movie S11), supporting the model where stiffer Scu cells counteract compression, so flexible Per cells can escape compression zones.

Specialized cell types in animal tissues often adapt their cytoskeleton to mechanical cues. Although FtsZ1 and FtsZ2 paralogs are required for cell division in unicells (35), Δ*ftsZ1*Δ*ftsZ2* cells were still capable of cellularization under compression with “zig-zag” askew cell junctions (Fig. 3G). This observation parallels animals and holozoans, where microtubules are dispensable (36). Imaging knock-in FtsZ1-mChartreuse cells (37) (Fig. S9A), we observed continuous FtsZ1 rings converting to sparse filaments at the top and bottom of the central coenocytic region (Fig. 3H). Supporting the conclusion that cellularization is FtsZ-independent, FtsZ1 was absent at 42% of the division furrows (Fig. S9B, white arrowheads). Collectively, these observations support a sequential developmental program, with tissues relying on distinct molecular machinery for cell division that is absent in unicells.

### Archaeal tissue cellularization is triggered by envelope tension

Evidence from flies and holozoans suggests that the timing of cellularization correlates with a specific threshold ratio between the number of nuclei per cell volume (N/C) (38). Imaging *Hvo* cells expressing mNeonGreen-PCNA, we quantified the number of replication sites and the fluorescence intensity within replication sites relative to cell area as a proxy for N/C. If N/C is critical for cellularization, DNA replication would increase faster than cell area. However, mNeonGreen-PCNA at replication sites remained constant relative to cell area, indicating that N/C does not influence cellularization (Fig. S10A).

**Fig. 4:**
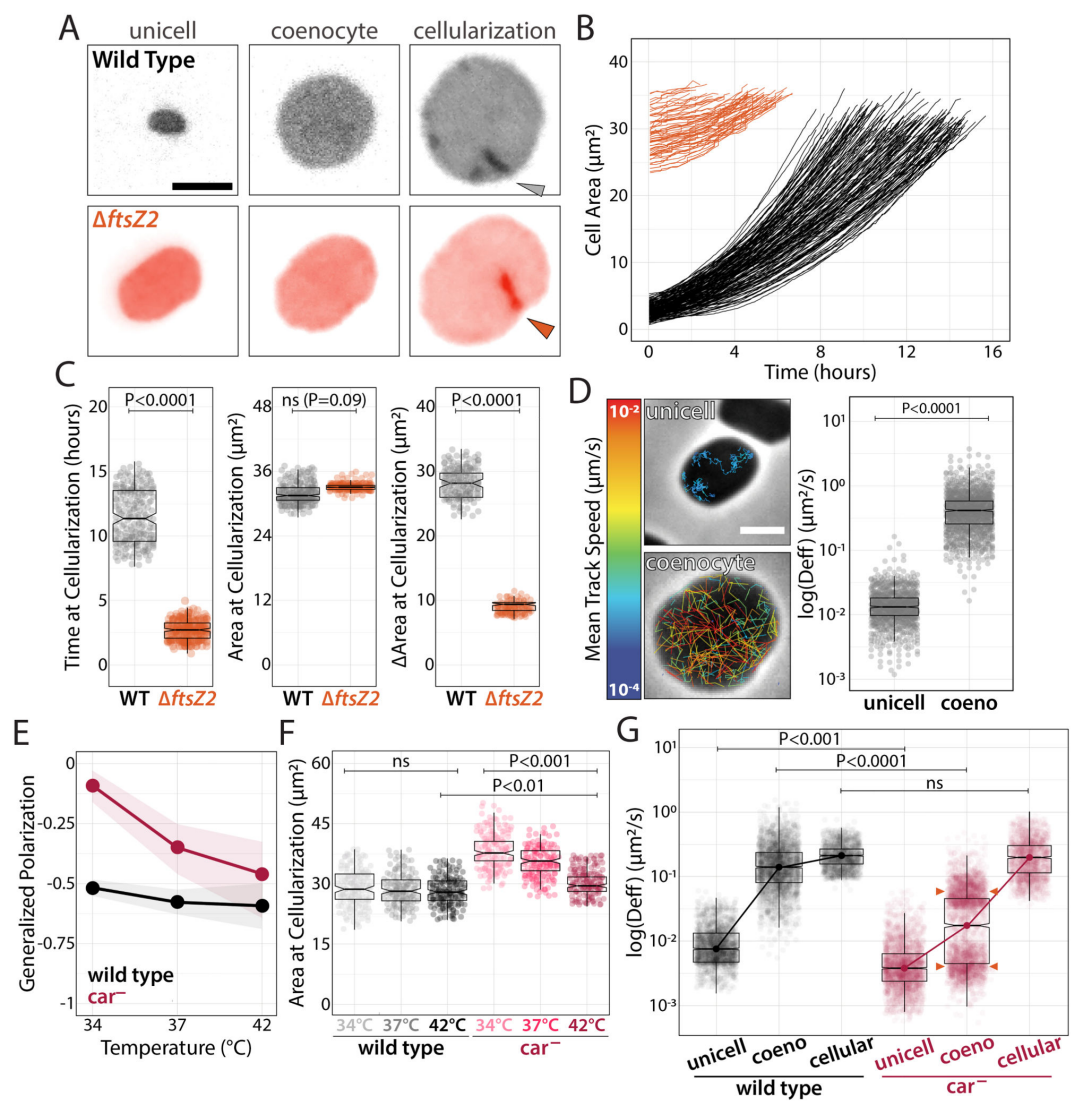
Tissue cellularization is triggered by coenocytic size through a membrane tension threshold. **(A)** Epifluorescence micrographs of wild-type (top) and Δ*ftsZ2* (bottom) cells from compression to cellularization. **(B)** Single-cell growth curves from compression to cellulation onset. **(C)** Time, cell area, and cell area are added at the onset of cellularization. **(D)** Phase-contrast images of unicell and coenocytes and bSpoJ single-molecule tracks overlay false-colored relative to their mean speed. Effective diffusion coefficients are calculated from MSD curves. **(E)** Live-cell Generalized Polarization measurements of wild-type and *car*^−^ stained with Laurdan. **(F)** Area at cellularization measurements of wild-type and *car*^−^ cells from phase-contrast time-lapses across temperatures. **(G)** Wild type and *car*^−^ membrane fluidity calculated by bSpoJ effective diffusion coefficients across developmental stages at 34°C. Correlation between cellularization areas and bSpoJ diffusion in wild-type and *car*^−^ cells at 34°C. Scale bars: 2 μm.

Next, we tracked single-cell parameters such as size, time, and the amount of added cell volume (39) at cellularization (Fig. 4A and 4B), observing a lower coefficient of variance for cell area (CV=9.6%) compared to area added and time (CV=24.2% and 47.1%, respectively) (Fig. 4C). To confirm that cellularization happens at a specific coenocyte size, we compressed larger Δ*ftsZ2* unicells, resulting in cellularization ~4.2 times faster and ~3.7 times less added area compared to wild type, but still at a consistent (Fig. 4C).

Although cell size is set at cellularization, the underlying mechanism remains unclear. Given the differences in membrane tension between tissues and unicells (Fig. 1H), we hypothesized that cell envelopes accumulate elastic strain as coenocytes grow larger until the critical threshold to initiate cellularization. To measure membrane tension in real time, we created bSpoJ, a single-molecule live-cell membrane fluidity reporter. bSpoJ is a chimeric transmembrane domain of SpoIIIJ from *Bacillus subtilis*, with a secretion signal peptide from *Hvo*, and HaloTag. Particle tracking of bSpoJ in *Hvo* showed a ~30-fold increase in apparent diffusion in coenocytes compared to unicells, an order of magnitude above the ~2.5-fold increase in cell area (Fig. 4D and S10B; Movie S12 and S13).

The disproportionate increase in membrane tension relative to cell area suggests biochemical changes in the coenocytic envelope throughout development. Because carotenoids are thought to play a similar role in bacteria as cholesterol in eukaryotes (40), we measured membrane fluidity in cells stained with Laurdan, a fluorescent reporter for generalized polarization (41). Despite the lack of obvious phenotypic defects in *car*^−^ unicells at higher or lower temperatures (Fig. S10C), *car*^−^ showed a weaker membrane fluidity homeostasis than wild type at lower temperatures (Fig. 4E and S10D). Consistently, *car*^−^ showed decreased coenocytic viability (Fig. S10E). Colony pigmentation also appeared attenuated in *Hmed* and tissue-defective strains (Fig. S10F and S11), suggesting a positive correlation between carotenoids and tissue development in opposition to biofilm production.

The loss of *car*^−^ membrane fluidity control implies that coenocytes fail to trigger cellularization below critical tension thresholds. Hence, we expect changes in cellularization cell sizes among survivors. As predicted, we observed increased cell area at cellularization (Fig. 4F), suggesting *car*^−^ can only match membrane tension threshold at larger coenocyte areas. To directly probe membrane fluidity at cellularization, we compared bSpoJ diffusion between wild type and *car*^−^ at 34°C (Fig. 4G). Once again, we observed lower membrane fluidity in *car*^−^ unicells compared to wild type as recorded by generalized polarization. bSpoJ diffusion at tissue cellularization was similar in wild type and *car*^−^, supporting a tension control mechanism. Curiously, we observed a bimodal distribution in *car*^−^ coenocytes (orange arrowheads). We speculate that one sub-population with higher bSpoJ diffusion rates reaches the membrane tension threshold only at a larger cell size. In contrast, the second group stochastically fails to meet the tension-area ratio requirement and undergoes lysis. These results highlight a mechanoresponsive mechanism whereby cells regulate membrane fluidity to secure precise cellularization timing.

### Actin and N-glycosylation are fiducial markers for archaeal tissue polarity

**Fig. 5:**
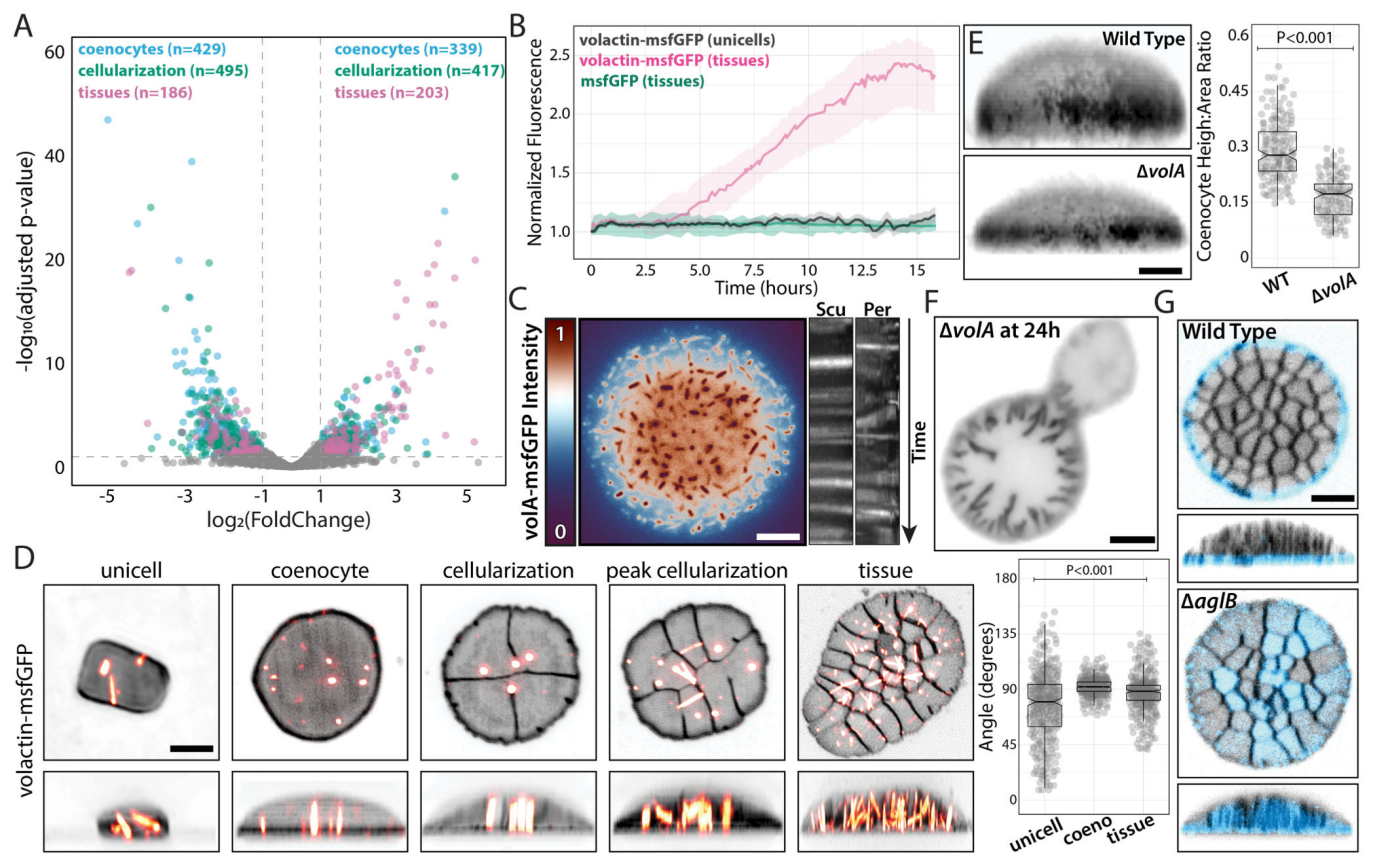
Volactin and N-glycosylation are tissue-specific polarity markers. **(A)** Volcano plot overlays from RNA-seq datasets collected across developmental stages and normalized by liquid unicellular cultures. Parentheses indicate the number of candidates above the arbitrary cutoff. **(B)** Normalized fluorescence by cell area of volA-msfGFP and constitutively expressed cytoplasmic msfGFP from confocal time-lapses. **(C)** Epifluorescence micrograph of a false-colored tissue relative to volA-msfGFP fluorescence (left) and dynamics of volA-msfGFP polymers represented by kymographs (right) from Per and Scu regions. **(D)** 3D-SoRa projections of representative cells expressing volA-msfGFP across developmental stages (left) and volA cable angle measurements relative to the coverslip plane (right). **(E)** Height measurements of wild-type and Δ*volA* coenocytes from 3D-SoRa projections. **(F)** Representative epifluorescence micrograph of a Δ*volA* cell stalled at cellularization. **(G)** 3D-confocal projections of cell surface N-glycosylated proteins in wild-type (top) and Δ*aglB* (bottom) tissues stained by ConA-Alexa488. Scale bars: 2 μm.

To identify multicellular factors, we performed RNA-seq across developmental stages (Fig. 5A). Gene expression profiles revealed that coenocytes repressed twice as many as upregulated genes, possibly involving pathways related to stress response and cell division arrest (Fig. S12A). Although the number of differentially expressed genes (~28% of the genome) is distributed equally across the genome (Fig. S12B), most of the highest-expressed genes in tissues are clustered in the third quarter of the main chromosome between the HVO_2134 and HVO_2150 *loci* (Fig. S12C). This pattern suggests the existence of possible transferable islands related to the emergence of multicellularity. We also observed an enrichment of proteins with cytochrome-related and photosynthetic reaction center (PRC) domains, which were shown to have structural cell division roles (42, 43). Hence, the above paralogs could represent tissue-specific cell cycle factors.

From our gene candidate list, we focused on four groups: cell surface, cytoskeleton/mechanosensing, signaling, and cell cycle (Data S2). Given the importance of actin in eukaryotic tissue biogenesis, we investigated the role of volactin (volA), the only identified actin homolog in *Hvo* (44), which is upregulated ~1.6-fold in tissues. Time-lapses showed increased volA-msfGFP signal during development, reaching a steady state by the end of cellularization (Fig. 5B; Movie S14). In contrast, unicells showed unaltered volA-msfGFP levels, as did tissues expressing cytoplasmic msfGFP. Moreover, volactin polymers were less abundant but highly dynamic in uncompressed Per cells than unicells and Scu scutoids (Fig. 5C; Movie S15). VolA also displayed changes in structural patterns, with cables aligning in coenocytes and Scu cells (Fig. 5D; Movie S16). Compression of Δ*volA* cells resulted in shorter coenocytes than wild type (Fig. 5E) and delayed or stalled tissue maturation (Fig. 5F). Because volA is implicated in regulating the development of rod-shaped (motile) and disk-shaped (sessile) cell types (44, 45), we propose that volactin may mirror eukaryotic actin's multifunctional roles as a mechanosensing cytoskeleton and polarity factor in tissues (46).

Comparative genomics leveraging the differences in tissue formation across *Hfx* species showed substantial overrepresentation of orthologous groups (orthogroups) related to protein glycosylation, sugar metabolism, and transport in presence/absence datasets among (i) *Hvo* and *Hmed* (Fig. S12D; Data S3); (ii) 4 *Hfx* species (Fig. S12E; Data S4); and (iii) orthogroups enriched in species that form tissues (Data S5). Protein glycosylation has historically been studied in haloarchaea for its role in mating and envelope biogenesis (47). In eukaryotes, N-glycosylation is crucial for cell identity, polarity, junctions, and adhesion (48). To explore the role of N-glycosylation, we labeled cells with ConA-Alexa488, a cell-impermeable fluorescent lectin conjugate that binds mannose glycol groups. While ConA-Alexa488 did not stain unicells (Fig. S13A), it exhibited a radial localization at the outline of Per cells (Fig. 5G; Movie S17). To test whether patterns are formed by biofilm accumulation in the extracellular matrix, we examined a Δ*pibD* mutant, blocking all pili-dependent secretion (49). We observed no significant differences in ConA-Alexa488 localization in Δ*pibD* tissues, indicating that biofilm is not critical for cell junctions. Next, we imaged tissues of different N-glycosylation mutants, finding that Δ*aglB* is the only one to disrupt ConA-Alexa488 halos, resulting in staining of the whole tissue surface (Fig. 5G and Fig. S13B). Therefore, we propose that AglB, the main oligosaccharyltransferase required for S-layer N-glycosylation (47), acts as an inhibitor of N-glycosylation in Scu cells, directing tissue cell-surface polarity. This discovery adds a novel function to S-layer glycoproteins beyond morphogenesis and mating.

The discovery of clonal, tissue-like multicellularity in archaea highlights the potential of archaeal mechanobiology to shed light on the emergence of complexity in nature. Nevertheless, this is not the first developmental program identified in haloarchaea, joining the rod-shaped (motile) and disk-shaped (sessile) shape-shift transitions (45). These cell types are connected by volactin, which is also required for disk formation (44). The coordinated alignment of volactin cables indicates that actin cables might sense membrane curvature or mechanically support coenocytic and Scu cells. While these scenarios are not mutually exclusive, volA's roles in different developmental programs, combined with the FtsZ-independent tissue cellularization, underscore a developmental transition from tubulin-dependent to actin-dependent cellularization (25, 46).

An integrative evo-devo mechanobiology approach offers a framework for understanding the link between microbial biofilms and membrane homeostasis, which may regulate the transition between different multicellularity modalities. Our findings also suggest the need to revisit past evidence for the origin of eukaryotic multicellularity, such as fossils identified as holozoans (50). Based on their size and absence of definitive eukaryotic features, these fossils may represent ancestors of archaeal tissues. Future studies should uncover the biochemical and structural nature of archaeal cell junctions and expand the presence of archaeal tissues in phyla evolutionarily closer to eukaryotes, such as the Asgard archaea.

## Supplementary Material

Data S1 - S5

Movies S1 - S17

Supplementary Materials

## Data Availability

All custom software developed in this work is publicly available without restrictions in a Zenodo repository (90). The complete raw RNA-seq datasets presented in this study can be found in NCBI GEO online repository PRJNA1165275. Haloarchaeal strains are available from the Leibniz Institute DSMZ-German Collection of Microorganisms and Cell Cultures GmbH (DSMZ) strain bank.
