# Supplementary material for: Tissue-Like Multicellular Development Triggered by Mechanical Compression in Archaea
Source: Science. Author manuscript; Available in PMC 2025 Jun 6. (PMC7617734; doi:10.1126/science.adu0047)
Supplement: Supplementary Materials [file EMS206035-supplement-Supplementary_Materials.pdf]

Supplementary Materials for

Tissue-Like Multicellular Development Triggered by Mechanical Compression in Archaea

Theopi Rados[1]†, Olivia S. Leland[1]†, Pedro Escudeiro[2], John Mallon[1], Katherine Andre[1], Ido Caspy[3], Andriko von Kügelgen[3] Elad Stolovicki[4], Sinead Nguyen[1], Inés Lucía Patop[1], L. Thiberio Rangel[5], Sebastian Kadener[1], Lars D. Renner[6], Vera Thiel[7], Yoav Soen[4], Tanmay A.M. Bharat[3], Vikram Alva[2], Alex Bisson[1]*

Corresponding author: bisson@brandeis.edu

The PDF file includes:

Materials and Methods
Figs. S1 to S13
Tables S1 to S7
References

Other Supplementary Materials for this manuscript include the following:

Movies S1 to S17
Data S1 to S5

Materials and Methods

Statistical Analysis

The experimental design, sample sizes, number of biological replicates, and statistical tests are summarized in Table S7. In summary, distributions are presented as boxplots, where the box limits indicate the interquartile range around the median, and error bars represent a 95% confidence interval (CI). For XY graphs, shaded areas indicate the 95% CI. When applicable, P-values and statistical significance were evaluated using Kolmogorov-Smirnov (for distributions with at least 50 data points that do not assume normality) or One-Way ANOVA (for comparisons between two groups, with three data points each).

Haloarchaeal Strains

All haloarchaeal species (other than *Hfx. volcanii*, *Hfx. mediterranei*, *Hca. hispanica*, and *Hbt. salinarum*, which were kindly shared by Amy Schmid, Duke University) were provided by the DSMZ culture collection as described in Table S4.

Media and Culture Growth

All *Haloferax* species and strains were grown in the semi-defined Hv-Cab medium (52). Strains were streaked from -80°C freezer glycerol stocks onto Hv-Cab plates (1.5% agar), supplemented with 20 µM tryptophan and 50 µM uracil when necessary, and incubated for 2-3 days at 45°C. Single colonies were transferred into 16×25 mm glass tubes with 3 mL of liquid Hv-Cab, placed on a roller drum, and grown at 42°C to mid-exponential phase (OD_{600nm}~0.5). When necessary, the expression fluorescent reporters were induced by adding 500 µM Tryptophan. Other haloarchaeal species (as described in Table S4) were grown on 372-Cab medium, adapted from Hv-cab for other halophilic organisms: 3.3M NaCl, 56mM KCl, 180mM MgCl2, 174mM MgSO4, 10g/L Casaminoacids (Oxoid), pH 7.4, supplemented with thiamine 1 g/L, biotin 0.1 g/L, and trace elements (4.9mM $FeCl_3$, 0.37mM $ZnCl_2$, 0.074 mM $CuCl_2$, 0.077mM $CoCl_2$, 0.16mM $H3BO_3$, 12.7mM $MnCl_2$, 0.065mM $NiSO_4$, 0.041mM $Na_2MoO_4.2H_2O$).

Growth Curves

Hfx. volcanii strains were grown at 42°C until an OD_{600nm}=0.5, diluted 10-fold to an OD_{600nm}=0.05 and transferred to a 96-well flat-bottom plate (Corning Inc., #3370) next to Hv-Cab blanks. All wells surrounding the samples were filled with 200 µL ddH_2O to prevent media evaporation. Growth curves of biological triplicates were performed using an EPOCH2 microplate spectrophotometer (Agilent) with constant orbital shaking at 42°C. OD_{600nm} measurements were collected every 30 minutes for 48 hours. Each measurement was averaged across triplicates and then subtracted from the averaged Hv-Cab blank measurements.

Storage Modulus Measurements by Dynamic Mechanical Analysis (DMA)

Fresh pads under different agarose concentrations (0.25, 0.5, 1.0, 1.5, 2.0, 2.5, and 3.5%, w/v) were sent to Element Materials Technology (New Berlin WI) for Dynamic Mechanical Analysis (DMA). A small circle was cut from the provided agarose pad and placed within a Thermal Analysis (TA) DMA Q800 instrument equipped with a compression clamp fixture. The Storage Moduli (E') were calculated using the equation (53):

$$E' = \frac{\sigma_0}{\gamma_0} \cos \delta$$

where σ_0 is stress, γ_0 is strain, and δ is the phase lag between stress and strain. The analysis was conducted at 1 Hz and an oscillating amplitude of 3 µm. Each sample was heated from 10°C to 80°C at a 2°C/min rate.

Membrane Fluidity Measurements with Laurdan

Cells were grown as described above and kept at the desired temperature (34°C, 37°C, or 42°C) during the experiment unless indicated. To increase Laurdan permeability to *Hfx. volcanii* membranes, cells were spheroplasted by spinning down 1.5 mL of culture (2,500xg for 2 minutes) and resuspending the pellet in 100 µL of EBS Solution (1M NaCl, 25mM KCl, 0.4M Sucrose, 50mM EDTA, and 50mM Tris-HCl pH 8.5). After a 10-minute incubation at room temperature, 10% DMF and 100 µM Laurdan (Invitrogen, #D250) were added to spheroplasts, and tubes were mixed at maximum speed in a ThermoMixer Compact (Eppendorf) at desired temperatures (34°C, 37°C, or 42°C) for 10 minutes. A 2 µL droplet of spheroplasted culture was transferred to a prewarmed 35mm glass-bottom dish (Ibidi, #81218-200) and gently covered with a pre-warmed 0.25% agarose pad prepared for S-layer staining as described above. Cells were then imaged on a Nikon Ti2E inverted microscope AX-R resonant scanning confocal with 405, 488, 561, and 640 nm lasers, aa CFI60x Apochromat oil objective and OKOlab caged incubator set to the desired temperature. Samples were imaged using the 640 nm laser (for S-layer) and the Spectral Detection for capture every 10nm from 420 nm to 670 nm (for Laurdan). For analysis, a binary mask was created for the S-layer image using the built-in Thresholding (Default setting). The mask was then applied to the individual Spectral Detection images after background subtraction, and the mean gray value was obtained for each cell.

Hvo and *Hmed* Viability under Micropillar Chambers

Mid-exponential *Hfx. volcanii* and *Hfx. mediterranei* liquid cultures (OD_{600nm}=0.5) were diluted 100-fold and 2 µl were transferred to 50 mm MatTek dishes (1.5mm coverslips) and compressed under microchambers fabricated as described in the "Micropillar Chambers" section. After confirming that cells were sparsely distributed (no more than one cell for every 10x10 µm pillar) by phase-contrast microscopy, samples were incubated at 42°C for 24 hours. Following incubation, cells were washed out from chamber with Hv-Cab liquid media, OD_{600nm} adjusted to 0.1, and 100 µl from serial dilutions were plated onto Hv-Cab agar plates. After 48 hours, colonies were counted and colony unit formation (CFU) determined. Viability was plotted as values normalized by CFU from mid-exponential liquid cultures used as starting inoculum. For the viability comparison between *Hvo* colonies and tissues (Fig. S7D), 10 individual colonies were picked from Hv-Cab agar plate streaks, resuspended in liquid Hv-Cab, serial dilutions plated and CFU determined as described above.

Quantification of Biofilm Mass from *Haloferax* species

Biofilm was quantified with crystal violet as described in Schulze et al. (54) with modifications. Briefly, biological triplicates of *Haloferax* species were grown in Hv-cab at 42°C to an OD_{600}~2.0,

under constant agitation in a roller drum. Cultures were then diluted to an OD_{600} of 1.5 and incubated at 42°C static for 60h. Cells were pelleted by centrifugation at 3000xg for 10 minutes and resuspended in 30 µL of Hv-cab (1 µL was used for serial dilutions, plated for CFU count). 200 µL of 2% acetic acid was added to the remainder of the cell suspension, which was then incubated at RT for 15 minutes, followed by centrifugation at 10,000xg for 2min. 200 µL of 0.1% crystal violet was added to the pellet, followed by vortexing to break the pellet, incubation at RT for 20min, and centrifugation at 10,000xg for 2min and two ddH2O washes. The pellets were dried for 20 minutes, followed by adding 500 µL of 30% acetic acid, and incubated at RT overnight, protected from light. 20 µL of the sample was then transferred to a 96-well plate, mixed with 180 µL of 30% acetic acid per well, and absorbance was measured using an EPOCH2 microplate spectrophotometer (Agilent) at 550nm. Absorbance values were normalized by colony formation units (CFU).

Quantification of Carotenoid Levels from *Hfx. volcanii and Hfx. mediterranei*

Carotenoid quantification was done as described in Cerletti et al. (55) with minor modifications. Briefly, cells were grown to stationary (OD_{600} 1.5), centrifuged for 10 minutes at 4500xg and the pellet was resuspended in 1 mL of 1:1 Methanol:Chloroform and vortexed thoroughly for 10min. Samples were then centrifuged for 2min at 10000xg, and the 495nm and 475nm absorbance peaks of the supernatant were measured using a UV-Vis Spectrophotometer (Nanodrop, ThermoScientific) and used to calculate carotenoid concentration.

RNA-seq of Multicellular Developmental Stages

DS2 cells were grown to an OD_{600} of 0.15 in Hv-Cab and concentrated by centrifugation to an OD_{600} of 0.6 in the same media. An aliquot of this cell culture was then saved for RNA extraction and corresponds to time 0h. Thirteen 3 µL droplets of cell culture were then placed equidistantly on glass Petri dishes (Corning 3160-101BO), and each was covered with a 1.5cm x 1.5 cm pad. The plates were washed with warm (42°C) Hv-cab for cell retrieval after 6h, 12h and 24h. Cells were immediately concentrated by centrifugation (4500xg for 10 minutes) and lysed with 1 mL of TriZol (Invitrogen) per sample. Total RNA extraction was performed as described (Rados and Andre, 2023). Samples were sent to SeqCenter LLC (Pittsburgh, USA, seqcenter.com) for ribosome depletion using *Haloferax*-specific probes (56) and sequencing. Pre-processing and quality filter of paired-end raw sequencing reads were performed by fastp (v.0.23.4) (57) under default arguments. Reads that passed this quality filtering step were eligible for downstream transcript quantification using Salmon (v.1.10.0) (58) in a two-step procedure: 1) The nucleotide CDS sequences for the *Haloferax volcanii* genome ("_cds_from_genomic.fna.gz" file) were downloaded from the NCBI Genomes FTP website (59), using the respective assembly and RefSeq accessions (Table S1). To build an index of the *Haloferax volcanii* transcriptome, the "salmon index" command was run using the file above as input and default arguments. 2) Transcripts were quantified with the "salmon quant" command, using the quality-filtered reads and the index built in the previous step as input. The sequence-specific bias correction parameter was enabled, and the library type was set to be automatically inferred. All other parameters were left to default. A gene count matrix with dimensions "number of samples" versus "number of genes" was created with the read counts generated by Salmon. Genes with less than 10 reads across all samples were discarded from this matrix.

Differential Gene Expression (DGE) analysis

DGE analysis was performed with PyDESeq2 (v.0.4.9) (60), using the gene count matrix as input and metadata concerning the experimental design. Gene expressions were compared between the reference (unicells, T=0h) and the samples collected at the 6h (coenocytes), 12h (cellularization), and 24h (tissues) time points. Genes whose |log2(Fold Change)| ≥ 1 and adjusted p-value ≤ 0.05 were used for downstream data analysis and visualization. Data analysis was carried out with Pandas v.1.5.3 (61), and plots were created using Matplotlib v.3.7.1 (62). These packages were installed and run under Python v.3.10.12.

Tree of *Haloferax* Species

Annotated protein sequences from Halobacteria genomes were retrieved from the NCBI GenBank database, out of the 315 genomes 78 are part of the *Haloferax* genus. The accession numbers and associated metadata for these genomes are provided in Supplementary Data S5 and Data S6.

Homologous gene clusters were identified using GET_HOMOLOGS's (63) implementation of the OrthoMCL algorithm (64). A total of 385 core homologous groups are present in at least 90% of the studied Halobacteria genomes. Each homologous group was independently aligned using MAFFT (65) with --reorder and --auto parameters. Once aligned, sequences were concatenated into a single supermatrix comprising 162,041 sites.

A phylogenetic tree was reconstructed using IQ-TREE (66), and the substitution model LG+F+I+G4. Bipartition support was estimated using both the Ultrafast Bootstrap (i.e., UFBoot) with 1,000 replicates (67) and the SH-like approximate likelihood-ratio test (SH-aLRT) with 1,000 replicates (68). The unrooted maximum likelihood tree generated by IQ-TREE was rooted using the Minimal Ancestor Deviation (MAD) method (69) to objectively determine the root position within Halobacteria without the need for an outgroup. MAD version 2.0 was employed with default settings, minimizing the deviation in root-to-tip lengths across the tree.

Comparative Genomics Analysis of *Haloferax* Species

The protein sequences ("_protein.faa.gz" suffix file) for each of the 11 *Haloferax* species' genomes shown in Table S1 were downloaded from the NCBI Genomes FTP website (70), using the assembly and RefSeq accessions respective to each species. OrthoFinder (v.2.5.5) (71) was run using the protein sequences from these 11 genomes as input, with default arguments. OrthoFinder was also run separately for two other datasets: (i) one containing the protein sequences from the *Haloferax volcanii*, *Haloferax gibbonsii*, *Haloferax prahovense*, and *Haloferax mediterranei* genomes, and (ii) another containing the protein sequences from the *Haloferax volcanii*, and *Haloferax mediterranei* genomes. Genomic features for the *Haloferax volcanii* protein sequences within each set of orthologous sequences (orthogroup) were gathered from the "_feature_table.txt.gz" file for the respective genome (Table S1). This file was downloaded from the NCBI Genomes FTP website. The "idmapping.dat" file was downloaded from the UniProt FTP website (72). Next, the RefSeq id of each protein sequence was cross-referenced against it to retrieve the respective UniProt id. Data analysis was carried out on the output files generated by OrthoFinder using Pandas v.1.5.3 (61), installed and run under Python v.3.10.12.

Over-Representation Analysis (ORA) of Orthologous Protein Groups

The protein counts generated by OrthoFinder were used for all orthogroups and each of the 11 Haloferax species in Table S1. Our dataset of 11 species was divided into two groups: (i) species that form tissue and (ii) species that do not form tissue (Table S1). The null hypothesis H0 was defined as orthogroup membership and belonging to the group of species that form tissue are independent properties, and the alternative hypothesis H1 as there is an over-representation of proteins from an orthogroup that belong to the group of Haloferax species that form tissue. This problem was formulated as a contingency table (Table S2). The test performed was the one-sided hypergeometric test, and the critical region chosen was the upper tail of the distribution. To calculate the p-value associated with this test and for each orthogroup, the survival function of the hypergeometric distribution was used. To control the false positive rate (FDR) from these multiple comparisons, the Benjamini-Hochberg correction (73) was applied to all p-values calculated this way. The over-represented orthogroups that satisfied all the following conditions were considered for downstream data analysis and interpretation: (i) the orthogroup has at least one protein from the Haloferax volcanii genome; (ii) the orthogroup has proteins from at least 4 *Haloferax* species that form tissue; (iii) the orthogroup has a hypergeometric test FDR-adjusted p-value ≤ 0.05. Data analysis was carried out with Pandas v.1.5.3 (61), hypergeometric tests were performed with SciPy v.1.14.0 (74), and the Benjamini-Hochberg correction to the p-values was achieved with statsmodels v.0.14.2 (75). These packages were installed and run under Python v.3.10.12.

Cryo-EM and cryo-ET of cells from sheared tissues

Grids for cryo-EM experiments were prepared as described previously (76). Briefly, 2.5 µL of *Hfx. volcanii* unicells were supplemented 10:1 (cells:gold solution, v/v) with 10 nm protein-A gold (CMC Utrecht) immediately prior to grid preparation. For sheared multicells, *Hfx. volcanii* cells were grown under compression for 16 to 17 hours, then washed with the Hv-cab media supplemented 8:1 (cells:gold solution, v/v) with 10 nm protein-A gold (CMC Utrecht). These mixtures were then applied to a freshly glow discharged Quantifoil R2/2 (washed multicells) or R3.5/1 (unicells) Cu/Rh 200 mesh grid, adsorbed for 10 s, blotted for 4-5 s and plunge-frozen into liquid ethane that was maintained at -178 ºC in a Vitrobot Mark IV (ThermoFisher), while the blotting chamber was maintained at 100% humidity at 10 ºC. The frozen grids were stored in liquid nitrogen until further investigation.

Cryo-ET data was collected as described previously (77-79). Briefly, for tomographic data collection, the SerialEM software (80) was used in a Titan Krios microscope equipped with a Quantum energy filter (slit width 20 eV) and K3 direct electron detector running in counting mode. Tilt series with a defocus range of −3 to −6 µm (washed multicells) or -5 to -8 µm (unicells) were collected between ±60° in a dose symmetric scheme (81) with a 3° (washed multicells) or 2° (unicells) tilt increment. A total dose of 80 $e^-/Å^2$ (washed multicells) or 85 $e^-/Å^2$ (unicells) was applied over the entire series. Cryo-ET data analysis: tilt-series alignment and tomographic reconstruction were carried out using IMOD (82). Datasets were motion-corrected and dose weighted with MotionCor2 implemented in Relion 5.0 (83). Contrast transfer functions (CTFs) of the resulting motion-corrected micrographs were estimated using CTFFIND4 (84). Data visualization was performed in IMOD.

Live-Cell Microscopy and Image Analysis

Immobilization and Compression of *Hfx. volcanii*: Agarose pads were prepared by mixing Hv-Cab and Seakem agarose (Seakem Lonza Inc 50002) and autoclaving bottles in a pressure cooker (Instant Pot Duo 8-quart V5) for 30 minutes. Homogenous agarose solutions were immediately poured into 100x15mm Petri dishes (Corning™ FisherSci 07-202-030) as a mold and left to polymerize at room temperature overnight. Plates were wrapped in parafilm and stored at 4°C inside Ziploc bags. For tissue development, colonies from fresh plates (under one week old) were grown in liquid until OD_{600nm}~0.2. 3 µL culture droplets were placed onto clean 50 mm Mattek dishes (MatTek Corp., #P50G-1.5-30-F), gently covered with a (unless otherwise indicated) 1.5cm x 1.5 cm 2.5% agarose pad and incubated at temperatures and times indicated in each experiment.

S-layer and ConA-Alexa488 Staining: S-layer was stained with Brilliant Blue FCF, a nontoxic triarylmethane dye impermeable to cells (85) that binds preferentially to glycoproteins. Brilliant Blue FCF was added at a 1:4000 concentration to liquid cultures (Brilliant Blue FCF, Sigma, 3844-45-9) or directly to pads (Brilliant Blue dye, Spice Supreme, NJ) after autoclaving. For ConA-Alexa488 staining, 30 µL of ConA-Alexa 488 1 mg/mL conjugate stock (Invitrogen, C11252) was added to the top of the pad and allowed to diffuse for 8 hours before imaging.

Coverslip Cleaning for Super-Resolution and Single-Molecule Imaging: Coverslips were placed in a custom 3D-printed tray, sonicated for 15 minutes in 2M KOH, and rinsed with MilliQ water. They were sonicated again for 15 minutes in 4% Extran MA 02 detergent (Millipore Sigma, #1075532500) and rinsed with Milli-Q water. They were then sonicated a third time for 15 minutes in 190-proof, benzene-free ethanol, rinsed with ethanol, and parafilm-wrapped and stored in fresh ethanol. Before imaging, coverslips were fished from the container with clean tweezers, sonicated for 5 minutes in ethanol, and dried with a compressed air duster.

Image Segmentation: For time-lapse experiments requiring lineage tracking (ArcCell device, cellularization control, PCNA foci counting, etc), images were segmented using Omnipose (86) with a custom in-house trained model. Segmented images were converted to binary masks, and CellProfiler (87) was used to measure and track cells over time. Unicells with areas above 10 μm^2 or circularity below 0.7 were filtered out for quality control.

Epifluorescence and Phase-Contrast Microscopy: Cells were imaged using a Nikon TiE2 inverted microscope equipped with a Lumencor Sola II Fluorescent LED (380-760 nm), Hamamatsu ORCA Flash 4.0 v3 sCMOS Camera (6.5 µm/pixel), and a CFI PlanApo Lambda 100x DM Ph3 oil objective (NA=1.45). The microscope body was enclosed by an OkoLab Caged incubator set to temperatures indicated in each experiment.

SoRa Microscopy: Cells were grown as described, and a 3 µL droplet was placed on a 35mm glass-bottom dish (Ibidi, #81218-200) and gently covered with a pre-warmed 2.5% agarose pad prepared for S-layer staining as described above. Samples were then incubated at 42°C for 6h (coenocytes), 10-12h (cellularization), or 18-20h (tissues). Super-resolution images were collected

at room temperature using a Nikon Ti-2 equipped with a Yokogawa CSU-W1 SoRa spinning disk, with lasers 640 nm (Brilliant Blue) and 488 nm (GFP), a Plan Apo λ 100x objective (NA=1.45), and a Prime BSI express camera (6.5 µm/pixel), with a Z-step of 0.1µm. Images were processed using NIS-Elements software (Nikon), using NIS.ai for Richardson-Lucy 3D deconvolution.

iSIM microscopy: Cells expressing cytoplasmic GFP were grown as described, and a 3 µL droplet was placed on a 35mm glass-bottom dish (Ibidi, #81218-200) and gently covered with a pre-warmed 2.5% agarose pad prepared. Samples were then incubated at 42°C for 18h. Images were collected at room temperature using a Leica microscope equipped with an iSIM 488 Quad, a Plan Apo λ 100x objective (NA=1.45) with a Z-step of 0.2µm. Images were processed using NISElements software (Nikon), using NIS.ai for Richardson-Lucy 3D deconvolution.

STED Microscopy: *Haloferax volcanii* WT cells (DS2) were grown as described. A 3 µL droplet was placed on a 35mm glass-bottom dish (Ibidi, #81218-200) and gently covered with a pre-warmed 2.5% agarose pad prepared for S-layer staining as described above. Samples were then incubated at 42°C for 14-16h. Super-resolution images were captured at room temperature on an Abberior 3D STED system, mounted on an Olympus IX83 microscope with 405, 485, 561, and 640 nm excitation lasers and a 60x oil immersion objective. Images were segmented using a custom in-house pre-trained Cellpose (88-89) model available at https://zenodo.org/records/14568734 (90). Volume and surface area were calculated using Fiji. Segmentation outlines were processed and rendered using Imaris 10.2 (Oxford Instruments).

Traction Force Microscopy (TFM): Fluorescent polystyrene beads of 50 nm diameter (Thermo Scientific, #09-980-410) were diluted 1:100 in a 0.0125% Tween 20 (Thermo Fisher #28320) solution and vortexed. Petri dishes (Genessee, 32-105G) were plasma treated for two minutes. A 200 µL aliquot of the bead solution was pipetted directly on the Petri dish and allowed to bond for 15 seconds. The Petri dish was inverted to pour off excess solution and then allowed to air dry. *Hfx. volcanii* cells were grown to an $O.D._{600nm}$ of 0.14-0.20, and 1 µL of media was pipetted beneath a bead-embedded 2.5% agarose pad, bead side down. 3D-stack timelapses were collected at 1-hour intervals by both phase-contrast and epifluorescence. 3D-stacks were collected with 34 slices at 0.2 µm intervals.

Recompression of Cells from Fragmented Tissues: Hvo tissues under 2.5% agarose pads were placed under the microscope and imaged by phase-contrast microscopy. With ongoing timelapses, tissues were broken by shear-pipetting of 50 µl of Hv-Cab media in between pads and coverslips. After having cells ruptured from tissues, cells were recompressed by removal of excess liquid from pad using a kimwipe paper tissue touching the top of the pad around the imaged area. Cells were segmented and tracked as described above, and deformation was calculated as the difference recompressed cell area (A_k) and initial uncompressed cell area (A_0), normalized by the initial cell area (A_0). Annotation of peribasal and apicobasal cells was done manually across tissues.

Volactin Filament Angle Analysis: aBL126 cells were grown as described, and a 3 µL droplet was placed on a 35mm glass-bottom dish (Ibidi, #81218-200). For tissue analysis, cells were gently covered with a pre-warmed 2.5% agarose pad prepared for S-layer staining, as

described above. For single-cell analysis, the process was repeated with a 0.5% agarose pad. Samples were then immediately imaged (single cells) or incubated at 42ºC for 6h (coenocytes), 10-12h (cellularization), or 18-20h (mature tissues). Images were collected at room temperature using a Nikon Ti-2 equipped with a Yokogawa CSU-W1 SoRa spinning disk, with lasers 640 nm (Brilliant Blue) and 488 nm (GFP), a Plan Apo λ 100x objective, and a Prime BSI express camera (6.5 µm/pixel), with a Z-step of 0.1µm. Filament angles were calculated manually using Fiji straight-line tool.

Laser Ablation: Cells were imaged at room temperature using a Nikon Ti-2 equipped with a Yokogawa CSU-W1 spinning disk, with lasers 640 nm (Brilliant Blue), with a Plan Apo λ 100x objective, Prime BSI express camera (6.5 µm/pixel). Different ROIs were selected, and 100mslong ablation pulses were performed using a 405 nm laser. Cells were then subsequently imaged under 30-second intervals for 15 minutes (large area ROIs) or 3-second intervals for 15 seconds (small area ROIs). Mean Squared Displacement (MSD) was calculated by processing timelapse images segmented with a pre-trained Cellpose model (88-89). The resulting image stacks were analyzed using the Fiji plugin TrackMate (91), with the built-in Label Image Detector and Simple LAP Tracker. The resultant masks were exported as ImageJ ROIs to determine the center of mass (COM) for each independent cell. The initial ablation coordinates, along with the COM coordinates from the first and last frames of each cell, were then used to calculate the MSD for each cell. For plotting and analysis, only cells that were moving directionally (α>1) and towards the ablation point (Δdistance<1) were considered. The custom Python script for MSD calculations and the pre-trained Cellpose models used can be found at https://zenodo.org/records/14568734 (90). This approach was modified from a thread in the imageJ.sc forum (92).

Microfluidics: The ArcCell chips are produced using standard photolithographic techniques (93). The silicon wafer was coated by a negative tone photoresist (SU-8). After soft backing, the desired pattern was exposed on the wafer via direct writing. The devices have channels with distinguished lengths, widths, and heights (Fig. S1A). After the master was ready, it was cast with polydimethylsiloxane (PDMS) degas and cured at 70°C. When cured, the PDMS was cut, peeled and punched to connect ports to the external tubing. The ready mold was then bonded irreversibly to cover glass using oxygen plasma. The ArcCell device was designed to ensure high cell loadings in the chamber and low shear prefusion of fresh media to cell trapping areas. The device has two working modes: loading and running. The loading output is open, the run output is blocked, and cells and media are gently forced to flow into the trapping chambers. After loading, the run output is open, and the media flow rate at the chamber is reduced. Loading and flow rates in the ArcCell device were performed using the LU-FEZ-0345 FlowEZ Microfluidic Flow Controller (Fluigent) coupled with a 0-1 mL/min Flow Unit L (Fluigent). Cells were loaded under 1 µL/min flow rates, and time-lapses were recorded under a constant flow rate of 2 µL/min.

Micropillar Chambers: The squared microchambers with micropillars were designed using the open software tool KLayout with planar dimensions of $10\times10\mu m^2$. The microfabrication process was previously described in (94-96). Briefly, 3” silicon dioxide wafers (Siegert Wafer GmbH, Germany) were extensively cleaned in a solution of 1:1:5 H_2O_2:NH_4OH:H_2O at 70°C for 10 min before use. After drying, the clean wafers were incubated with a 1:10

hexamethyldisiloxane:isopropanol priming solution directly on top. The priming solution was then removed by spincoating and the wafer was heated on a hot plate to 95°C for 5min on a heating plate to remove excess solvent. The hexamethyldisiloxane primer is activated at 95°C for a further 5min to improve adhesion of the positive photoresist Shipley 1813 (Micro Resist Technology GmbH, Germany). The photoresist is spincoated on the silicon substrate to achieve a height of 1.3 µm according to the manufacturer instructions. After curing, the squared microchamber design was written directly onto the photoresist using a tabletop µMLA system (Heidelberg Instruments, Germany). After exposure, the photoresist was developed in MF-321 (Micro Resist Technology GmbH, Germany) for ~2 min with gentle shaking and dried under a stream of nitrogen. The resulting master template was silanized overnight under vacuum with a drop of heptadecafluoro1,1,2,2-tetrahydrodecyl trichlorosilane (abcr GmbH, Germany). The master design was then replicated into PDMS (Sylgard184) using a ratio of 10:1 base:curing agent, degassed and cured overnight at 65°C under vacuum. Subsequently, the PDMS mold was then separated from the silicon master and replicated in a growth medium-agarose mixture for growth experiments.

***Hvo* and *Hmed* Viability under Micropillar Chambers:** Mid-exponential *Hfx. volcanii* and *Hfx. mediterranei* liquid cultures (OD_{600nm}=0.5) were diluted 100-fold and 2 µl were transferred to 50 mm MatTek dishes (1.5mm coverslips) and compressed under microchambers fabricated as described above. After confirming that cells were sparsely distributed (no more than one cell for every 100x100 µm area) by phase-contrast microscopy, samples were incubated at 42°C for 24 hours. Following incubation, cells were washed out from chamber with Hv-Cab liquid media, OD_{600nm} adjusted to 0.1, and 100 µl from serial dilutions were plated onto Hv-Cab agar plates. After 48 hours, colonies were counted and colony unit formation (CFU) determined. Viability was plotted as values normalized by CFU from mid-exponential liquid cultures used as starting inoculum. For the viability comparison between *Hvo* colonies and tissues, 10 individual colonies were picked from Hv-Cab agar plate streaks, resuspended in liquid Hv-Cab, serial dilutions plated and CFU determined as described above.

Growth Rate and Lifespan Measurements: Epifluorescence and phase-contrast time-lapses (20-minute intervals) of *Hvo* S-Layer-stained unicells were 3D- and 2D-segmented using CellPose2 (88) model available at https://zenodo.org/records/14568734 (90). Segmented images were converted to binary masks, and CellProfiler (87) was used to create lineage tracks to extract single-cell volume and surface area over time. Each lineage was checked manually and traced back to its progenitor cells. The lifespan of each cell was defined as the time between its origin and its subsequent division. Cells that did not complete their entire lifespan within the timelapse were excluded from the analysis. The growth rate was then calculated by dividing the area added by lifespan.

bSpoJ Particle Tracking: bSPoJ expression was induced under 200uM tryptophan and grown to exponential phase ($O.D._{600nm}$~0.2). For bSpoJ tracking in unicells, cultures were incubated with 75-150pM HaloTag-ligand JFx549 (kindly provided by Luke Lavis and lab at Janelia) for 30 minutes under standard growth conditions. The cultures were concentrated 10-fold (3,000 xg for 3 min), and 2 µL droplets of culture were placed on 60×24 mm sonicated-clean coverslips and gently immobilized under pre-warmed 1.5x0.5 cm, round 0.5% agarose pad. For

bSpoJ tracking in early and cellularizing coenocytes, cells from liquid exponential cultures were compressed under 2.5% agarose pads prepared with 200 µM tryptophan and 50-500pM JFx549, placed onto a clean 50 mm Mattek dish (MatTek Corp., #P50G-1.5-30-F) and incubated at 42°C for ~6 (early coenocytic phase) and ~12 (onset of cellularization) hours protected from light. TIRF time-lapses were collected in a Nikon TiE2 inverted microscope equipped with a Hamamatsu ORCA Flash 4.0 v3 sCMOS Camera (6.5 µm/pixel), a CFI PlanApo Lambda 100x DM Ph3 oil objective. The microscope body was enclosed by an OkoLab Caged incubator set at 42°C. TIRF time-lapses were collected under 500ms (unicells) or 50ms (coenocytes) exposure times and 2% laser power from a 70 mW Nikon LUN-F 561 nm laser line.

bSpoJ Particle-Tracking Analysis: Phase-contrast snapshots were automatedly segmented using Omnipose (86) using previously custom-trained model (44-45). Cell masks were exported from Omnipose and merged with the fluorescence TIRF channel. Foci positions were tracked, and diffusion speed was calculated in Fiji using the TrackMate plugin (91). Spot detection was performed with the LoG detector and the following parameters: Object Diameter=0.45 µm, Quality Threshold=0.2, Pre-process with median filter checked, and sub-pixel localization checked. Spots were filtered based on minimum intensity to exclude spots outside of cells. Tracking was performed with the Simple LAP Tracker and the following parameters: Linking Max Distance=1 µm, Gap-closing max distance=0 µm, Gap-closing max frame gap=0. Spot files, containing position information over time, and Track files, containing mean speeds of tracks (µm/s), were exported from TrackMate. Next, tracks from TrackMate were used to calculate Mean Squared Displacements (MSD). First, tracks with a lifetime below 5 frames were filtered out to preserve temporal information. The time-averaged MSD for a given time lag was calculated for each individual track following the equation:

$$\langle MSD(\tau)\rangle = \frac{1}{N}\sum_{t=t_{\text{start}}}^{T-\tau}\left[(x(t+\tau)-x(t))^2+(y(t+\tau)-y(t))^2\right]$$

where x and y are the positions in 2 dimensions (µm), τ denotes the time lag interval between each position, t denotes the current time point of the track, t_{start} is the first-time interval of the track, T is the total time in the track, and N-1 is the number of positions used in the current MSD calculation. For an individual track, the MSD was calculated with increasing time lags starting with the frame rate at which the movie was collected and ending with the total lifetime of the track. The effective diffusion coefficient, D_{eff}, was subsequently extracted from the linear fit to the truncated TA-MSD containing the first 5-time lags, following the equation:

$$MSD(\tau) = 4D_{eff}\tau$$

Tracks with fits $R^2<0.9$ were discarded. All calculations were done using a custom Python script (90).

<u>Cloning and Transformation</u>

Oligos, plasmids, and strains used in this study can be found in Tables S3-S5. Plasmids were cloned using Gibson assembly (97) and transformed into competent *E. coli* DH5α cells, transformants

selected on LB plates supplemented with 100 µg/mL Carbenicillin. Plasmids were transformed into *Hfx. volcanii* using the method previously described (98). Unless noted, *Hfx. volcanii* strains were built from the parental H26 (Δ*pyrE2*) or DS2 wild-type strains. A detailed description of each strain created in this work follows:

aSB14 [pcna::mevR-HaloTag-mNeonGreen-PCNA] was created by transforming the DS2 strain with the eSB14 plasmid, cloned from a Gibson assembly consisting of four fragments: 1) pcna upstream region: PCR with primers oSB33 and oSB42 from DS2 genomic DNA template; 2) mevR-Ppcna: synthetic fragment carrying the mevR resistance cassette and 500bp sequence from the pcna promoter region; 3) HaloTag-15aa-mNeonGreen-pcna: synthetic fragment carrying both HaloTag and mNeonGreen tags and the first 750bp of the pcna gene; 4) pTA131 plasmid linearized with NotI. DS2 transformants were selected on Hv-Cab plates supplemented with 10 µg/ml mevinolin and uracil.

aBL582 [ΔpyrE2 ftsZ1::ftsZ1-mChartreuse] was created by transforming H26 with the eBL416 suicide plasmid, cloned from a Gibson assembly consisting of 4 fragments: 1) ftsZ1 fragment: PCR with primers oJM193 and oAB130 from DS2 genomic DNA template; 2) 40aamChartreuse synthetic fragment; 3) ftsZ1 downstream fragment: PCR with primers oBL532 and oBL533 from DS2 genomic DNA template; 4) pTSD1 plasmid, carrying the pyrE2 expression cassette, linearized with EcoRI. The pTSD1 suicide vector was created from a Gibson assembly consisting of a PCR fragment with primers oBL536 and oBL537 from the pTA131 vector, inserting an EcoRI restriction site upstream to the pyrE2 cassette. H26 cells were transformed, plated on Hv-Cab plates, and incubated at 45°C. Transformants were grown in liquid Hv-Cab overnight, back-diluted into Hv-Cab, and grown overnight. Cultures were then back diluted into Hv-Cab supplemented with 50 µM uracil, grown overnight, and then back diluted again in HvCab supplemented with 50 µM uracil. Cultures were struck onto Hv-Cab plates supplemented with 50 µM uracil and 50 µg/mL 5-FOA. Colonies were confirmed by PCR for the presence of *ftsZ1mChartreuse* and the absence of untagged *ftsZ1*.

aOL39 [ΔpyrE2 pTA962::sec-spoIIIJ-HaloTag] was created by transforming the H26 strain with the eOL52 plasmid, cloned from a Gibson assembly consisting of four parts: 1) SEC fragment: PCR with primers oHV13 and oOL79 from a synthetic DNA template; 2) spoIIIJ fragment: PCR with primers oOL80 and oOL81 from a PY79 (*Bacillus subtilis*) gDNA template; 3) 30aaHaloTag: PCR with primers oBL354 and oBL318 from a synthetic DNA template; 4) pTA962 plasmid linearized with NdeI. H26 cells were transformed in Hv-Cab plates.

ID112 [ΔhdrB ΔpyrE2 ΔftsZ1 ΔftsZ2] was a gift from Iain Duggin (UT Sydney).

ID77 *[ΔhdrB ΔpyrE2 ΔftsZ1]* was a gift from Iain Duggin (UT Sydney).

Δ2528 [ΔpyrE2 ΔHVO_2528] was a gift from Jörg Soppa (Goethe-Universität).

Δ*aglB [ΔpyrE2 ΔtrpA tn::aglB]* was a gift from Jerry Eichler (Ben Gurion University)

Δ*aglE [ΔpyrE2 ΔtrpA tn::aglE]* was a gift from Jerry Eichler (Ben Gurion University)

Δ*aglJ [ΔpyrE2 ΔtrpA tn::aglJ]* was a gift from Jerry Eichler (Ben Gurion University)

Δ*aglG [ΔpyrE2 ΔtrpA tn::aglG]* was a gift from Jerry Eichler (Ben Gurion University)

Δ*aglI [ΔpyrE2 ΔtrpA tn::aglI]* was a gift from Jerry Eichler (Ben Gurion University)

Fig. S1. Different setups used for *Haloferax volcanii* live-cell imaging. (A) Schematic illustration of the ArcCell microfluidic device. Cells are trapped in the orange region. Media flows through the blue channels, diffusing into the orange region. The green region is an alternative outlet, allowing for the flow path to be manipulated into loading cells into the traps (left). Dimensions of the cell trapping region (middle). Dimensions of the pillars that create the height of the cell trapping region (right). Dimensions of the device were measured using 200 steps on a profilometer. **(B)** Cell solidity measurements of cells from liquid cultures (black), trapped in ArcCell (blue) or confined beneath 0.25%, 0.5%, and 1% agarose pads (pink gradient). **(C)** Storage moduli (E') of agarose pads measured by DMA. Data was fit under a second-order polynomial curve.

Fig. S2. Pad stiffness is not sufficient to trigger tissue development. (A-C) Schematic illustrating the experiment performed to decouple substrate rigidity and compression. **(A)** (Left) *Hvo* cells were placed on a coverslip (step 1) and confined with a 2.5% agarose pad (step 2). The same culture was placed atop the agarose pad (step 3) and allowed to develop for 24 hours, unconfined. A secondary coverslip was placed on the agarose pad (step 4). (Right) Phase contrast images of cells on the bottom or top of the pad. **(B)** (Left) Bilayer agarose pads were poured with 2.5% on the bottom and 0.25% agarose on top. *Hvo* cells were then placed on two separate coverslips (step 1). The cells were compressed with either the 2.5% (step 2a) or 0.25% (step 2b) agarose surface facing towards them. (Right) Phase contrast images of cells under the bilayer pad cakes' 2.5% or 0.25% pad surface. **(C)** *Hvo* cells were compressed between an agarose pad (top) and a thin agarose film coating the coverslip (bottom).

Fig. S3. Tissue development is a precisely controlled process independent of pad weight, mass, and thickness. (A) (left) Illustration of *Hvo* cells compressed by pads in upright and upside-down positions. (right) Phase-contrast images of tissues after 24 hours of incubation at 42°C. **(B)** *Hvo* cells were compressed under pads with different agarose concentrations and masses. Pad thickness was scaled with masses as their areas were kept the same. **(C)** Cell area at cellularization is independent of agarose concentration above the 2% threshold.

Figure S4. Tissues maintain intact S-layer lattices but show distinct viscoelastic interaction properties. **(A)** Phase-contrast micrographs of *Hvo* cells fragmented from tissues (left) and slices through a tomogram of a *Hvo* unicell (top-right) and a *Hvo* cell from fragmented tissues (bottom-right), highlighting the cell membrane, S-layer and the distance measured between them, which agrees with previously published data (~12 nm distance from the membrane to the S-layer base and a further 8-10 nm thickness of the S-layer) (76). Scale bars: 100 nm. **(B)** Epifluorescence microscopy of *Hvo* unicells stained with Brilliant Blue (S-layer, magenta) and SYBR (DNA, green). (Left) Control cells from cultures in Hv-Cab medium. (Middle) Spheroplasting under 50 mM EDTA (10-minute incubation) disrupts the Slayer lattice but does not detach the S-layer glycoprotein from the cytoplasmic membrane. Cells under EDTA treatment show high heterogeneity in DNA staining. (Right) Spheroplasting under 50 mM EDTA and 1 mg/ml Proteinase K degrades outer-surface proteins and prevents Brilliant Blue staining. **(C)** (Left) Displacement vectors of cells' center of mass in response to laser ablation in the center of unicells (left) and a tissue (middle). (Right) Segmented cell masks and displacement tracks in a representative tissue. False-colored vectors and tracks were normalized from MSD values.

Fig. S5. Evolutionary diversity of haloarchaeal tissues based on core genome. The cladogram represents the evolutionary relationship between 315 Halobacteria genomes, estimated from the concatenation of 385 core genes (see Supplementary Methods for details on gene selection and alignment procedure). Ultrafast bootstrap approximation (UFBoot) and the approximate likelihood-ratio test (aLRT), used as bipartition support values, are shown for nodes that do not equal 100. Taxa are color-coded according to identified multicellular traits: red reflects taxa that multicellularity was not observed, blue denotes taxa with tissue organization, and green reflects taxa displaying sarcina-like aggregate structures. Collapsed taxa shown in grey have not been assessed in this study. For clarity in presentation, branch lengths are not depicted, hence the cladogram, and clades are composed of not-evaluated taxa. These traits are collapsed, indicated by a shorter tip length. Detailed information regarding tree topology, branch lengths, and full bipartition support values can be found in Supplementary Data S1.

Fig. S6: Diversity of responses other than tissue development of haloarchaeal cells to compression. **(A)** Correlation between unicell size (prior to compression) and doubling time from liquid cultures of tested haloarchaeal species. **(B-E)** Phase contrast images of **(B)** *Halorubrum tebenquichense*, **(C)** *Halobacterium salinarum*, **(D)** *Halococcus salifodinae*, and **(E)** *Natronococcus jeotgali* under compression.

Fig. S7. Diversity of archaeal tissue structures and biofilm production across *Haloferax* species. (A) SoRa super-resolution microscopy images of 11 *Haloferax* species developed under compression. Tissues shown in *Hfx. volcanii* (blue frame). *Hfx. mediterranei* and *Hfx. gibbonsii* (red frames) cannot form tissues and *Hfx. prahovense* (yellow frame) forms larger, deformed tissues. **(B)** (Top) Biofilm mass measurements from *Haloferax* species *Hmed* display markedly high biofilm formation relative to other tested *Haloferax* species. Representative photography of *Hvo* (bottom-left) and *Hmed* (bottom-right) colonies show the pale aspect of *Hmed* compared to *Hvo*. **(C)** Viability of *Hvo* tissues compared to *Hvo* cells from isolated colonies from plate streaks measured by colony formation unit (CFU). CFUs were normalized by liquid cultures used as initial inoculum.

Fig. S8. Apicobasal cells interact directly with pads, allowing peribasal cells to escape mechanical compression. (A) Traction force microscopy setup using fluorescent beads attached to the pad's surface compressing cells. Epifluorescence z-stacks of bead displacement exclusively on top of apicobasal cells at the time of compression and after 24 hours. Images are false-colored in magenta (bottom focal plane) and green (top focal plane) **(B)** Surface area and volume measurements of apicobasal and peribasal cells from 3D projections.

Fig. S9. FtsZ1-mChartreuse is a functional fusion that localizes to the tissues' nascent junctions. (A) Phase contrast images of (top) wild-type and (middle) Δ*ftsZ1* mutant cells. (bottom) Phase contrast and epifluorescence overlay of cells expressing FtsZ1-mChartreuse under the control of its native promoter. **(B)** SoRa super-resolution microscopy images of tissue junction formation from the top and side perspective of the 3D projection in 10-minute intervals (at 0, 10, 20, and 30 min). The white arrow indicates a nascent cell junction where the FtsZ1-mChartreuse (green) constricts together with the S-layer (magenta).

Fig. S10. Membrane fluidity impacts viability during multicellular development. (A) (Left) Sum of msfGFP-PCNA fluorescence intensity within foci in a cell normalized by cell area between unicells and coenocytes at the onset of cellularization. (Right) The number of msfGFP-PCNA foci in each cell normalized by cell area in unicells versus coenocytes at the onset of cellularization. **(B)** Mean Squared Displacement (MSD) vs time lag (τ) for individual full-length bSpoJ tracks ($R^2 \geq 0.9$) from unicells and coenocytes. Both axes are plotted with logarithmic scaling. **(C)** (Left) Growth curves of wild-type and *car*⁻ liquid cultures at 42°C or 34°C. (Right) Phase contrast images of wild-type and car− unicells from liquid cultures at 42°C or 34°C. **(D)** (Left) Spectral confocal microscopy of representative wild-type spheroplasts stained with 100µM Laurdan at different wavelengths. (Right) Blue-shift emission spectra from confocal images of wild-type and car− cells at lower temperatures, indicating a decrease in membrane fluidity. **(E)** Normalized survival rates of wild-type and car− cells across temperatures. Survival was determined by cell lysis events from phase-contrast time lapses. **(F)** Quantification of carotenoid production in *Hvo* and *Hmed*, showing higher production of pigments by *Hvo*.

Fig. S11. Archaeal tissue development correlates with carotenoid production across *Haloferax* species. Colonies of twelve *Haloferax* strains on Hv-Cab agar plates. Colonies from species that do not develop into archaeal tissues (*Hfx. mediterranei*, red frame) have the lowest observed red coloration, whereas colonies from *Hfx. prahovense* and *Hfx. gibbonsii* (yellow frame), that form tissues with unstable junctions, show lower red coloration than other species.

Fig. S12. Temporal and genomic patterns observed from RNA-seq during Multicellular Development. **(A)** Venn diagram with the number of downregulated (left) and upregulated (right) genes in each stage. **(B)** Number of differentially expressed gene candidates grouped by quadrant of the main chromosome of *Hvo* across each (top-left) or all (top-right) developmental stages. Number of differentially expressed gene candidates normalized by the number of total genes in each quadrant of the main chromosome of *Hvo* across each (bottom-left) or all (bottom-right) developmental stages. **(C)** The modulus of expression fold-change values from both upregulated and downregulated gene candidates mapped to each quadrant of the main chromosome of *Hvo*. A significant difference in the amplitude of gene expression was observed only in tissues, specifically in the third chromosomic quadrant. **(D)** Venn diagram of shared and exclusive ortholog distribution from comparative genomic analysis for *Hvo* (blue) and *Hmed* (orange). **(E)** Venn diagram of enrichment ortholog distribution between *Hvo* (blue), *Hmed* (orange), *Hfx. gibbonsii* (*Hgib*; green), and *Hfx. prahovense* (*Hpra*; yellow).

Fig. S13. Polarity patterns of N-Glycosylation are tissue-specific and independent of biofilm. (A) Spinning-disk confocal images of unicells and archaeal tissues across mutant strains stained with ConAAlexa488 **(B)** Normalized ConA-Alexa488 fluorescence intensity across wild-type (black) and Δ*aglB* (blue) tissues. Normalized distances across tissues were determined by the longest diameter measurement. Scale bars: 10 µm.

Table S1. *Haloferax* species and RefSeq genome accessions used.

Species	RefSeq	Assembly	Forms Arc-Tissues
Haloferax alexandrinus	GCF_030535215.1	ASM3053521v1	Yes
Haloferax denitrificans	GCF_000337795.1	ASM33779v1	Yes
Haloferax elongans	GCF_000336755.1	ASM33675v1	Yes
Haloferax gibbonsii	GCF_000336775.1	ASM33677v1	**No**
Haloferax larsenii	GCF_000336955.1	ASM33695v1	Yes
Haloferax lucentense	GCF_000336795.1	ASM33679v1	Yes
Haloferax mediterranei	GCF_033843025.1	ASM3384302v1	**No**
Haloferax mucosum	GCF_000337815.1	ASM33781v1	Yes
Haloferax prahovense	GCF_000336815.1	ASM33681v1	***Yes**
Haloferax sulfurifontis	GCF_014635105.1	ASM1463510v1	Yes
Haloferax volcanii	GCF_000025685.1	ASM2568v1	Yes

*Cells cellularize into larger and unorganized tissues

Table S2. Contingency table used in the hypergeometric test that allows for classification of proteins per class according to a given orthogroup. N is the total protein population; K is the number of proteins issuing from the cohort of species that form tissue; n is the total number of proteins in an orthogroup; and k is the number of proteins from an orthogroup that issue from the group of species that form tissue.

	**∈ orthogroup**	**∉ orthogroup**	**total**
**∈ sp. that forms tissue**	k	$K - k$	K
**∉ sp. that forms tissue**	$n - k$	$N + k - n - K$	$N - K$
**total**	n	$N - n$	N

Table S3. Plasmids used in this work

Alias	Genotype	Reference
pTA962	pTA962 (PtnA *pyrE2 hdrB ori-pHV2 oriC*)	(99)
pTA131	pTA131 (*pyrE2*)	(99)
eTR68	pTSD1 (*pyrE2*)	This work
eBL416	pTDS1::ftsZ1-40aa-mChartreuse	This work
eSB14	pTA131::mevR-HaloTag-15aa-mNG-40aa-PCNA	This work
eOL52	pTA962::sec-spoIIIJ-HaloTag	This work

Table S4. Haloarchaeal strains used in this work

Alias	Genotype	Growth Media	Reference
ATCC 33960	*Haloarcula hispanica*	372-Cab	(100)
NRC-1	*Halobacterium salinarum*	HCM	(101)
ATCC 33500	*Haloferax mediterranei*	HV-Cab	(102)
DSM 4427	*Haloferax gibbonsii*	HV-Cab	(100)
DSM 27206	*Haloferax alexandrinus*	HV-Cab	(103)
DSM 4425	*Haloferax denitrificans*	HV-Cab	(104)
DSM 14919	*Haloferax lucentense*	HV-Cab	(105)
DSM 16227	*Haloferax sulfurifontis*	HV-Cab	(106)
DSM 18310	*Haloferax prahovense*	HV-Cab	(107)
DSM 26526	*Haloferax chudinovii*	HV-Cab	(108)
DSM 27190	*Haloferax larsenii*	HV-Cab	(109)
DSM 27191	*Haloferax mucosum*	HV-Cab	(110)
DSM 27209	*Haloferax elongans*	HV-Cab	(110)
DSM 11551	*Halogeometricum borinquense*	372-Cab	(111)
DSM 23171	*Halogranum salarium*	HV-Cab	(112)
DSM 17463	*Halorubrum ezzemoulense*	372-Cab	(113)
DSM 10284	*Halorubrum coriense*	372-Cab	(114)
DSM 21996	*Halorubrum xinjiangense*	372-Cab	(115)
DSM 23453	*Halorubrum distributum*	372-Cab	(116)
DSM 12287	*Halorubrum trapanicum*	372-Cab	(117)
DSM 14210	*Halorubrum tebenquichense*	372-Cab	(118)
DSM 1137	*Halorubrum saccharovorum*	372-Cab	(119)
DSM 19804	*Halorubrum kocurii*	372-Cab	(120)
DSM 21995	*Halorubrum lipolyticum*	372-Cab	(121)
DSM 5036	*Halorubrum lacusprofundi*	372-Cab	(122)
DSM 23496	*Halorubrum aidingense*	372-Cab	(121)
DSM 18322	*Halorubrum aquaticum*	372-Cab	(123)
DSM 19504	*Halorubrum cibi*	HV-Cab	(124)
DSM 22472	*Halostagnicola kamekurae*	372-Cab	(125)
DSM 3751	*Natrinema pallidum*	372-Cab	(126)
DSM 15624	*Natrinema pellirubrum*	372-Cab	(126)
DSM 18796	*Halalkalicoccus jeotgali*	372-Cab	(127)
DSM 18795	*Natronococcus jeotgali*	372-Cab	(128)

DSM 18794	*Haloterrigena jeotgali*	372-Cab	(129)
DSM 11552	*Haloterrigena thermotolerans*	372-Cab	(130)
DSM 25055	*Natrinema salaciae*	372-Cab	(131)
DSM 18328	*Haloterrigena hispanica*	372-Cab	(132)
DSM 26231	*Halopiger salifodinae*	372-Cab	(133)
DSM 5511	*Haloterrigena turkmenica*	372-Cab	(134)
DSM 13077	*Natrialba aegyptia*	372-Cab	(135)
DSM 12281	*Natrialba taiwanensis*	372-Cab	(135)
DSM 12278	*Natrialba asiatica*	372-Cab	(136)
DSM 10524	*Natronococcus amylolyticus*	372-Cab	(137)
DSM 14522	*Halococcus dombrowskii*	372-Cab	(138)
DSM 1307	*Halococcus morrhuae*	372-Cab	(139)
DSM 8989	*Halococcus salifodinae*	372-Cab	(140)
DSM 5350	*Halococcus saccharolyticus*	372-Cab	(141)
DSM 3756	*Haloarcula vallismortis*	372-Cab	(142)
DSM 6131	*Haloarcula japonica*	372-Cab	(143)
DSM 12282	*Haloarcula argentinensis*	372-Cab	(144)
DSM 3752	*Haloarcula marismortui*	372-Cab	(145)

Table S5. ***Haloferax volcanii*** **strains used in this work**

Alias	Genotype	Reference
DS2	*Haloferax volcanii*	(146)
H26	Δ*pyrE2*	(147)
H53	Δ*pyrE2* Δ*trpA*	(147)
aBL126	Δ*pyrE2 volA::volA-40aa-msfGFP-pyrE2*	(44)
aKA17	Δ*pyrE2* Δ*volA*	(45)
ID112	Δ*hdrB* Δ*pyrE2* Δ*ftsZ1* Δ*ftsZ2*	(148)
ID77	Δ*pyrE2* Δ*ftsZ2*	(148)
Δ2528	Δ*pyrE2* Δ*HVO_2528*	(149)
MT4	Δ*pibD*	(150)
ΔaglB	Δ*pyrE2* Δ*trpA tn::aglB*	(151)
ΔaglE	Δ*pyrE2* Δ*trpA tn::aglE*	(151)
ΔaglJ	Δ*pyrE2* Δ*trpA tn::aglJ*	(152)
ΔaglG	Δ*pyrE2* Δ*trpA tn::aglG*	(152)
ΔaglI	Δ*pyrE2* Δ*trpA tn::aglI*	(153)
aBL582	Δ*pyrE2* ftsZ1::ftsZ1-40aa-mChartreuse	This work
aSB14	*pcna::mevR-HaloTag-15aa-mNG-40aa-pcna*	This work
aOL39	Δ*pyrE2* pTA962::sec-spoIIIJ(TM)-HaloTag	This work

Table S6. Oligos used in this work

Alias	Sequence (5’ → 3’)
oHV13	CGGACCTATTGCGCATATGACAAAGCTCAAAGATCAAACG
oAB130	GCCTTGACCTGGGCCAGATC
oBL97	AGGTGGCACTTTTCGG
oBL105	TGAGCAAAAGGCCAGC
oBL318	GGAATTCGATATCAAGCTTATCGATTTTTAGCCGCTGATTTCTAAGGTAG
oBL354	CTTGAGGGTAGCGGAC
oBL532	GATATCGAATTCCTGCAGCCTCGAGCCGTCCCG
oBL533	CGAGGGGTTTTATCCACGCGAAGAAACGGTTTTGTGG
oBL536	GAAAAGTGCCACCTGAATTCACATGCATGGGGGGTTG
oBL537	GGGTTTTATCCACGGAATTCCATGAGCTTCTTTGATTCGAGC
oOL79	GGCTCTTTCACGAACGATTCGCTGTCAG
oOL80	GTGAAAGAGCCGATCACTG
oOL81	GCTACCCTCAAGCTTTTTCTTTCCTCCGGCTTTT
oTR202	CCGAAAAGTGCCACCTCGGCCACGCGGTC
oTR203	CCTACGACGCGTGAGGTATGGCGCGCCGGTCCGAG
oTR204	CGCCATACCTCACGC
oTR205	CGAGGGGTTTTATCCACGGCCCGGTTCGGGT
oSB41	CCGAAAAGTGCCACCTCGTGACGCTCGCG
oSB42	CCATCCCCTCCCATGATGTGCCGTCGTACG

Table S7. Statistical Information from Datasets in this Study.

Figure	Experiment	N Samples (Biological Replicate)	Statistical Test
1G	Unicell Ablation Displacement	119 (14,48,67)	Kolmogorov-Smirnov
	Tissue Ablation Displacement	92 (12,42,38)	
1H	Unicell Ablation Junction Recoil	22 (6,13,3)	-
	Tissue Ablation Junction Recoil	24 (4,12,6)	
2E	*Hvo* x Hmed Survival	3 (1,1,1)	One-Way ANOVA
3D	Unicell Growth Rate	942 (333,278,341)	Kolmogorov-Smirnov
	Peribasal Growth Rate	172 (56,74,42)	
	Apicobasal Growth Rate	205 (99,63,43)	
3E	Unicell Lifespan	896 (211,405,280)	Kolmogorov-Smirnov
	Peribasal Lifespan	99 (52,25,22)	
	Apicobasal Lifespan	191 (87,40,64)	
3F	Unicell Deformation	337 (109,58,170)	Kolmogorov-Smirnov
	Peribasal Deformation	46 (23,9,14)	
	Apicobasal Lifespan	121 (25,82,14)	
4C	WT Cellularization	241 (73,90,78)	Kolmogorov-Smirnov
	ΔftsZ2 Cellularization	164 (33,69,62)	
4D	Unicell bSpoJ Diffusion	1782 (1023,455,304)	Kolmogorov-Smirnov
	Coenocyte bSpoJ bSpoJ Diffusion	1875 (521,179,1175)	
4E	WT Generalized Polarization	1455 (654,324,477)	-
	Car− Generalized Polarization	1199 (560,340,299)	
4F	WT Cellularization Area at 34°C	106 (69,26,11)	Kolmogorov-Smirnov
	WT Cellularization Area at 37°C	131 (45,24,52)	
	WT Cellularization Area at 42°C	162 (39,102,21)	
	Car− Cellularization Area at 34°C	77 (26,19,32)	
	Car− Cellularization Area at 37°C	125 (59,23,43)	
	Car− Cellularization Area at 42°C	179 (91,26,62)	
4G	WT Unicell bSpoJ Diffusion	2522 (1306,560,656)	Kolmogorov-Smirnov
	WT Coenocyte bSpoJ Diffusion	1965 (1048,753,164)	
	WT Cellularization bSpoJ Diffusion	1358 (509,515,334)	
	Car− Unicell bSpoJ Diffusion	2114 (734,251,1129)	
	Car− Coenocyte bSpoJ Diffusion	987 (735,201,51)	
	Car− Cellularization bSpoJ Diffusion	793 (282,405,106)	
5B	Volactin-GFP Unicell Fluorescence	103 (21,44,38)	-
	Volactin-GFP Tissue Fluorescence	42 (9,16,17)	
	GFP Tissue Fluorescence	35 (10,5,20)	
5D	Volactin Unicell Cable Angle	172 (41,62,71)	Kolmogorov-Smirnov
	Volactin Coenocyte Cable Angle	94 (33,16,45)	

	Volactin Tissue Cable Angle	79 (18,32,29)	
5E	WT Coenocyte Height:Area Ratio	152 (43,67,42)	Kolmogorov-Smirnov
	Δ*volA* Coenocyte Height:Area Ratio	117 (22,52,43)	
S1B	Cell Solidity (Liquid Bulk Culture)	931 (255,245,431)	-
	Cell Solidity (Microfluidics)	1525 (350, 500, 675)	
	Cell Solidity (0.25% Agarose Pad)	134 (11,56,67)	
	Cell Solidity (0.5% Agarose Pad)	102 (34,23,45)	
	Cell Solidity (1% Agarose Pad)	91 (20,42,39)	
S1C	DMA Agarose Pads	3 (1,1,1)	-
S2C	Cellularization Cell Area (2% Agarose Pad)	211 (50,50,111)	Kolmogorov-Smirnov
	Cellularization Cell Area (2.5% Agarose Pad)	198 (50,50,98)	
	Cellularization Cell Area (3% Agarose Pad)	185 (50,50,85)	
	Cellularization Cell Area (3.5% Agarose Pad)	201 (50,50,101)	
S7B	*Hfx* Species Biofilm Mass	3 (1,1,1)	One-Way ANOVA
S7C	*Hvo* Colonies x Tissues Survival	3 (1,1,1)	One-Way ANOVA
S8B	Peribasal Cell Surface:Area	120 (63,32,25)	Kolmogorov-Smirnov
	Apicobasal Cell Surface:Area	230 (77,64,89)	
S10A	Unicell PCNA Fluorescence	158 (42,51,55)	Kolmogorov-Smirnov
	Cellularization PCNA Fluorescence	125 (37,65,23)	
S10C	WT x Car− Growth Curves	3 (1,1,1)	-
S10E	WT Coenocyte Survival at 34°C	154 (67,42,35)	Kolmogorov-Smirnov
	Car− Coenocyte Survival at 34°C	91 (30,50,11)	
	Car− Coenocyte Survival at 37°C	138 (25,39,74)	
	Car− Coenocyte Survival at 42°C	112 (54,33,35)	
S10F	*Hvo* x *Hmed* Carotenoid Fraction	3 (1,1,1)	-
S13B	WT ConA-Alex488 Localization	42 (12,21,9)	-
	Δ*aglB* ConA-Alex488 Localization	61 (31,19,11)	

Movie S1. *Hfx. volcanii* unicell growth within ArcCell microfluidics and under agarose pads. Phase-contrast microscopy timelapses in ArcCell (left panel) and under 0.25% agarose pads (right panel). Timelapses were acquired at 2- and 5-min intervals, respectively. Movies are displayed at 8 frames per second. Scale bars: 5µm

Movie S2. Archaeal tissue development under compression. Epifluorescence (left panel) and phase-contrast (right panel) microscopy of cells growing under compression by a 2.5% Hv-Cab agarose pads. Timelapses collected at 20-min intervals and displayed at 5 frames per second. S-layer stained with Brilliant Blue. Scale bar: 5µm

Movie S3. DNA replication carries on during the coenocytic phase. Epifluorescence microscopy of mNeonGreen-PCNA (DNA sliding clamp, cyan) foci decorating replication sites. Middle panel: phase contrast. Right panel: mNeonGreen-PCNA. Left panel: overlay. Timelapses were collected at 2-min intervals and displayed at 20 frames per second. Scale bar: 5µm

Movie S4. 3D-SoRa super-resolution microscopy of tissues versus unicellular colonies. 3D projections from the central regions of a clustered unicell colony (left panel) and tissue (right panel). Z-stacks were collected at 0.1-µm slices. S-layer stained with Brilliant Blue. Scale bar: 5µm.

Movie S5. Wounding of archaeal tissues induces directional stretching of cell junctions towards the injured site. Central regions of unicell clusters (left panel) and tissues (right panel) were ablated by a 405 nm laser pulse (darker). Following injury, samples were imaged by SoRa super-resolution microscopy at 2.5-min intervals. For the tissue movie, a Z-stack projection with 2 slices separated by 0.5 µm is shown. Movies are displayed at 5 frames per second. S-layer stained with Brilliant Blue. Scale bar: 5µm.

Movie S6. Cell membrane recoil of unicells and tissues by laser ablation. Cytoplasmic membrane of unicells (left panel) and junctions within archaeal tissues (right panel) ablated with 405nm laser pulse. Following severing, samples were imaged by SoRa super-resolution microscopy at 1-second (unicells) and 100-ms (tissues) intervals. Movies are displayed at 10 frames per second. S-layer stained with Brilliant Blue. Scale bar: 5µm.

Movie S7. *Hfx. mediterranei* cells do not form tissues and "swarms out" out compression zones. Phase-contrast microscopy of *Hmed* cells growing under 3% (left panel) and 5% (right panel) agarose pads were collected at 15-min intervals. Movies are displayed at 5 frames per second. Scale bar: 5µm.

Movie S8. *Hfx.* cells growth under microfabricated "escape-room" pillar traps. Phase-contrast imaging of *Hvo* (right panel) and *Hmed* (left panel) cells were acquired at 10- and 1-min intervals, respectively. Movies are displayed at 5 frames per second. Scale bars: 10µm.

Movie S9. Reversible Rod-shape transition after recovery from tissue shear-fragmentation. (Left Panel) Phase-contrast timelapse of tissues being shear-shocked by a liquid wave (false-colored in magenta), followed by fast development (under a generation time) to swimming rods. (Right Panel) Zoom-in of a second tissue structure transition to rods followed by fragmentation. Movies were collected at 1-min intervals and displayed at 10 frames per second. Scale bar: 10µm.

Movie S10. Super-resolution microscopy of peribasal and apicobasal cells. (Top-Left Panel) 3D-STED projection of cells stained with Brilliant Blue acquired with 75-nm spaced Z slices. (Top-Right Panel) 3D

outline masks of apicobasal scutoid cells segmented from 3D-STED projections. (Bottom panels) 3D-iSIM projection of cells constitutively expressing cytoplasmic msfGFP acquired with 200-nm spaced Z slices. Scale bars: 5μm.

Movie S11. Tissue recompression followed by shear-fragmentation. Phase-contrast timelapse of tissues being disrupted by liquid shear shock, followed by removal of liquid media and recompression of fragmented cells. Movies were collected at 1-min intervals and displayed at 20 frames per second. Scale bar: 10μm.

Movie S12. Single-molecule tracking of the membrane-associated chimera bSpoJ-JFx549 conjugates in coenocytes. Total Internal Reflection Fluorescence Microscopy (TIRFM) of coenocytes (shown in phase contrast) expressing bSpoJ conjugated with JFx549 dyes imaged at 0.25-sec intervals. Movies are displayed at 40 frames per second. Tracks (right panel) are false-colored in a mean speed scale between 0.1 (blue) and 2 (red) μm/s. Scale bar: 5μm.

Movie S13. Single-molecule tracking of the membrane-associated chimera bSpoJ-JFx549 conjugates in unicells. Total Internal Reflection Fluorescence Microscopy (TIRFM) of unicells (shown in phase contrast) expressing bSpoJ conjugated with JFx549 dyes imaged at 500-ms intervals. Movies are displayed at 10 frames per second. Tracks (right panel) are false-colored according to their mean speed scale between 0.1 (blue) and 2 (red) μm/s. Scale bar: 5μm.

Movie S14. Volactin endogenous expression increases as cells differentiate into tissues.
Epifluorescence microscopy of volA-msfGFP under native promoter shows an increase in the fluorescence signal as cells differentiate into tissues. The S-layer is stained with Brilliant Blue. The scale bar is 5 5μm.

Movie S15. Volactin dynamics in tissues. Epifluorescence microscopy of volA-msfGFP acquired at 25-min intervals and displayed at 4 frames per second. Scale bar: 5μm.

Movie S16. Volactin cables relative orientation during development. 3D-SoRa super-resolution representative projections of volA-msfGFP cables in unicells (first/left panel), coenocytes (second panel), coenocytes during cellularization (third panel), and tissues (fourth/right panel). S-layer stained with Brilliant Blue. Z-stacks collected under 0.1-μm intervals. Scale bars: 5μm.

Movie S17. Protein Glycosylation patterns in wild-type and Δ*aglB* tissues. 3D projections of tissues showing S-layer (left panel, black) and protein glycosylation (right panel, blue) spatial profiles. Spinning-disk confocal Z-stacks were collected under 0.1-μm intervals. Glycoproteins are stained with ConA-Alexa 488. S-layer is stained with Brilliant Blue. Scale bars: 5μm.

Data S1. Maximum likelihood phylogenetic tree of Haloarchaea with branch support values (JSONformatted TreeFile/FigTree).

Data S2. Differentially gene expression (DGE) candidates across developmental stages (XLSXformatted spreadsheet). (A) All candidates above arbitrary threshold. (B) Shortlisted genes with possible role in development. Blue: upregulated. Orange: downregulated.

Data S3. *Hfx. volcanii* unique genes compared to *Hfx. mediterranei* (XLSX-formatted spreadsheet).

Data S4. *Hfx. volcanii* unique genes compared to *Hfx. mediterranei, Hfx. gibbonsii, and Hfx. prahovense* (XLSX-formatted spreadsheet).

Data S5. Enrichment of orthologs among Hfx species that form tissues relative to *Hfx. mediterranei, Hfx. gibbonsii, and Hfx. prahovense* (XLSX-formatted spreadsheet)

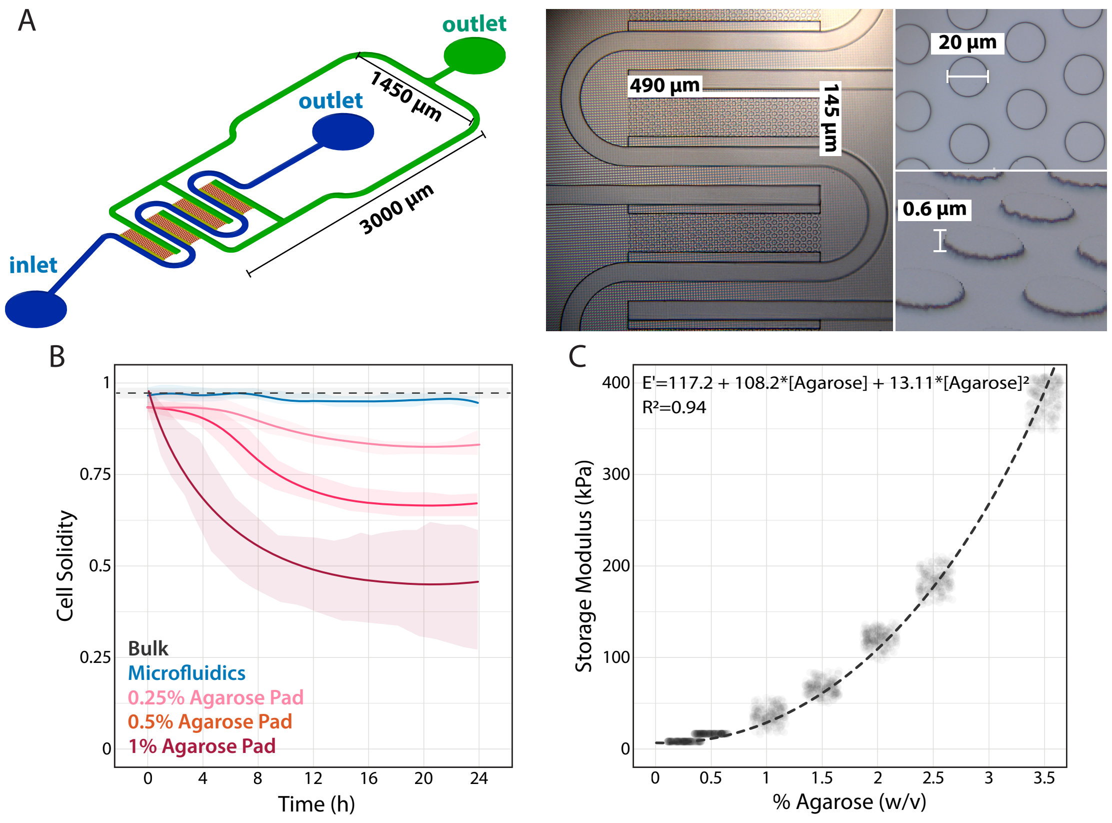
A
outlet
outlet
1450 µm
3000 µm
inlet
490 µm
145 µm
20 µm
0.6 µm
B
Cell Solidity
Time (h)
Bulk
Microfluidics
0.25% Agarose Pad
0.5% Agarose Pad
1% Agarose Pad
C
E'=117.2 + 108.2*[Agarose] + 13.11*[Agarose]²
R²=0.94
Storage Modulus (kPa)
% Agarose (w/v)

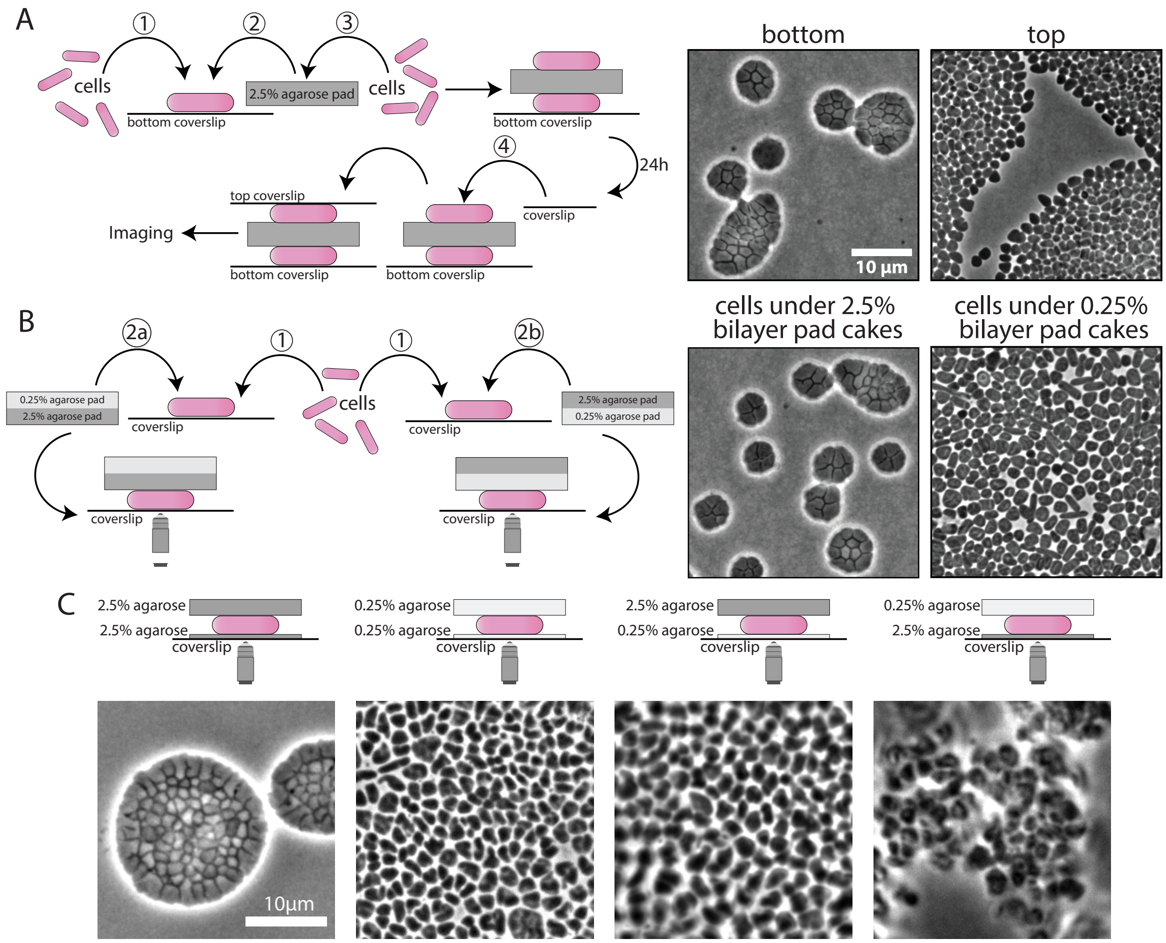

A
cells
2.5% agarose pad
cells
bottom coverslip
bottom coverslip
24h
top coverslip
coverslip
Imaging
bottom coverslip
bottom coverslip
bottom
top
10 µm
B
0.25% agarose pad
2.5% agarose pad
coverslip
cells
coverslip
2.5% agarose pad
0.25% agarose pad
coverslip
coverslip
cells under 2.5% bilayer pad cakes
cells under 0.25% bilayer pad cakes
C
2.5% agarose
2.5% agarose
coverslip
0.25% agarose
0.25% agarose
coverslip
2.5% agarose
0.25% agarose
coverslip
0.25% agarose
2.5% agarose
coverslip
10µm

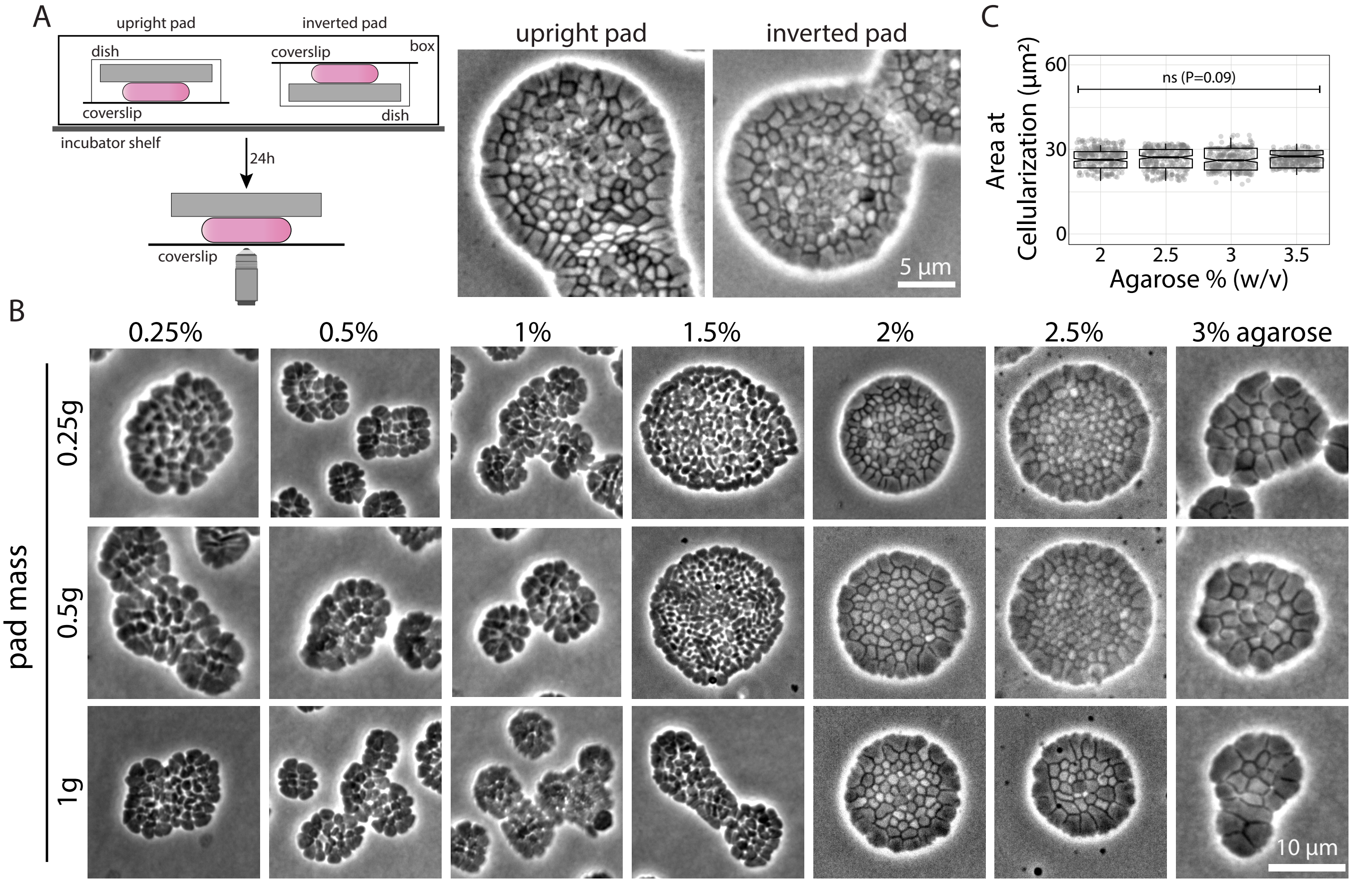
A
upright pad
inverted pad
dish
box
coverslip
coverslip
dish
incubator shelf
24h
coverslip
upright pad
inverted pad
5 µm
C
ns (P=0.09)
Area at Cellularization (µm²)
60
30
0
2
2.5
3
3.5
Agarose % (w/v)
B
0.25%
0.5%
1%
1.5%
2%
2.5%
3% agarose
pad mass
0.25g
0.5g
1g
10 µm

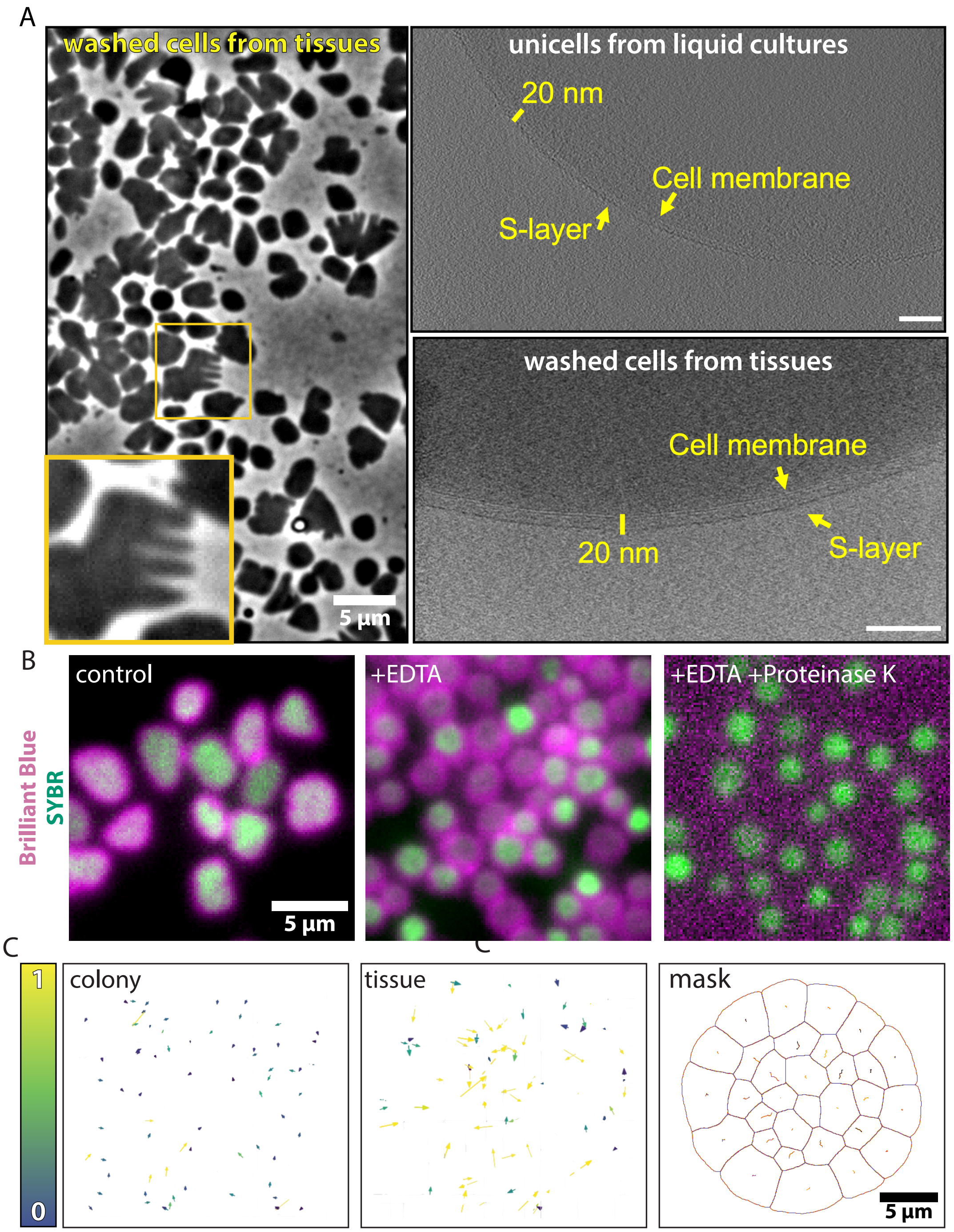
A
washed cells from tissues
5 µm
unicells from liquid cultures
20 nm
Cell membrane
S-layer
washed cells from tissues
Cell membrane
20 nm
S-layer
B
Brilliant Blue
SYBR
control
5 µm
+EDTA
+EDTA +Proteinase K
C
1
0
colony
tissue
mask
5 µm

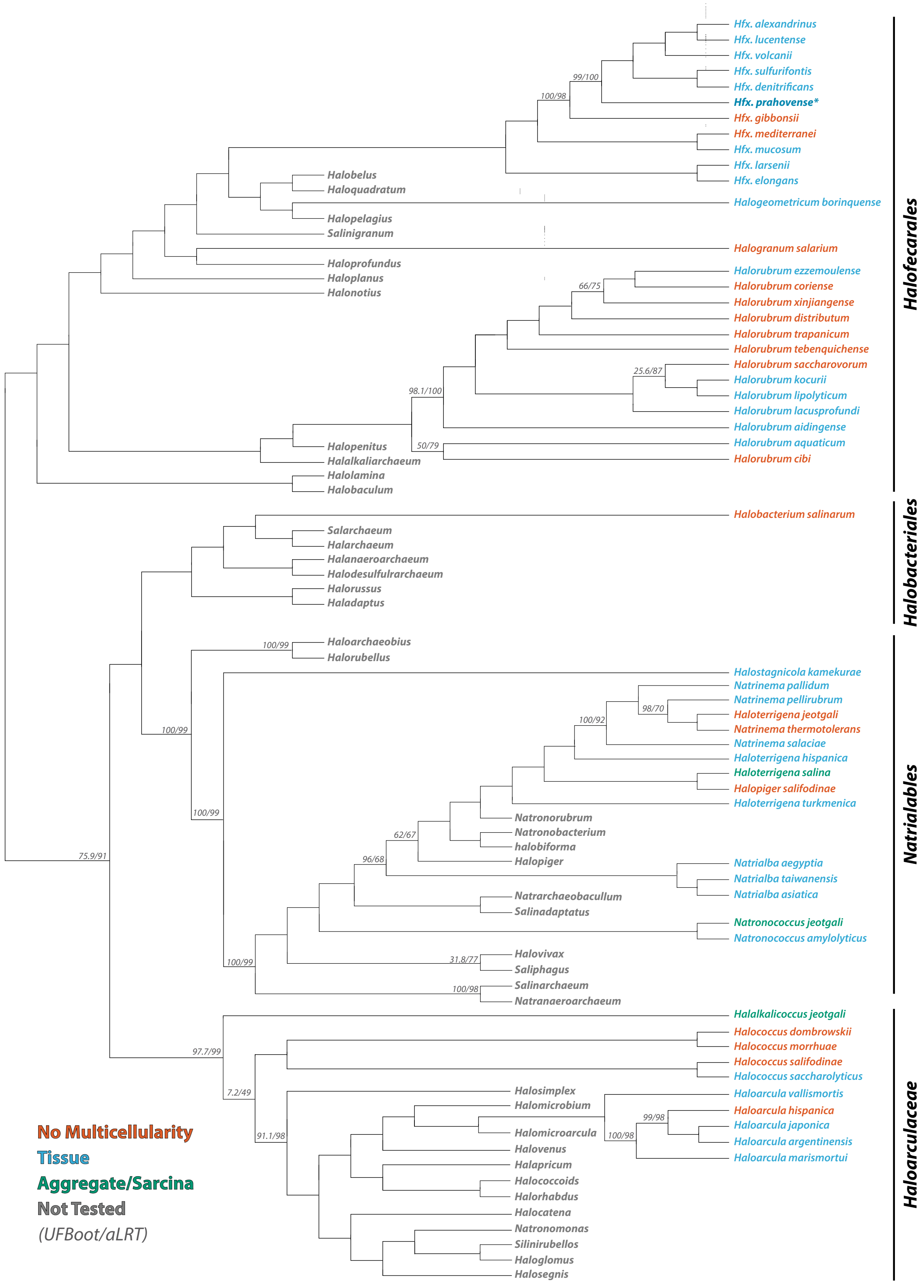

Hfx. alexandrinus
Hfx. lucentense
Hfx. volcanii
Hfx. sulfurifontis
Hfx. denitrificans
Hfx. prahovense*
Hfx. gibbonsii
Hfx. mediterranei
Hfx. mucosum
Hfx. larsenii
Hfx. elongans
99/100
100/98
Halobelus
Haloquadratum
Halogeometricum borinquense
Halopelagius
Salinigranum
Halogranum salarium
Haloprofundus
Haloplanus
Halonotius
66/75
Halorubrum ezzemoulense
Halorubrum coriense
Halorubrum xinjiangense
Halorubrum distributum
Halorubrum trapanicum
Halorubrum tebenquichense
25.6/87
Halorubrum saccharovorum
Halorubrum kocurii
Halorubrum lipolyticum
Halorubrum lacusprofundi
98.1/100
Halorubrum aidingense
50/79
Halorubrum aquaticum
Halorubrum cibi
Halopenitus
Halalkaliarchaeum
Halolamina
Halobaculum
Halofecarales
Halobacterium salinarum
Salarchaeum
Halarchaeum
Halanaeroarchaeum
Halodesulfurarchaeum
Halorussus
Haladaptus
Halobacteriales
100/99
Haloarchaeobius
Halorubellus
Halostagnicola kamekurae
Natrinema pallidum
98/70
Natrinema pellirubrum
100/92
Haloterrigena jeotgali
Natrinema thermotolerans
Natrinema salaciae
Haloterrigena hispanica
Haloterrigena salina
Halopiger salifodinae
Haloterrigena turkmenica
100/99
100/99
Natronorubrum
Natronobacterium
halobiforma
62/67
Halopiger
96/68
Natrialba aegyptia
Natrialba taiwanensis
Natrialba asiatica
75.9/91
Natrarchaeobacullum
Salinadaptatus
Natronococcus jeotgali
Natronococcus amylolyticus
31.8/77
Halovivax
Saliphagus
100/99
100/98
Salinarchaeum
Natranaeroarchaeum
Natrialables
Halalkalicoccus jeotgali
97.7/99
Halococcus dombrowskii
Halococcus morrhuae
Halococcus salifodinae
Halococcus saccharolyticus
7.2/49
Halosimplex
Halomicrobium
Haloarcula vallismortis
99/98
Haloarcula hispanica
Haloarcula japonica
91.1/98
Halomicroarcula
100/98
Haloarcula argentinensis
Haloarcula marismortui
Halovenus
Halapricum
Halococcoids
Halorhabdus
Halocatena
Natronomonas
Silinirubellos
Haloglomus
Halosegnis
Haloarculaceae
No Multicellularity
Tissue
Aggregate/Sarcina
Not Tested
(UFBoot/aLRT)

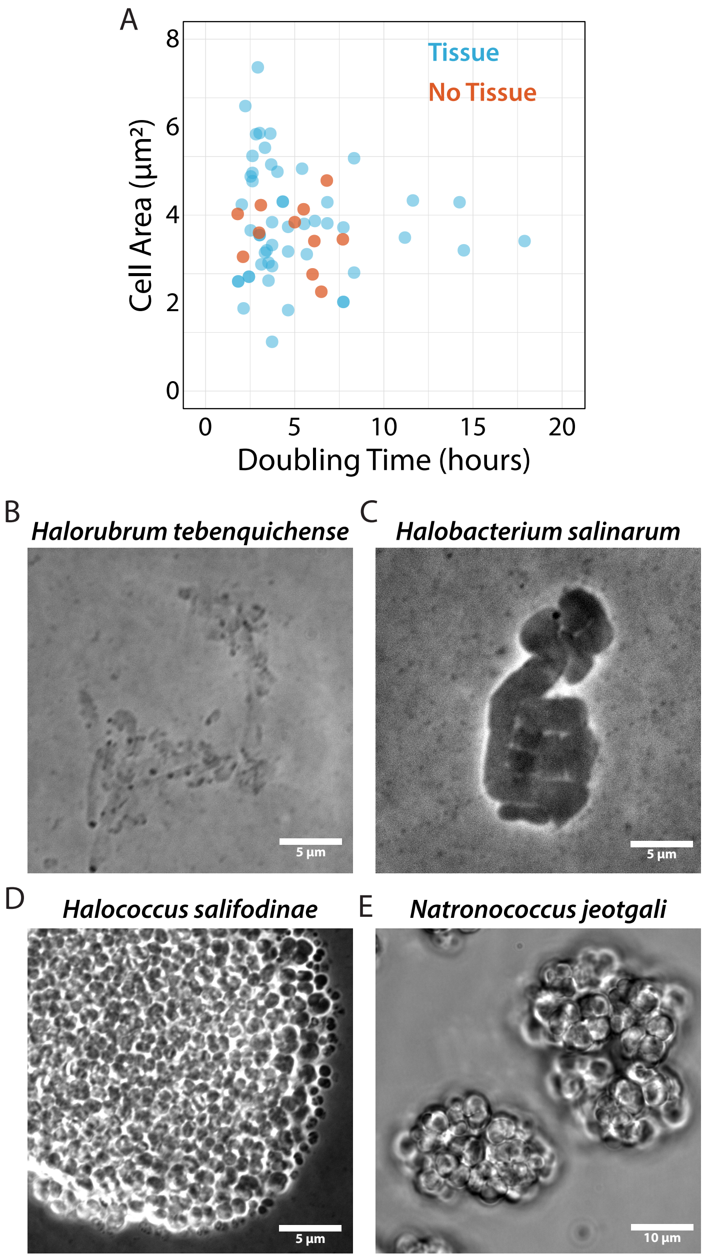
A
Cell Area (µm²)
Doubling Time (hours)
Tissue
No Tissue
0
2
4
6
8
0
5
10
15
20
B
Halorubrum tebenquichense
5 µm
C
Halobacterium salinarum
5 µm
D
Halococcus salifodinae
5 µm
E
Natronococcus jeotgali
10 µm

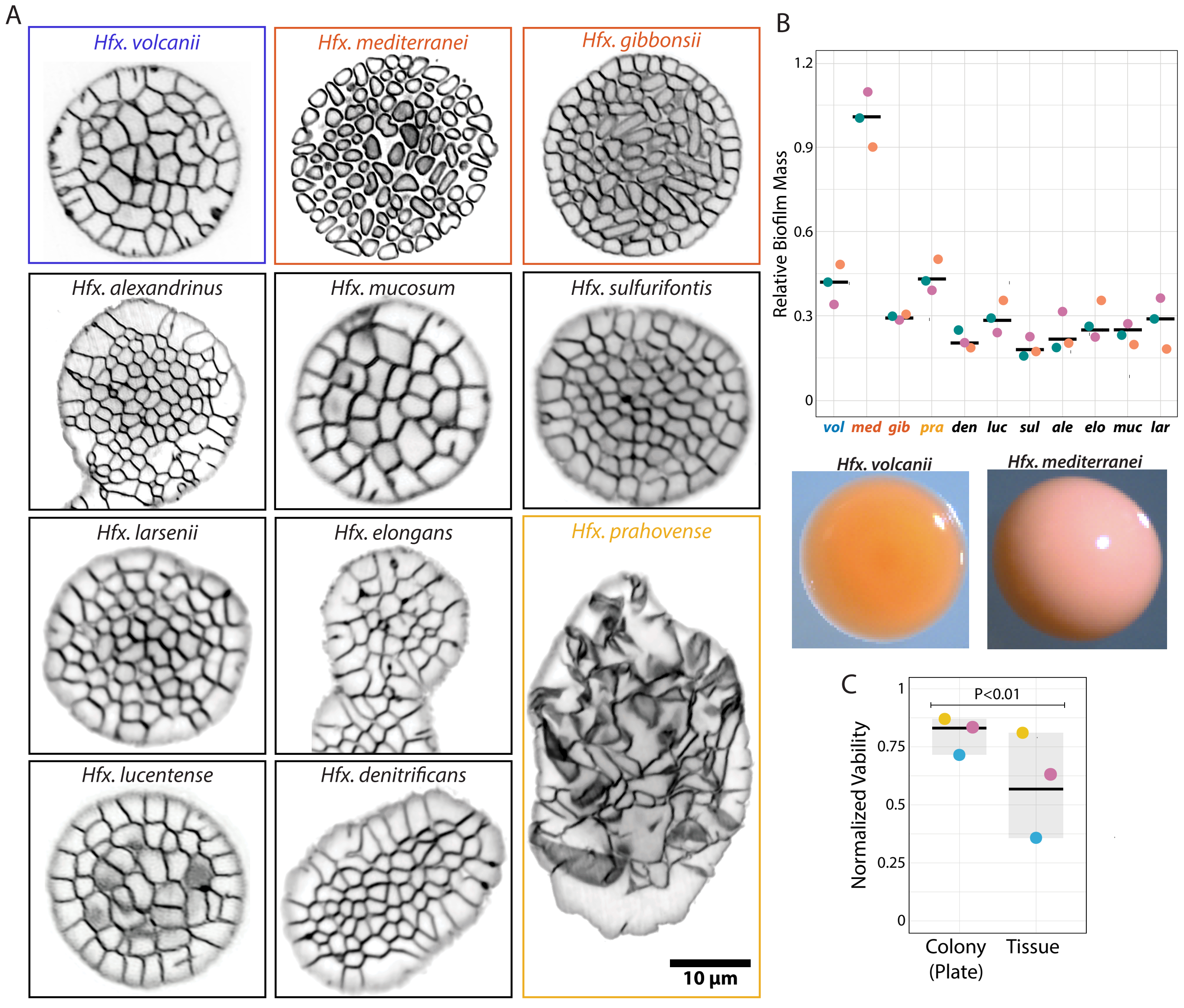

A
Hfx. volcanii
Hfx. mediterranei
Hfx. gibbonsii
Hfx. alexandrinus
Hfx. mucosum
Hfx. sulfurifontis
Hfx. larsenii
Hfx. elongans
Hfx. prahovense
Hfx. lucentense
Hfx. denitrificans
10 µm
B
Relative Biofilm Mass
1.2
0.9
0.6
0.3
0
vol med gib pra den luc sul ale elo muc lar
Hfx. volcanii
Hfx. mediterranei
C
P<0.01
Normalized Vability
1
0.75
0.5
0.25
0
Colony (Plate)
Tissue

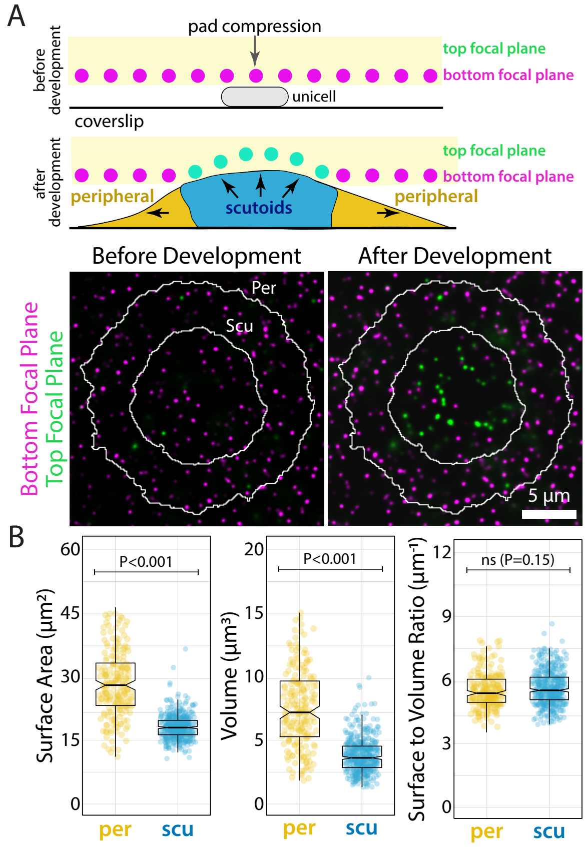
A
pad compression
before development
top focal plane
bottom focal plane
unicell
coverslip
after development
top focal plane
bottom focal plane
peripheral
scutoids
peripheral
Before Development
After Development
Bottom Focal Plane
Top Focal Plane
Per
Scu
5 µm
B
Surface Area (µm²)
P<0.001
Volume (µm³)
P<0.001
Surface to Volume Ratio (µm⁻¹)
ns (P=0.15)
per
scu
per
scu
per
scu

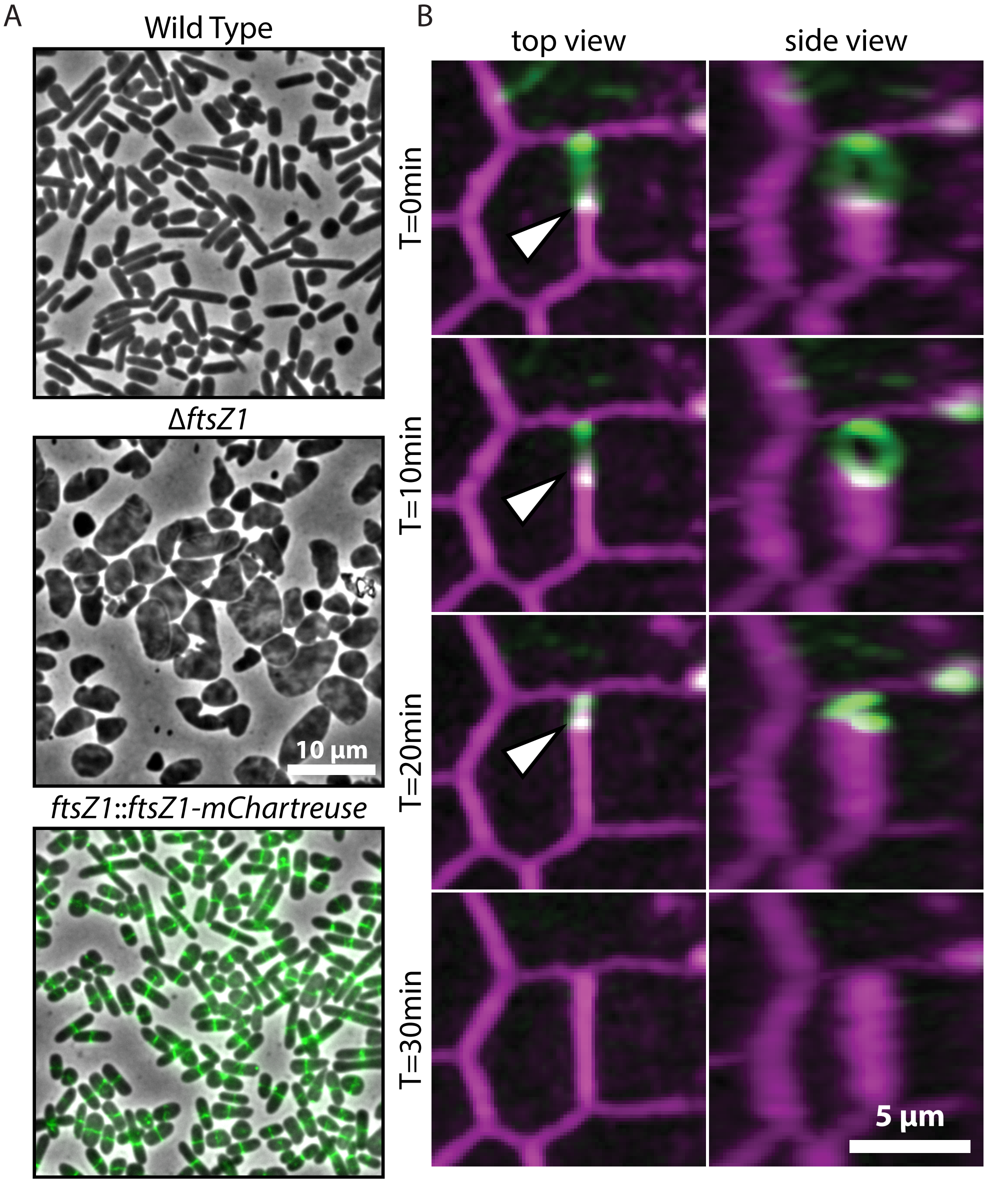

A
Wild Type
ΔftsZ1
10 µm
ftsZ1::ftsZ1-mChartreuse
B
top view
side view
T=0min
T=10min
T=20min
T=30min
5 µm

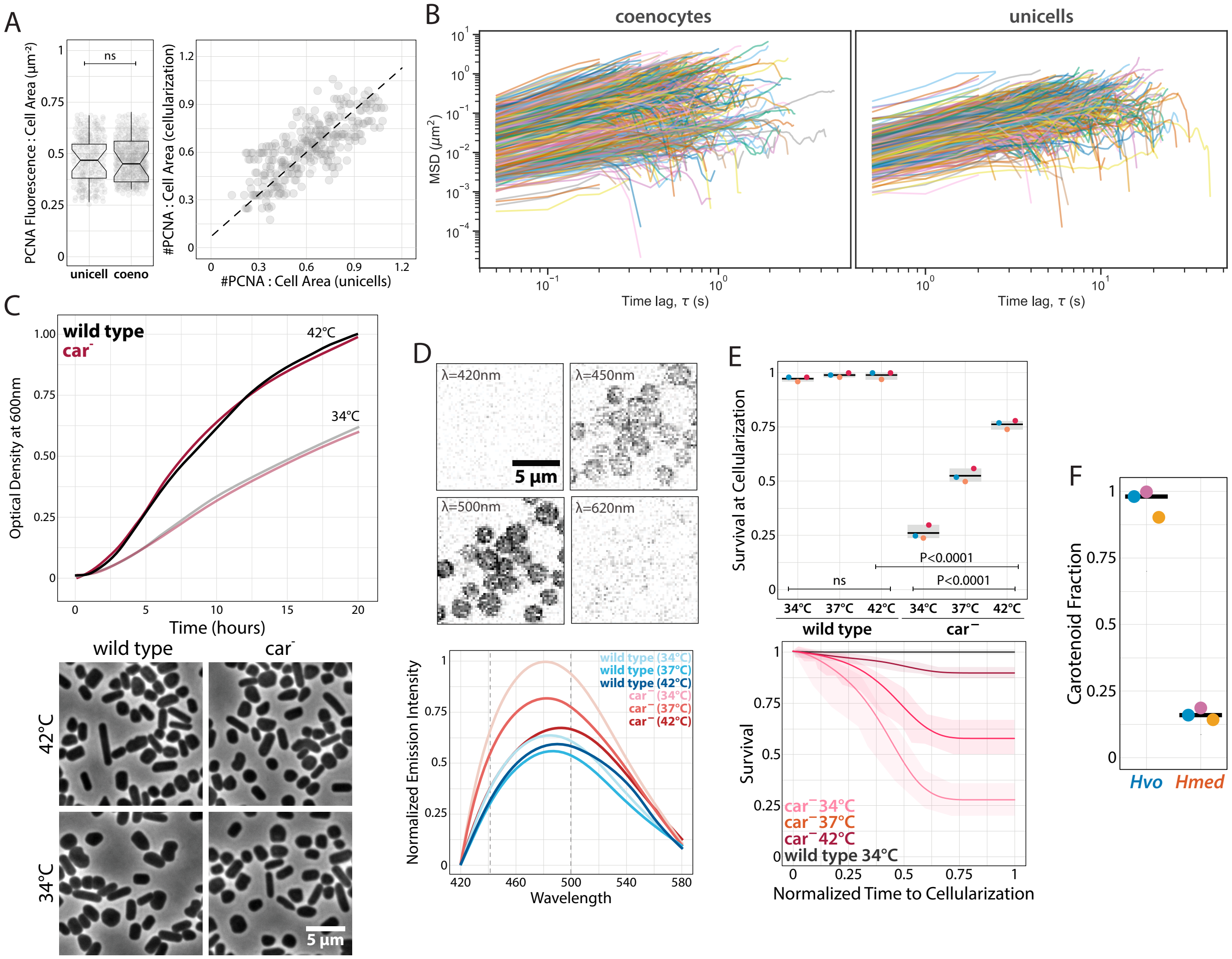
A
PCNA Fluorescence : Cell Area (µm⁻²)
ns
unicell coeno
#PCNA : Cell Area (cellularization)
#PCNA : Cell Area (unicells)
B
coenocytes
unicells
MSD (µm²)
Time lag, τ (s)
C
wild type
car-
42°C
34°C
Optical Density at 600nm
Time (hours)
wild type
car-
42°C
34°C
5 µm
D
λ=420nm
λ=450nm
λ=500nm
λ=620nm
5 µm
wild type (34°C)
wild type (37°C)
wild type (42°C)
car⁻ (34°C)
car⁻ (37°C)
car⁻ (42°C)
Normalized Emission Intensity
Wavelength
E
Survival at Cellularization
P<0.0001
ns
P<0.0001
34°C 37°C 42°C 34°C 37°C 42°C
wild type
car⁻
Survival
car⁻ 34°C
car⁻ 37°C
car⁻ 42°C
wild type 34°C
Normalized Time to Cellularization
F
Carotenoid Fraction
Hvo
Hmed

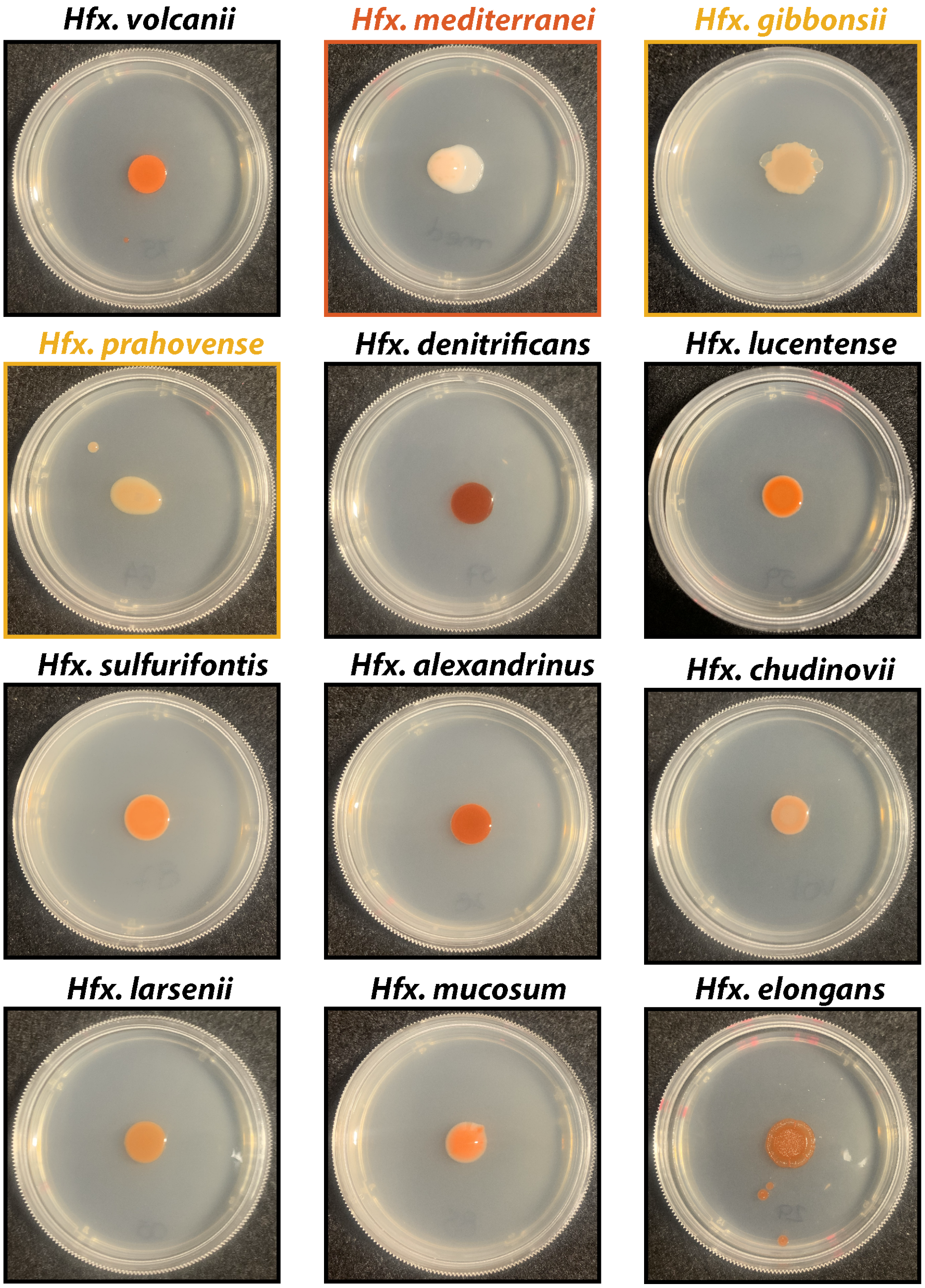

Hfx. volcanii
Hfx. mediterranei
Hfx. gibbonsii
Hfx. prahovense
Hfx. denitrificans
Hfx. lucentense
Hfx. sulfurifontis
Hfx. alexandrinus
Hfx. chudinovii
Hfx. larsenii
Hfx. mucosum
Hfx. elongans

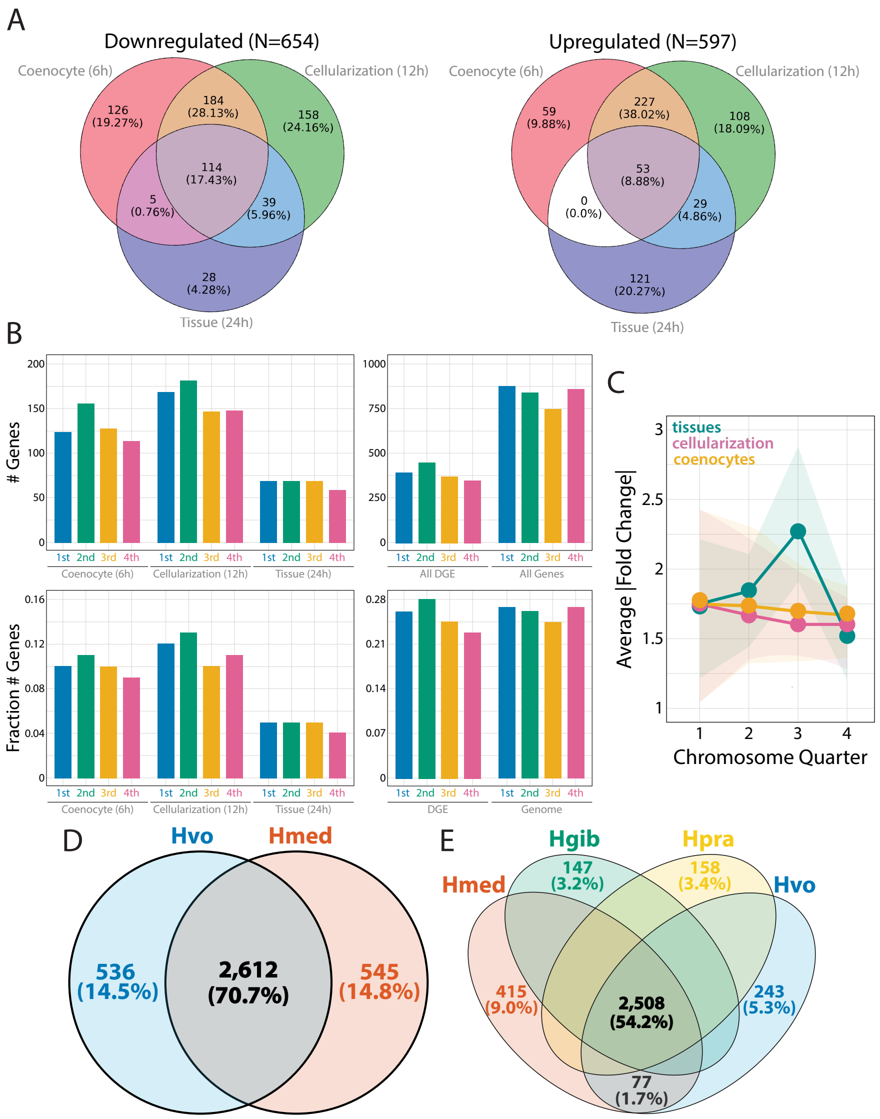

A
Downregulated (N=654)
Coenocyte (6h)
Cellularization (12h)
Tissue (24h)
126 (19.27%)
184 (28.13%)
158 (24.16%)
114 (17.43%)
5 (0.76%)
39 (5.96%)
28 (4.28%)
Upregulated (N=597)
59 (9.88%)
227 (38.02%)
108 (18.09%)
53 (8.88%)
0 (0.0%)
29 (4.86%)
121 (20.27%)
B
Genes
Fraction # Genes
1st 2nd 3rd 4th
Coenocyte (6h)
Cellularization (12h)
Tissue (24h)
All DGE
All Genes
DGE
Genome
C
tissues
cellularization
coenocytes
Average |Fold Change|
Chromosome Quarter
D
Hvo
Hmed
536 (14.5%)
2,612 (70.7%)
545 (14.8%)
E
Hgib
Hpra
Hmed
Hvo
147 (3.2%)
158 (3.4%)
415 (9.0%)
2,508 (54.2%)
243 (5.3%)
77 (1.7%)

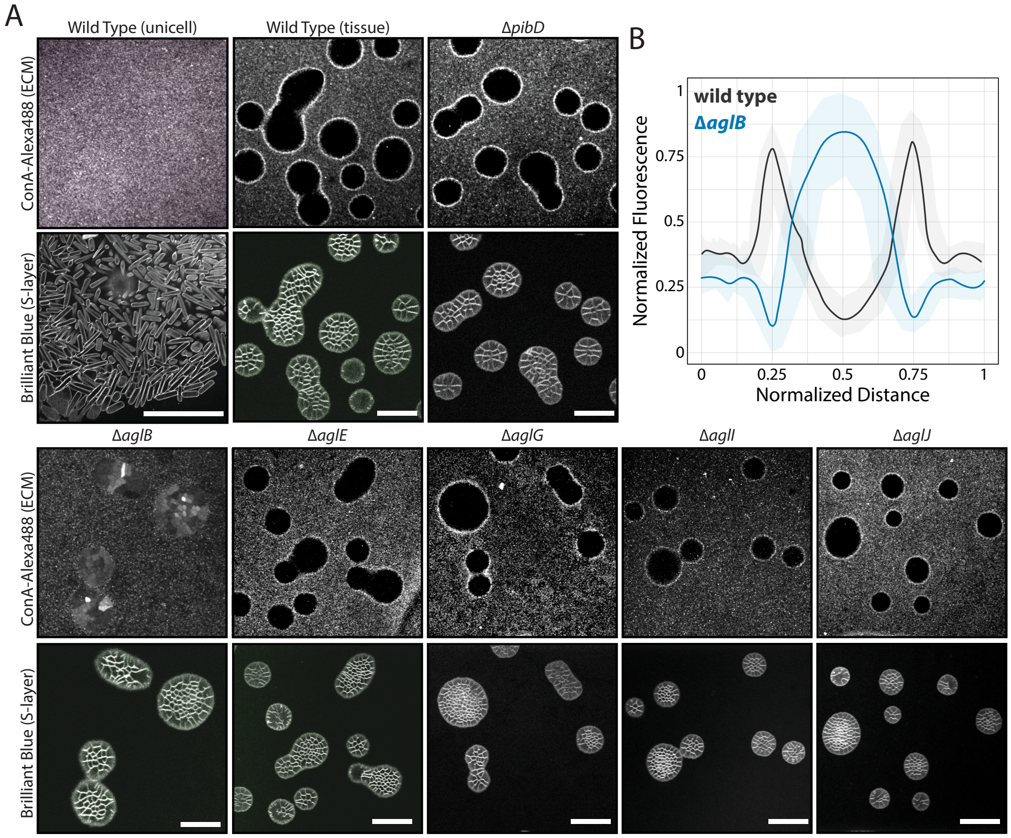

A
Wild Type (unicell)
Wild Type (tissue)
ΔpibD
ConA-Alexa488 (ECM)
Brilliant Blue (S-layer)
B
wild type
ΔaglB
Normalized Fluorescence
Normalized Distance
0
0.25
0.5
0.75
1
ΔaglB
ΔaglE
ΔaglG
ΔaglI
ΔaglJ
ConA-Alexa488 (ECM)
Brilliant Blue (S-layer)

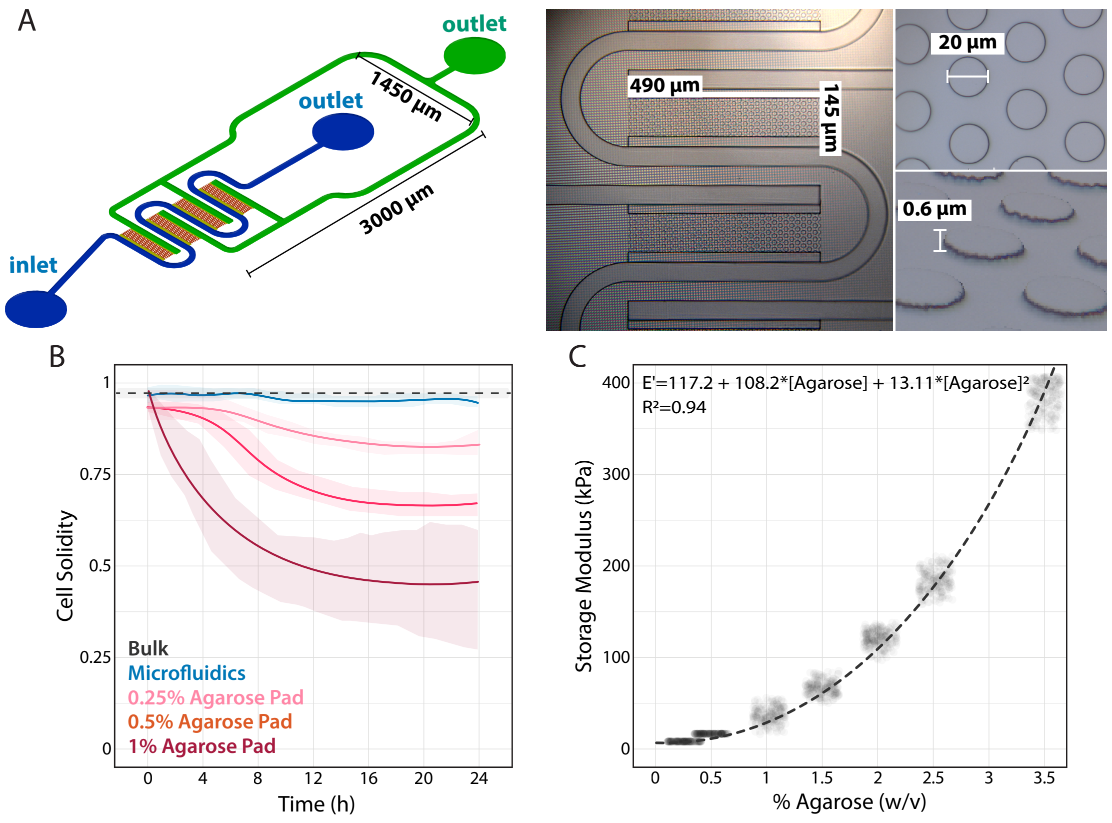
A
outlet
outlet
1450 µm
3000 µm
inlet
490 µm
145 µm
20 µm
0.6 µm
B
Cell Solidity
Time (h)
Bulk
Microfluidics
0.25% Agarose Pad
0.5% Agarose Pad
1% Agarose Pad
C
E'=117.2 + 108.2*[Agarose] + 13.11*[Agarose]²
R²=0.94
Storage Modulus (kPa)
% Agarose (w/v)

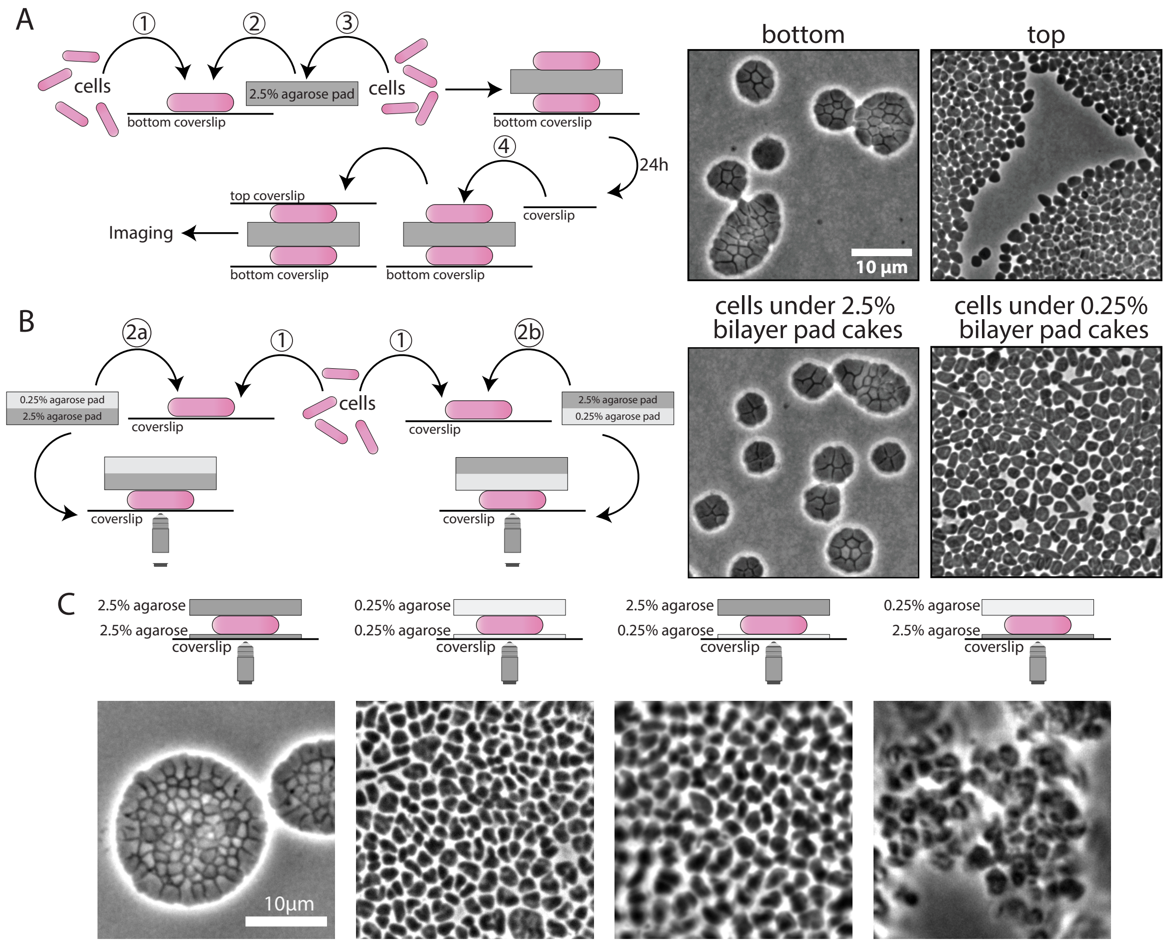

A
①
②
③
cells
2.5% agarose pad
cells
bottom coverslip
bottom coverslip
④
24h
top coverslip
coverslip
Imaging
bottom coverslip
bottom coverslip
bottom
top
10 µm
B
2a
①
①
2b
0.25% agarose pad
2.5% agarose pad
cells
coverslip
coverslip
2.5% agarose pad
0.25% agarose pad
coverslip
coverslip
cells under 2.5% bilayer pad cakes
cells under 0.25% bilayer pad cakes
C
2.5% agarose
2.5% agarose
coverslip
0.25% agarose
0.25% agarose
coverslip
2.5% agarose
0.25% agarose
coverslip
0.25% agarose
2.5% agarose
coverslip
10µm

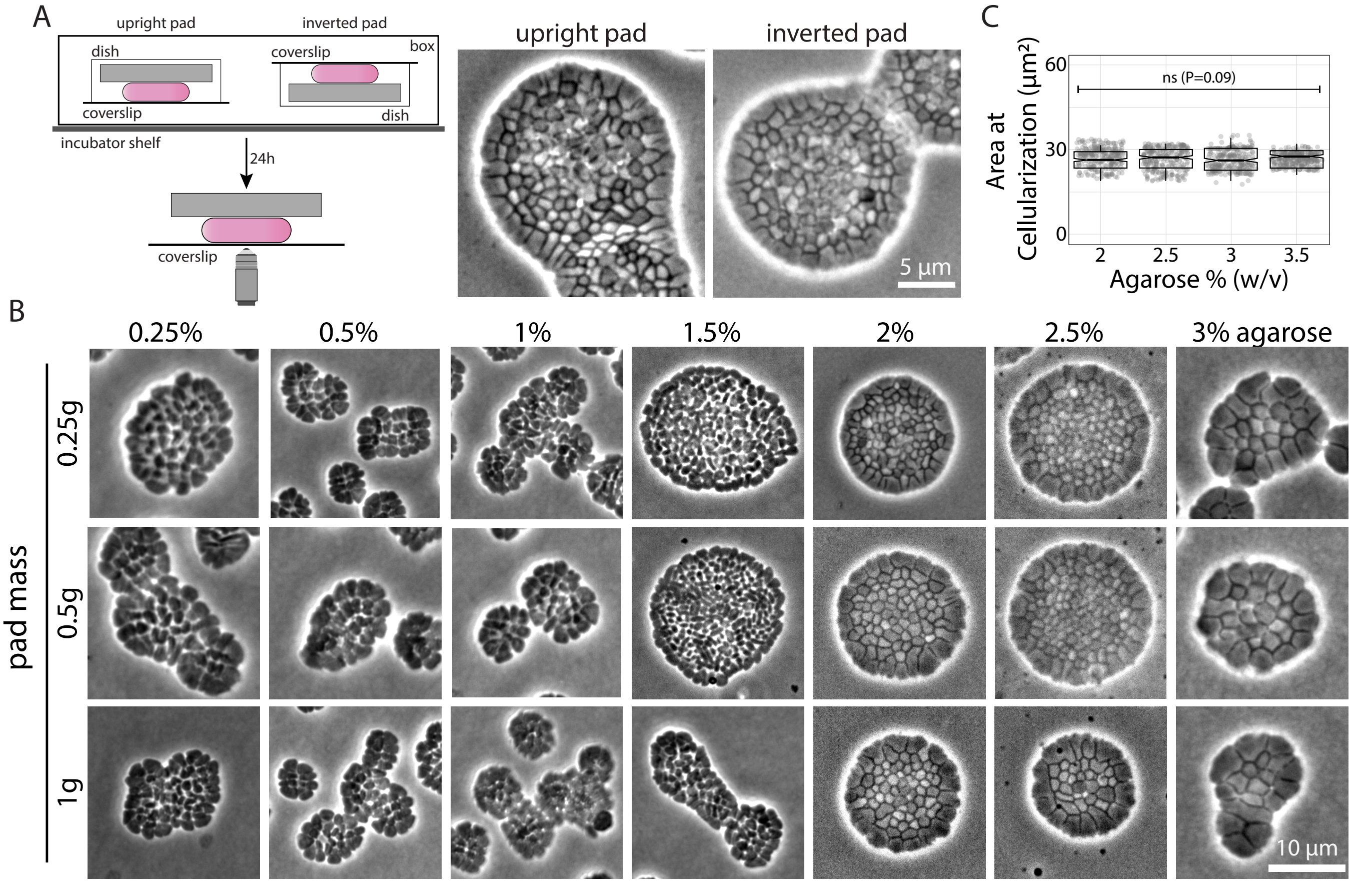
A
upright pad
inverted pad
dish
box
coverslip
coverslip
dish
incubator shelf
24h
coverslip
upright pad
inverted pad
5 µm
C
60
ns (P=0.09)
Area at
Cellularization (µm²)
30
0
2
2.5
3
3.5
Agarose % (w/v)
B
0.25%
0.5%
1%
1.5%
2%
2.5%
3% agarose
pad mass
0.25g
0.5g
1g
10 µm

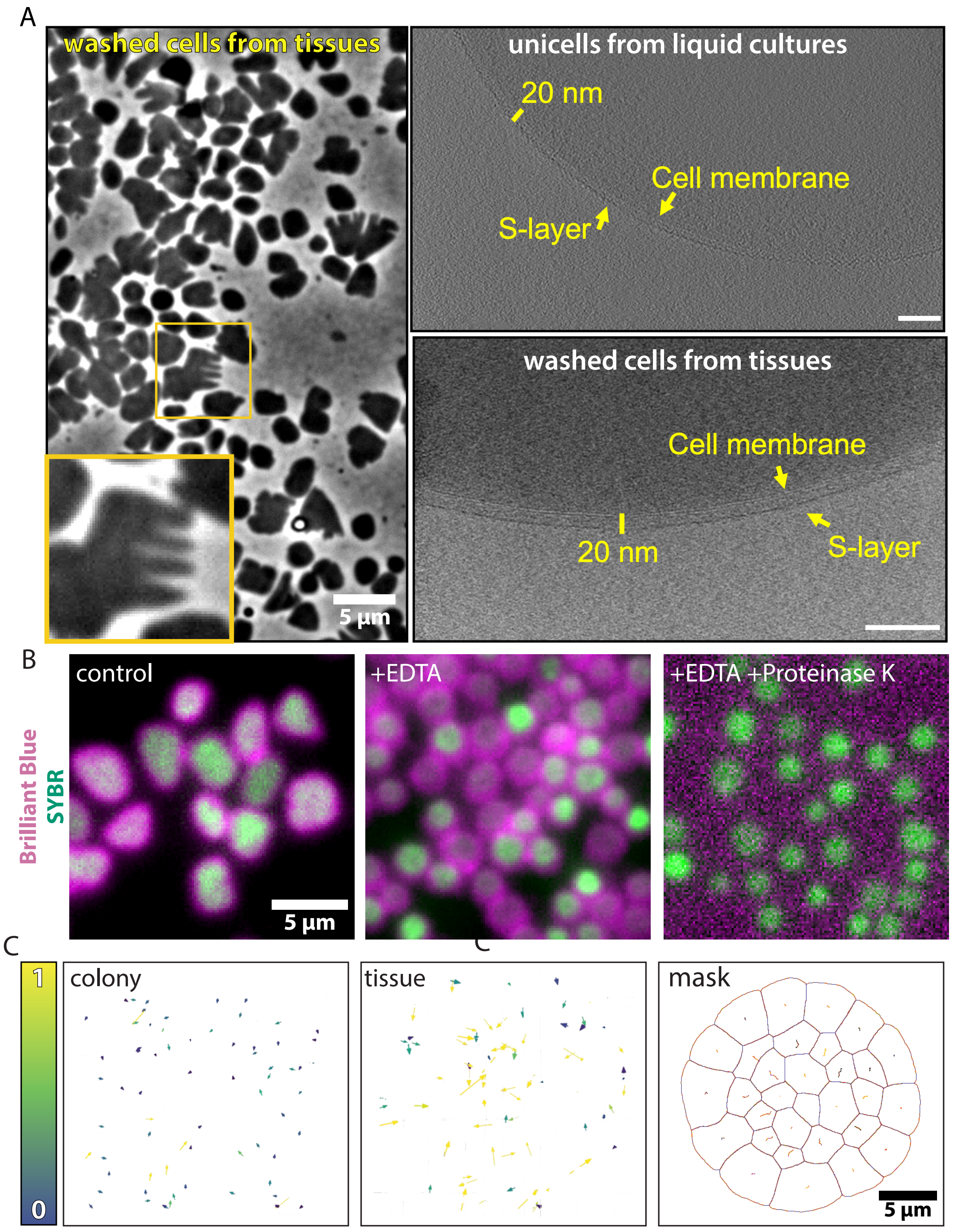
A
washed cells from tissues
5 µm
unicells from liquid cultures
20 nm
Cell membrane
S-layer
washed cells from tissues
Cell membrane
20 nm
S-layer
B
Brilliant Blue
SYBR
control
5 µm
+EDTA
+EDTA +Proteinase K
C
1
0
colony
tissue
mask
5 µm

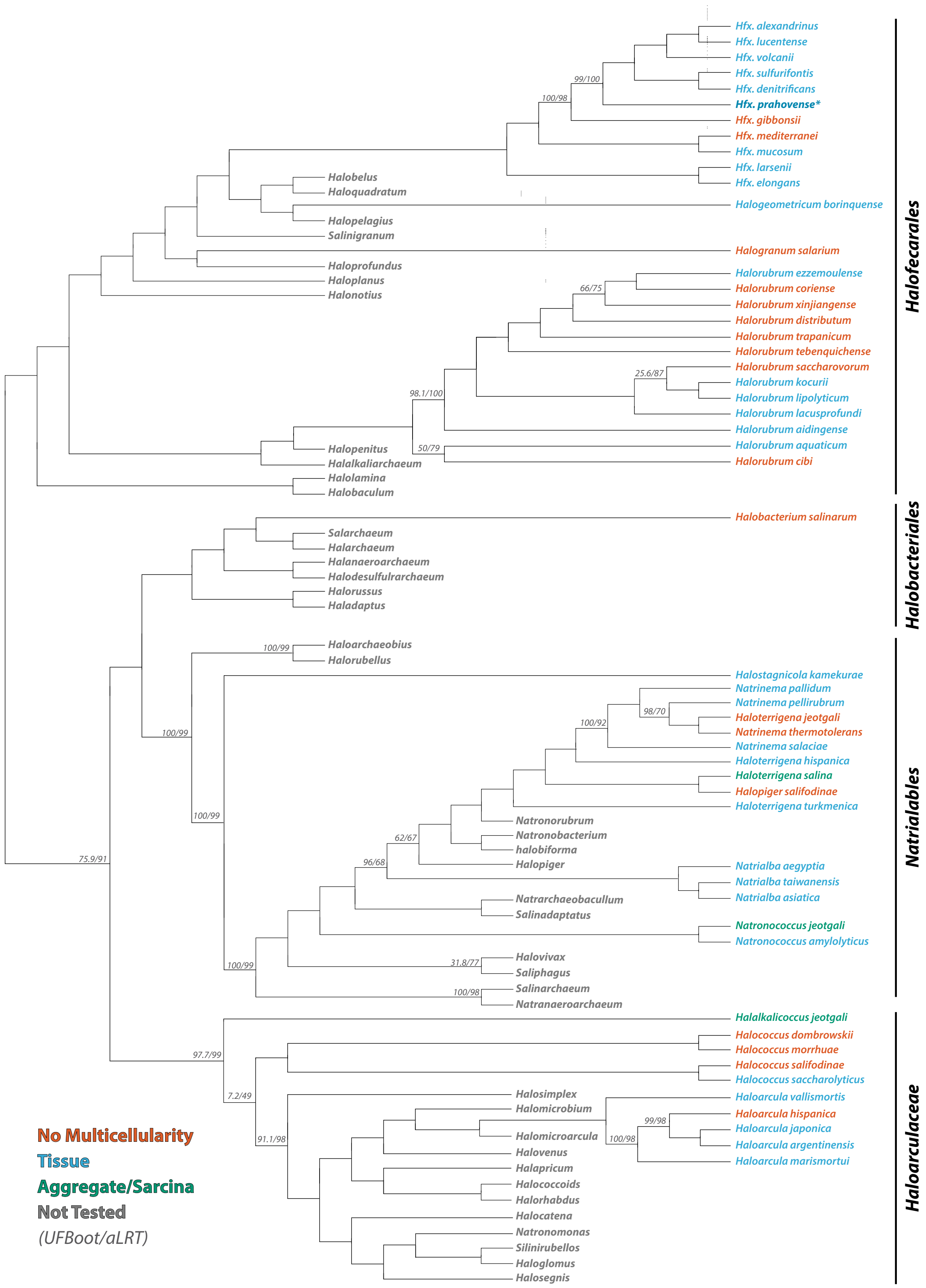

Hfx. alexandrinus
Hfx. lucentense
Hfx. volcanii
Hfx. sulfurifontis
Hfx. denitrificans
99/100
Hfx. prahovense*
100/98
Hfx. gibbonsii
Hfx. mediterranei
Hfx. mucosum
Hfx. larsenii
Hfx. elongans
Halobelus
Haloquadratum
Halogeometricum borinquense
Halopelagius
Salinigranum
Halogranum salarium
Haloprofundus
Haloplanus
Halonotius
Halorubrum ezzemoulense
66/75
Halorubrum coriense
Halorubrum xinjiangense
Halorubrum distributum
Halorubrum trapanicum
Halorubrum tebenquichense
Halorubrum saccharovorum
25.6/87
Halorubrum kocurii
Halorubrum lipolyticum
98.1/100
Halorubrum lacusprofundi
Halorubrum aidingense
Halopenitus
50/79
Halorubrum aquaticum
Halalkaliarchaeum
Halorubrum cibi
Halolamina
Halobaculum
Halofecarales
Halobacterium salinarum
Salarchaeum
Halarchaeum
Halanaeroarchaeum
Halodesulfurarchaeum
Halorussus
Haladaptus
Halobacteriales
Haloarchaeobius
100/99
Halorubellus
Halostagnicola kamekurae
Natrinema pallidum
Natrinema pellirubrum
98/70
100/92
Haloterrigena jeotgali
Natrinema thermotolerans
Natrinema salaciae
100/99
Haloterrigena hispanica
Haloterrigena salina
Halopiger salifodinae
Haloterrigena turkmenica
100/99
Natronorubrum
Natronobacterium
62/67
halobiforma
Halopiger
96/68
Natrialba aegyptia
75.9/91
Natrialba taiwanensis
Natrialba asiatica
Natrarchaeobacullum
Salinadaptatus
Natronococcus jeotgali
Natronococcus amylolyticus
Natrialables
Halovivax
31.8/77
Saliphagus
100/99
Salinarchaeum
100/98
Natranaeroarchaeum
Halalkalicoccus jeotgali
Halococcus dombrowskii
Halococcus morrhuae
97.7/99
Halococcus salifodinae
Halococcus saccharolyticus
Halosimplex
7.2/49
Haloarcula vallismortis
Halomicrobium
Haloarcula hispanica
99/98
Haloarcula japonica
91.1/98
Halomicroarcula
100/98
Haloarcula argentinensis
Halovenus
Haloarcula marismortui
Halapricum
Halococcoids
Halorhabdus
Halocatena
Natronomonas
Silinirubellos
Haloglomus
Halosegnis
Haloarculaceae
No Multicellularity
Tissue
Aggregate/Sarcina
Not Tested
(UFBoot/aLRT)

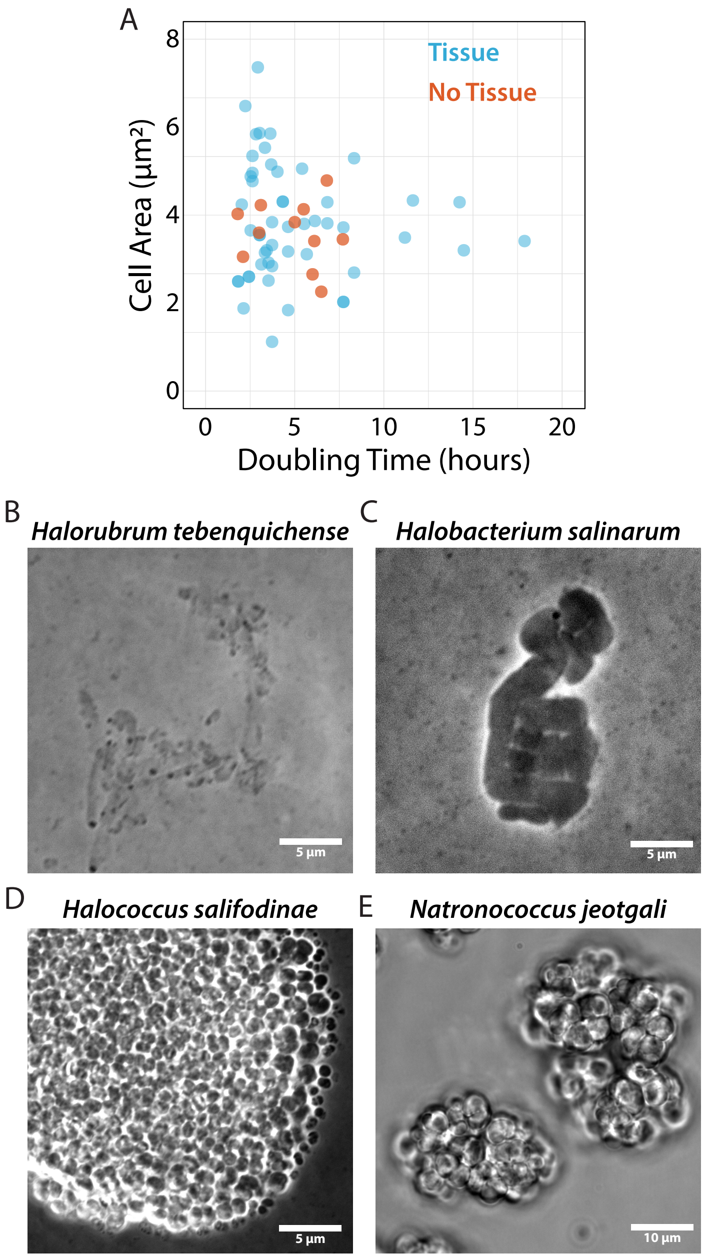
A
Tissue
No Tissue
Cell Area (µm²)
Doubling Time (hours)
0
2
4
6
8
0
5
10
15
20
B
Halorubrum tebenquichense
5 µm
C
Halobacterium salinarum
5 µm
D
Halococcus salifodinae
5 µm
E
Natronococcus jeotgali
10 µm

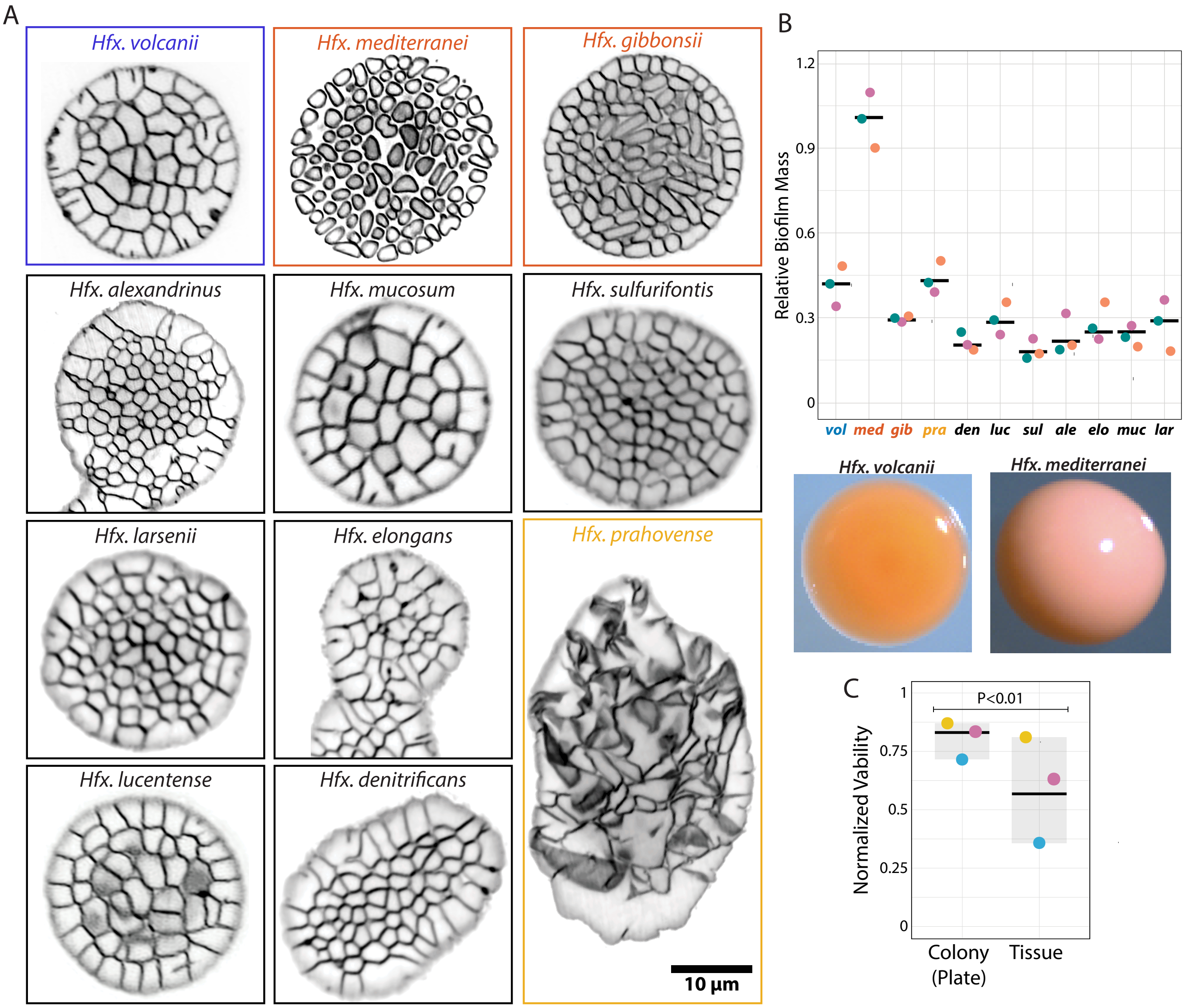

A
Hfx. volcanii
Hfx. mediterranei
Hfx. gibbonsii
Hfx. alexandrinus
Hfx. mucosum
Hfx. sulfurifontis
Hfx. larsenii
Hfx. elongans
Hfx. prahovense
Hfx. lucentense
Hfx. denitrificans
10 µm
B
Relative Biofilm Mass
1.2
0.9
0.6
0.3
0
vol med gib pra den luc sul ale elo muc lar
Hfx. volcanii
Hfx. mediterranei
C
Normalized Vability
1
0.75
0.5
0.25
0
P<0.01
Colony (Plate)
Tissue

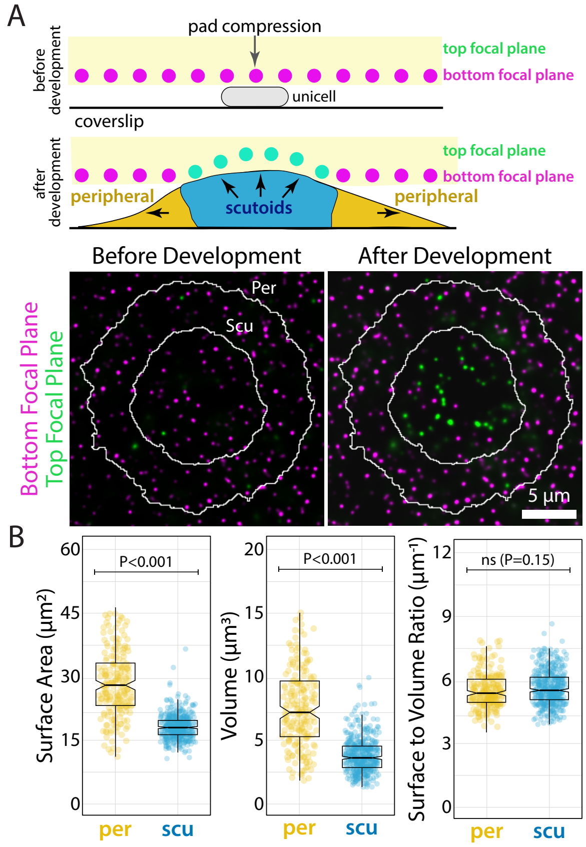
A
pad compression
before development
top focal plane
bottom focal plane
unicell
coverslip
after development
top focal plane
bottom focal plane
peripheral
scutoids
peripheral
Before Development
After Development
Bottom Focal Plane
Top Focal Plane
Per
Scu
5 µm
B
Surface Area (µm²)
P<0.001
Volume (µm³)
P<0.001
Surface to Volume Ratio (µm⁻¹)
ns (P=0.15)
per
scu
per
scu
per
scu

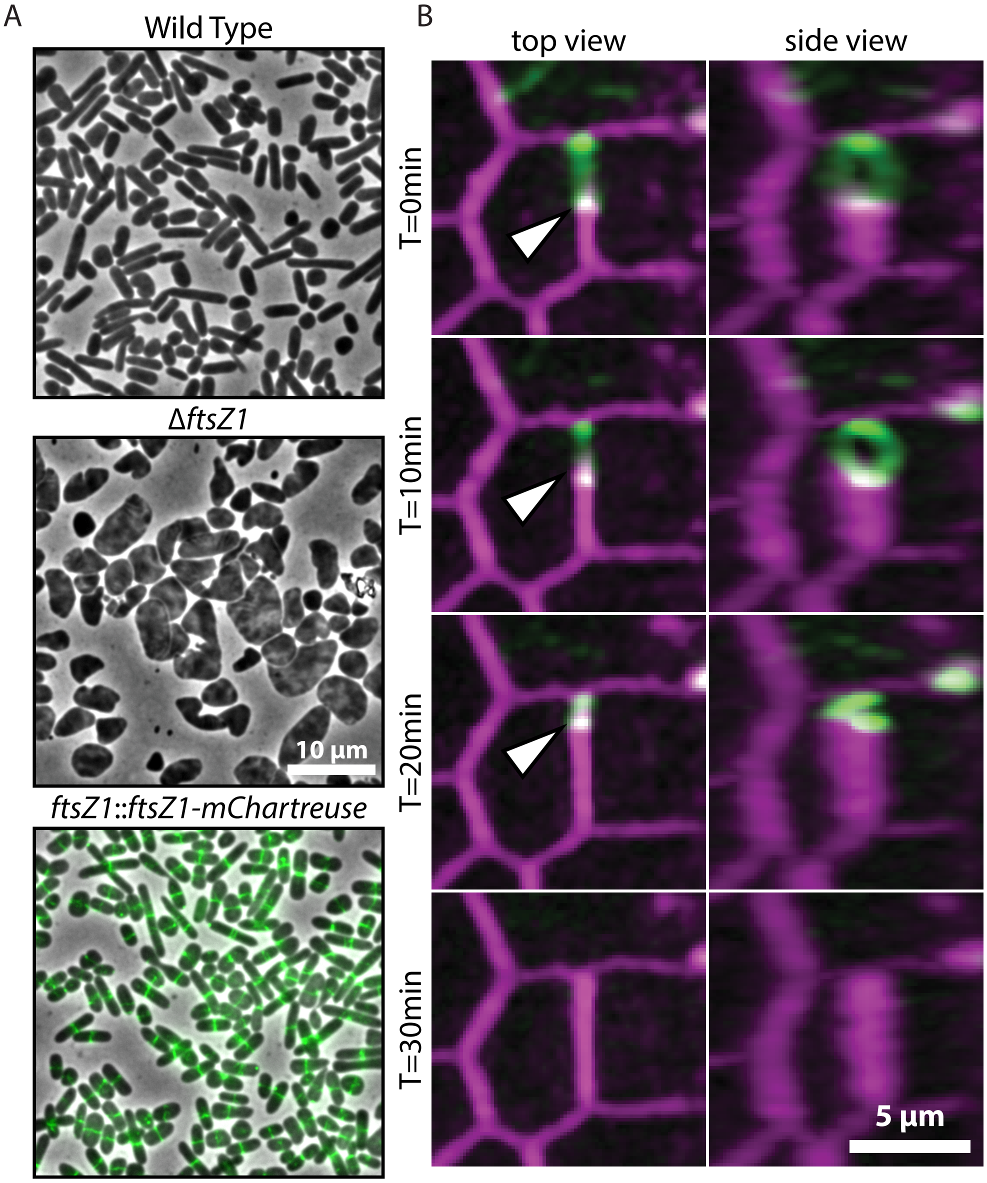
A
Wild Type
ΔftsZ1
10 µm
ftsZ1::ftsZ1-mChartreuse
B
top view
side view
T=0min
T=10min
T=20min
T=30min
5 µm

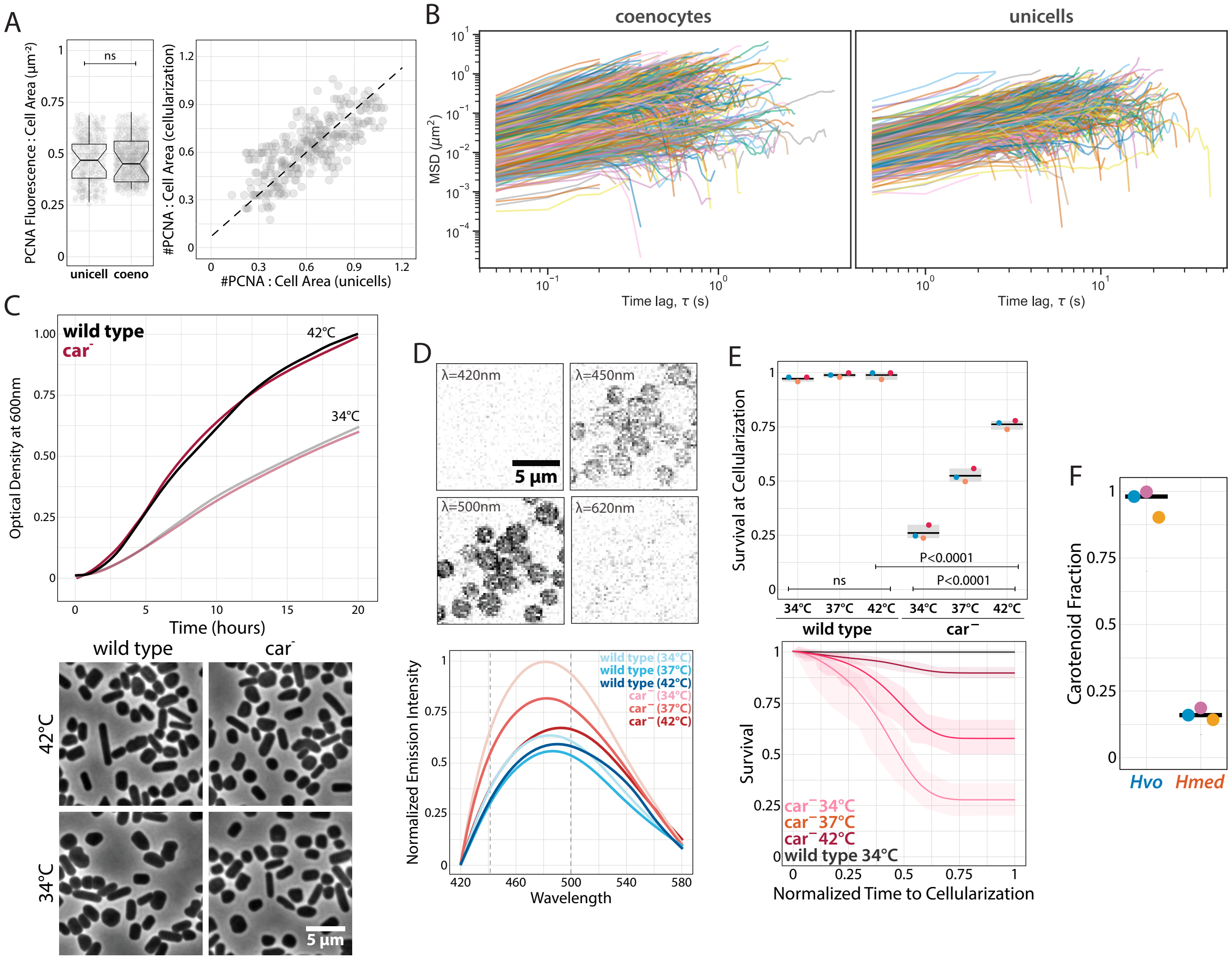
A
B
C
D
E
F
ns
PCNA Fluorescence : Cell Area (µm⁻²)
unicell
coeno
#PCNA : Cell Area (cellularization)
#PCNA : Cell Area (unicells)
coenocytes
unicells
MSD (µm²)
Time lag, τ (s)
wild type
car⁻
42°C
34°C
Optical Density at 600nm
Time (hours)
5 µm
λ=420nm
λ=450nm
λ=500nm
λ=620nm
Normalized Emission Intensity
Wavelength
wild type (34°C)
wild type (37°C)
wild type (42°C)
car⁻ (34°C)
car⁻ (37°C)
car⁻ (42°C)
Survival at Cellularization
P<0.0001
P<0.0001
34°C
37°C
42°C
Survival
car⁻ 34°C
car⁻ 37°C
car⁻ 42°C
wild type 34°C
Normalized Time to Cellularization
Carotenoid Fraction
Hvo
Hmed

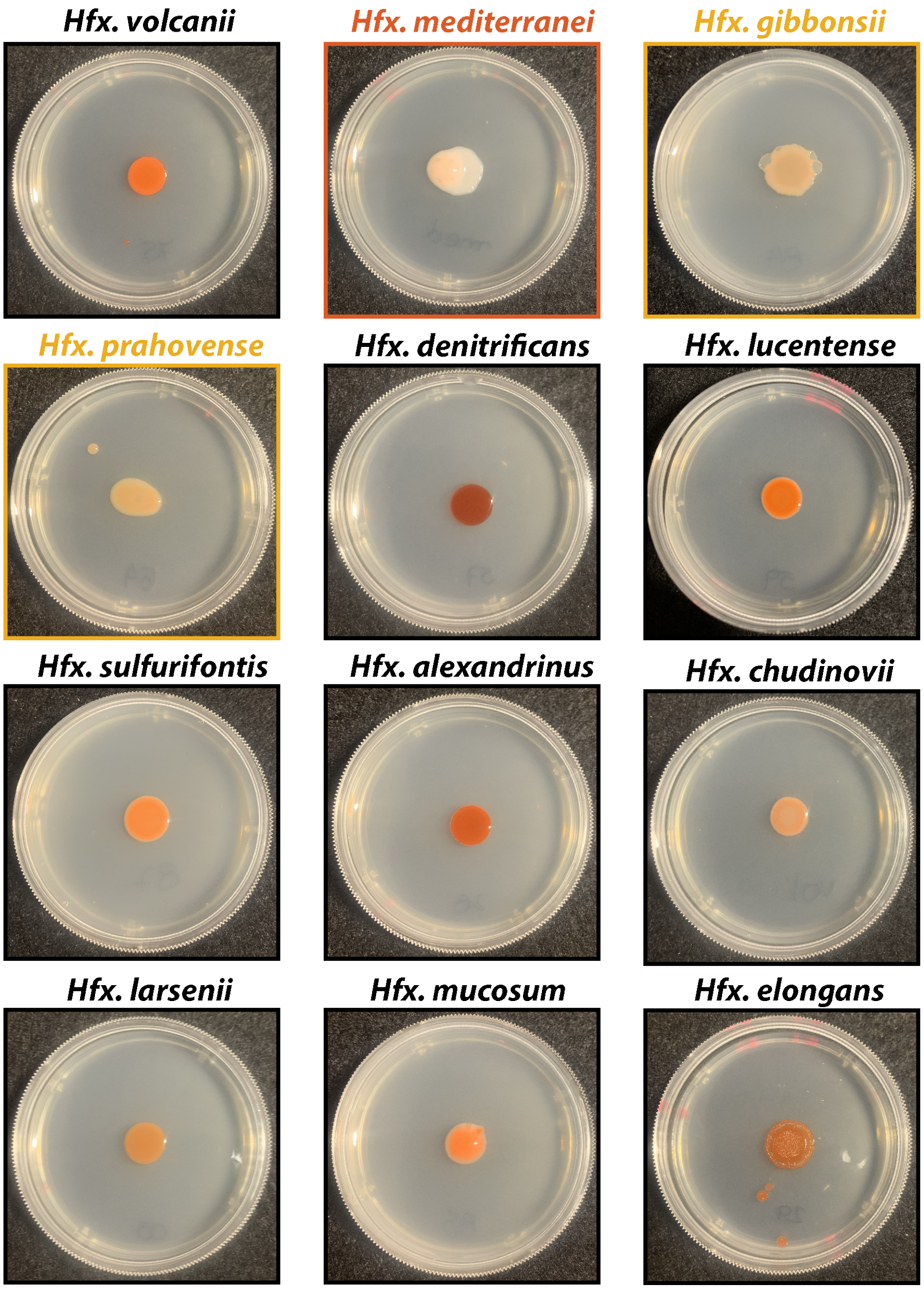

Hfx. volcanii
Hfx. mediterranei
Hfx. gibbonsii
Hfx. prahovense
Hfx. denitrificans
Hfx. lucentense
Hfx. sulfurifontis
Hfx. alexandrinus
Hfx. chudinovii
Hfx. larsenii
Hfx. mucosum
Hfx. elongans

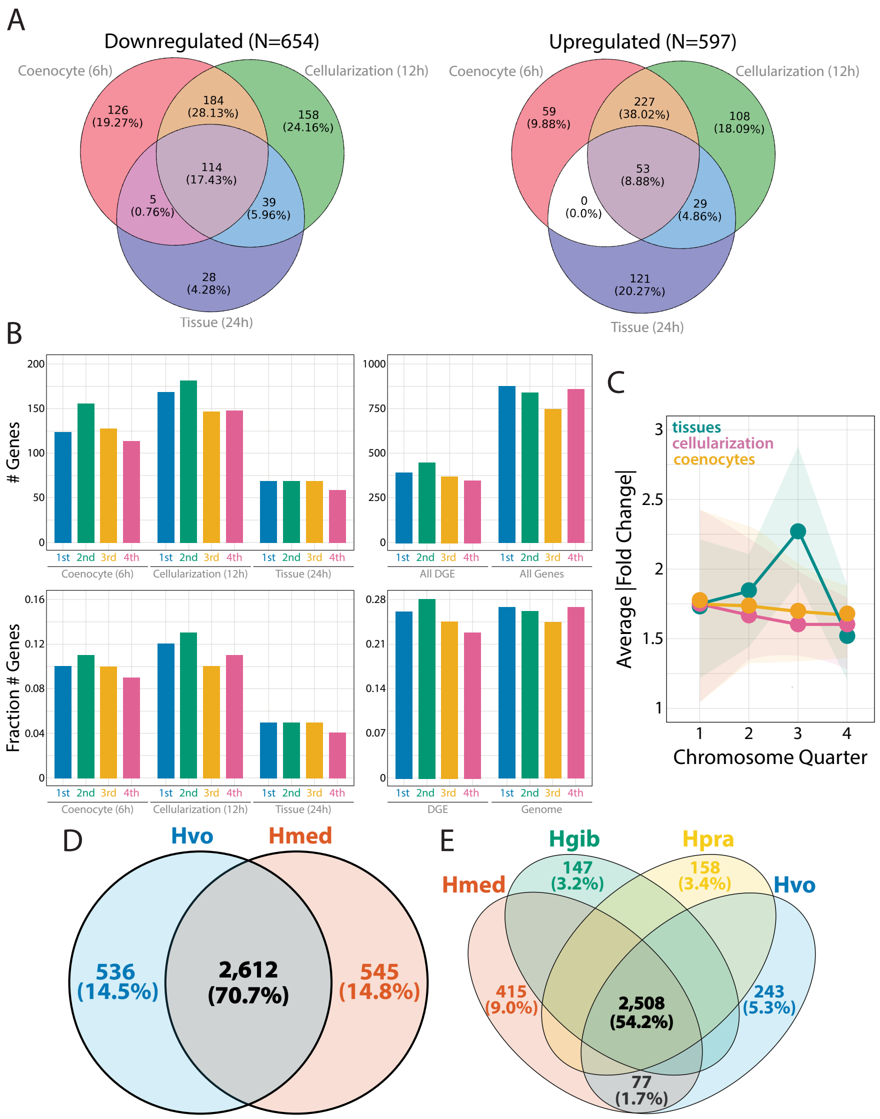

A
Downregulated (N=654)
Coenocyte (6h)
Cellularization (12h)
126 (19.27%)
184 (28.13%)
158 (24.16%)
114 (17.43%)
5 (0.76%)
39 (5.96%)
28 (4.28%)
Tissue (24h)
Upregulated (N=597)
Coenocyte (6h)
Cellularization (12h)
59 (9.88%)
227 (38.02%)
108 (18.09%)
53 (8.88%)
0 (0.0%)
29 (4.86%)
121 (20.27%)
Tissue (24h)
B
Genes
Fraction # Genes
1st 2nd 3rd 4th
Coenocyte (6h)
Cellularization (12h)
Tissue (24h)
All DGE
All Genes
DGE
Genome
C
tissues
cellularization
coenocytes
Average |Fold Change|
Chromosome Quarter
D
Hvo
Hmed
536 (14.5%)
2,612 (70.7%)
545 (14.8%)
E
Hgib
Hpra
Hmed
Hvo
147 (3.2%)
158 (3.4%)
415 (9.0%)
2,508 (54.2%)
243 (5.3%)
77 (1.7%)

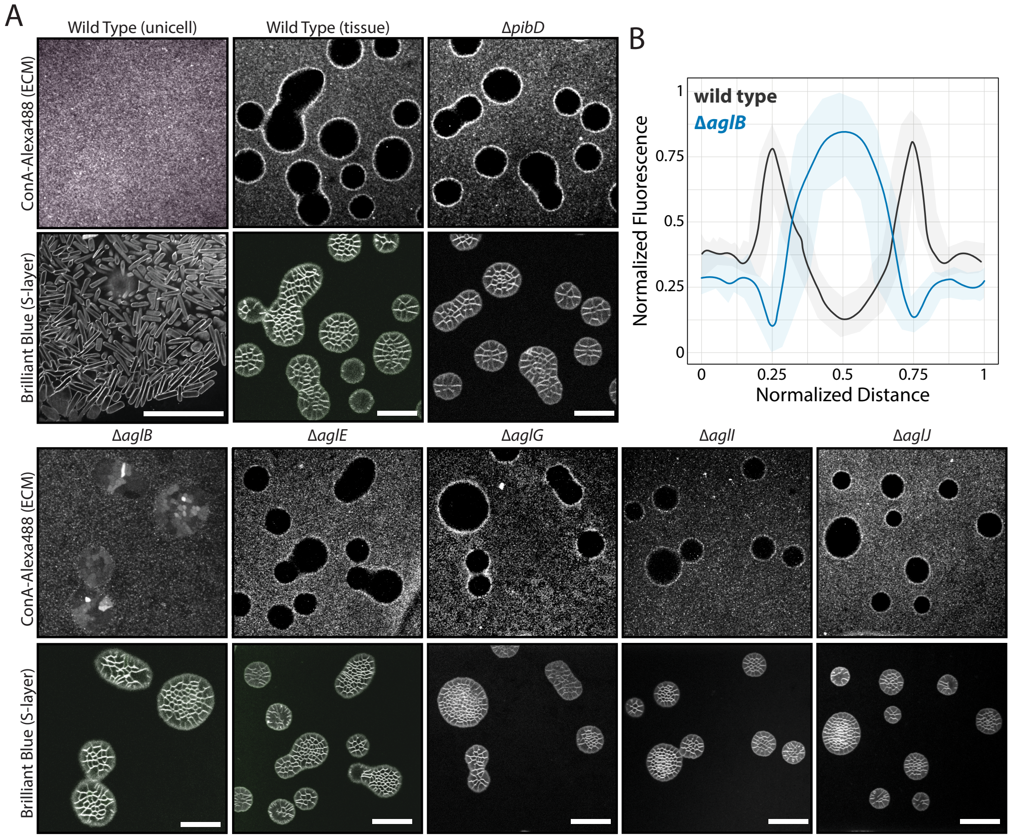
A
Wild Type (unicell)
Wild Type (tissue)
ΔpibD
ConA-Alexa488 (ECM)
Brilliant Blue (S-layer)
B
wild type
ΔaglB
Normalized Fluorescence
Normalized Distance
0
0.25
0.5
0.75
1
ΔaglB
ΔaglE
ΔaglG
ΔaglI
ΔaglJ
ConA-Alexa488 (ECM)
Brilliant Blue (S-layer)
